# Dereplication of Bioactive *Agave* Saponin Fractions: The Hidden Saponins

**DOI:** 10.1021/acs.jafc.4c02308

**Published:** 2024-06-05

**Authors:** Ana M. Simonet, Alexandra G. Durán, Francisco A. Macías

**Affiliations:** Allelopathy Group, Department of Organic Chemistry, Institute of Biomolecules (INBIO), Campus de Excelencia Internacional (ceiA3), School of Science, University of Cadiz, C/República Saharaui n° 7, Puerto Real, Cadiz 11510, Spain

**Keywords:** *Agave macroacantha*, *A. colorata*, *A. parryi*, *A. parrasana*, dereplication, steroidal saponin, coloratoside, HMAI, bioherbicide, apiose

## Abstract

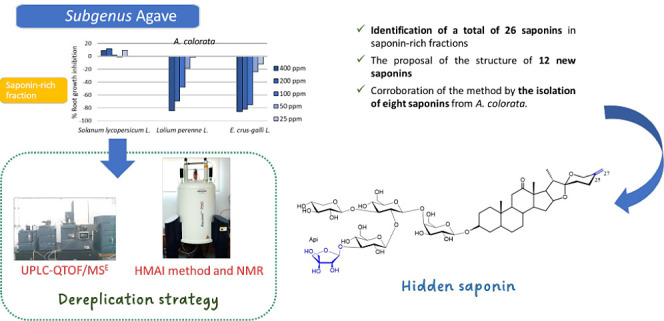

The good phytotoxicity and selectivity against weeds versus tomato or cress make saponin-rich fractions from *Agave macroacantha*, *A. colorata*, *A. parryi*, and *A. parrasana* attractive candidates as bioherbicides. The saponin contents have only previously been reported for *A. macroacantha*, and as a consequence, simultaneous dereplication has been performed on saponin-rich fractions from the other plants by mass spectrometry (MS) and nuclear magnetic resonance (NMR) spectroscopy. This strategy enables the identification of a total of 26 saponins, 14 of which have been described previously and 12 of which are proposed as new saponins. They include isomers and a new sugar chain with a β-d-apiofuranose unit. The method is corroborated by the isolation of eight dereplicated saponins from *A. colorata*.

## Introduction

Saponins are secondary metabolites that have a broad range of biological activities, and these compounds have been known since ancient times.^1,2^ Despite the wide range of applications of saponins and the increasing research interest in them, the purification and isolation of these metabolites remain a challenge.^3^ For this reason, saponin-rich fractions, rather than pure saponins, are the products that are commercially available for exploitation in the food, cosmetics, agricultural, and pharmaceutical sectors. Examples of extracts found in the market include *Yucca* extracts and saponins from tea, quinoa, and *Dioscorea*.^4,5^

The phytotoxic effects of steroidal saponin-rich fractions from different *Agave* species have recently been evaluated,^6^ and it was found that fractions belonging to the subgenus *Agave* were the most active. These fractions showed good activity profiles on two problematic weed species, namely, *Lolium perenne* L. (perenne ryegrass) and *Echinochloa crus-galli* L. (barnyard-grass), with IC_50_ values below 160 and 55 ppm (Table 1), respectively. It is worth noting that significant activity was not observed on *Solanum lycopersicum* L. (tomato) or *Lepidium sativum* L. (cress). This selectivity highlights the potential of saponin-rich fractions for use as natural herbicides, and suitable fractions could be obtained by exploiting the byproducts of the tequila industry.^7^

**Table 1:** IC_50_ Values (ppm) of Saponin-Rich Fractions of *Agave* Species Dereplicated in this Study on Cultivars and Weeds on Root Growth Inhibition^a^

	species evaluated				
saponin-rich fraction	*Solanum lycopersicum* L.	*Lepidium sativum* L.	*Lactuca sativa* L.	*Lolium perenne* L.	*Echinocloa crus-galli* L.
*A. parrasana*	n.a.	n.a.	364.1	147.6	59.54
*A. macroacantha*	n.a.	n.a.	368.1	147.9	55.54
*A. colorata*	n.a.	n.a.	548.1	110.0	68.04
*A. parryi*	n.a.	n.a.	361.9	158.6	58.62
Logran	60.14	n.a.	n.a.	61.56	n.a.

a The commercial herbicide Logran® was used as positive control. Adapted from Durán et al. *Agromomy*, **2021**, *11*, 2404. Copyright 2021 Creative Common CC By license.^6^

The bioactive fractions of *Agave macroacantha*, *A. colorata*, *A. parryi*, and *A. parrasana* showed similar profiles in a UPLC-QTOF/MS^E^ analysis, but the saponin contents have not previously been described for the latter three species.

More than a hundred saponins have been isolated from the genus *Agave*.^8^ Regarding the monosaccharides found in the sugar chains of the saponins, units of d-glucose, d-galactose, d-xylose, and l-rhamnose have been described. Moreover, the structural features found in steroidal aglycones include chiral centers at C-5 and C-25, the opening of the ring F in the case of furostanic saponins, or different functionalizations that include unsaturations or oxygenations. The characteristic fragmentation of the sugar chains in the mass spectra and the current published NMR data set for steroidal saponins enable us to propose a method for the dereplication of saponin-rich fractions^9^ using a combination of UPLC-QTOF/MS^E^ and NMR spectroscopy.

In a previous study, the bioactive fraction of *A. macroacantha* was dereplicated, and this led to the identification of five main saponins, four of which had the same sugar chain.^10^ The bioactive fractions of *A. colorata*, *A. parryi*, and *A. parrasana* contain several sugar chains, and the NMR spectra were therefore more complicated. In the study reported here, a method for the dereplication of saponins is proposed to identify the bioactive components in these fractions.

## Materials and Methods

### General Experimental Procedures

Optical rotations were measured on a JASCO *p*-2000 polarimeter using methanol as the solvent. Exact masses were measured on a UPLC-QTOF ESI (Waters Xevo G2, Manchester, UK) high-resolution mass spectrometer (HRESI-TOFMS). The 1D and 2D NMR spectra were recorded on an Agilent INOVA-600 spectrometer equipped with a 5 mm helium-cooled cryoprobe or on Bruker AVANCE NEO 500 MHz and Bruker AVANCE NEO 700 MHz spectrometers with a 5 mm helium-cooled cryoprobe. The^1^H and^13^C NMR spectra were recorded on samples dissolved in pyridine-*d*_5_ (Merck, Darmstadt, Germany) or MeOH-*d*_4_ (Eurisotop Saint Aubin, France) at room temperature. The chemical shifts are given on the δ scale and are referred to the residual pyridine signals (δ_H_ 8.70, 7.55, 7.18 and δ_C_ 149.84, 135.60, 123.48). ^1^H NMR spectra of fractions dissolved in MeOH-*d*_4_ were used to monitor the isolation processes.

Acetic acid and *n*-butanol were supplied by Panreac Química S.A. (Castellar del Vallés, Barcelona, Spain). Methanol, *n*-hexane, acetone, ethyl acetate, and chloroform were obtained from VWR International (Radnor, PA, USA). TLC silica 60 F_254_ and TLC Si gel F_254_S RP-18 plates were purchased from Merck (Darmstadt, Germany) and were used to monitor the isolation processes. The compounds were visualized by spraying the plate with H_2_SO_4_/H_2_O/AcOH (4:16:80 v/v/v). LiChroprep RP-18 (40–63 μm) from Merck (Darmstadt, Germany) was used for vacuum column chromatography for the first fractionation. Further purification was carried out using silica gel 0.060–0.200 (60 Å) from Acros Organics (Geel, Belgium) and preparative TLC silica gel 60 F_254_ (0.50 mm) and TLC silica gel RP-18 F_254_S (0.25 mm) supplied by Merck (Darmstadt, Germany).

### Plant Material

Leaves of *A. macroacantha*, *A. colorata*, *A. parryi*, and *A. parrasana* were supplied in November 2017 by Desert City S.L. (CIF B86691474, Madrid, Spain, GPS coordinates 40.59897539554237, – 3.5823863738311497), and saponin-rich fractions from *n*-butanol:water extracts were prepared by the method reported by Durán et al.^6^ Reference samples of powdered plant materials and *n*-butanol extracts are available in our laboratory and are labeled as DC2017-M14, DC2017-M08, DC2017-M06, and DC2017-M24, respectively.

*A. colorata* leaves (DC2021-M08) of the same specimen were supplied in November 2021 by the same company for saponin isolation.

### Extraction and Isolation of *A. Colorata*

Dried leaves of *A. colorata* (DC2021-M08; 18 g) were moistened in water (36 mL) for 2 h, and the same volume of *n*-butanol was then added. The solution was kept at room temperature for 24 h for the extraction process. The same volume of water was added a second time, and the extract was stirred slowly for a further 24 h. The two phases were separated, and the solvent was removed from the *n*-butanol layer under vacuum to yield 864 mg (4.7%) of crude extract. This residue was further purified by vacuum column chromatography with reverse phase silica gel (RP-18) with different ratios of H_2_O:MeOH [100% H_2_O, H_2_O:MeOH (1:1), H_2_O:MeOH (3:7), H_2_O:MeOH (1:4), and 100% MeOH] to obtain the saponin-rich fraction (425 mg, 49%, H_2_O:MeOH [3:7]). This fraction was purified by column chromatography on silica gel using as the eluent the lower layer of a biphasic solvent CHCl_3_:MeOH:H_2_O in a ratio of 13:7:2.5. The fractions obtained were rechromatographed under the same conditions to obtain 10 fractions with different NMR profiles. Fraction D (23 mg) was subjected to preparative TLC on silica gel 60 F254 0.5 mm plates using the organic phase of a mixture of CHCl_3_:MeOH:acetone:H_2_O (65:35:5:15) as the eluent to obtain fraction D2 (6 mg), which contained saponins with S4 as the sugar chain, and fraction D4 (8.5 mg), which contained saponins with S5Api as the sugar chain. Fraction E (14.5 mg) was subjected to preparative TLC on silica gel 60 F254 0.5 mm plates using ethyl acetate:AcOH:H_2_O (7:2:2) as the eluent to obtain a fraction containing saponins with S5Xyl as the sugar chain (4 mg). Fraction H (10.3 mg) was subjected to preparative TLC on silica gel 60 F254 0.5 mm plates using ethyl acetate:AcOH:H_2_O (7:2:2) as the first eluent and, after drying, *n*-butanol:AcOH:water (5:1:5) as the second eluent to obtain a fraction with saponins that had S5Rha as the sugar chain (6 mg). The four fractions were chromatographed on TLC silica gel RP-18 using a MeOH:H_2_O:AcOH ratio of 80:20:1 to obtain coloratoside A (**1**) (3.2 mg), coloratoside B (**2**) (2 mg), coloratoside C (**3**) (1.3 mg), coloratoside D (**4**) (0.5 mg), coloratoside E (**5**) (0.5 mg), TTS 14 (**6**) (1.6 mg), hecogenin-3-*O*-{β-d-xylopyranosyl-(1→3)-*O*-β-d-glucopyranosyl-(1→2)-*O*-[β-d-xylopyranosyl-(1→3)]-*O*-β-d-glucopyranosyl-(1→4)-*O*-β-d-galactopyranoside} (**7**) (1.6 mg), and agameroside E (**8**) (3.7 mg).

#### Coloratoside A (**1**)

[α]_Na_^25^ – 20.3 (*c* 0.32, MeOH); ^1^H and ^13^C NMR, see Tables 2 and 3; HRESIMS (negative ion mode) *m*/*z* 1225.5485 [M + CH_3_COO**^–^**]**^–^** (calcd for C_56_H_89_O_29_, 1225.5490); MS^E^*m*/*z* 1179 [M – H]^−^, 1047 [M – H – 132]^−^, 885 [M – H – 162–132]^−^, 753 [M – H – 162–132 × 2]^−^, 591 [M – H – 132 × 2–162 × 2]^−^

**Table 2:** ^13^C and ^1^H NMR Data (*J* in Hz) for the Aglycone Moieties of Compounds **1**–**5** (pyridine-*d*_5_)^a^,^b^

	coloratoside A (1)		coloratoside B (2)		coloratoside C (3)		coloratoside D (4)		coloratoside E (5)	
	δ_C_	δ_H_	δ_C_	δ_H_	δ_C_	δ_H_	δ_C_	δ_H_	δ_C_	δ_H_
1_ax_	36.7	0.70 ddd (14, 14, 4)	36.7	0.69 ddd (13, 13, 4)	36.7	0.69 ddd (14, 14, 4)	36.7	0.69 ddd (13, 13, 4)	36.7	0.71 ddd (14, 14, 4)
1_eq_		1.29 ddd (14, 3, 3)		1.29 (o)		1.28 br d (14)		1.28 ddd (13, 4, 4)		1.30 (o)
2_ax_	29.7	1.54 (o)	29.7	1.54 dddd (13, 13, 11, 5)	29.7	1.52 dddd (14, 14, 13, 4)	29.7	1.54 (o)	29.7	1.56 dddd (14, 13, 11, 5)
2_eq_		1.98 br d (14)		1.99 br d (13)		1.96 br d (13)		1.98 br d (13)		1.99 br d (13)
3	77.2	3.85 dddd (11, 11, 5, 5)	77.2	3.86 dddd (12, 11, 5, 5)	77.1	3.85 dddd (11, 11, 5, 5)	77.2	3.86 dddd (11, 11, 5, 5)	77.2	3.86 dddd (11, 11, 5, 5)
4_ax_	34.7	1.32 (o)	34.7	1.33 ddd (12, 12, 12)	34.7	1.29 (o)	34.7	1.31 (o)	34.7	1.33 (o)
4_eq_		1.77 br d (12)		1.79 br d (12)		1.77 br d (12)		1.77 br d (12)		1.78 dd (13, 5)
5	44.5	0.82 (o)	44.5	0.83 m	44.5	0.83 m	44.5	0.83 (o)	44.5	0.84 m
6	28.7	1.09 m (2H)	28.7	1.11 m (2H)	28.7	1.10 m (2H)	28.7	1.11 m (2H)	28.7	1.12 m (2H)
7_ax_	31.9	0.74 dddd (12, 12, 12, 5)	31.8	0.75 dddd (13, 12, 12, 5)	31.8	0.75 dddd (13, 12, 12, 5)	31.8	0.76 dddd (13, 13, 13, 5)	31.8	0.77 dddd (13, 12, 12, 4)
7_eq_		1.53 (o)		1.54 br d (13)		1.53 br d (13)		1.55 (o)		1.55 (o)
8	34.4	1.72 dddd (12, 11, 11, 4)	34.4	1.73 dddd (12, 11, 11, 4)	34.4	1.72 dddd (11, 11, 11, 4)	34.4	1.73 dddd (11, 11, 11, 4)	34.4	1.74 (o)
9	55.6	0.88 ddd (14, 11, 5)	55.6	0.88 ddd (13, 11, 5)	55.5	0.88 ddd (14, 11, 5)	55.6	0.88 ddd (12, 12, 5)	55.6	0.89 ddd (13, 11, 5)
10	36.3	-	36.4	-	36.3	-	36.4	-	36.4	-
11_ax_	38.1	2.35 dd (14, 14)	38.1	2.35 dd (14, 13)	38.1	2.34 dd (14, 14)	38.1	2.35 dd (14, 14)	38.0	2.36 dd (14, 13)
11_eq_		2.21 dd (14, 5)		2.21 dd (14, 5)		2.20 dd (14, 5)		2.21 dd (14, 5)		2.22 dd (14, 5)
12	212.8	-	212.8	-	212.7	-	212.7	-	212.8	-
13	55.4	-	55.4	-	55.4	-	55.4	-	55.4	-
14	56.0	1.34 (o)	56.0	1.35 ddd (13, 11, 6)	55.9	1.34 ddd (13, 11, 5)	56.0	1.34 ddd (12, 12, 5)	56.0	1.36 m
15_a_	31.5	1.57 (o)	31.5	1.58 ddd (13, 13, 7)	31.5	1.57 ddd (13, 12, 6)	31.5	1.57 ddd (13, 12, 7)	31.5	1.59 ddd (13, 13, 7)
15_b_		2.08 ddd (13, 7, 7)		2.07 ddd (13, 7, 7)		2.07 ddd (12, 7, 7)		2.07 ddd (12, 7, 7)		2.08 ddd (13, 7, 7)
16	79.8	4.47 ddd (9, 7, 7)	80.1	4.47 ddd (7, 7, 7)	80.1	4.46 ddd (8, 7, 7)	80.1	4.46 ddd (7, 7, 7)	80.1	4.47 ddd (7, 7, 7)
17	54.4	2.74 dd (9, 7)	54.4	2.74 dd (9, 7)	54.4	2.74 dd (9, 7)	54.4	2.74 dd (9, 7)	54.4	2.75 dd (9, 7)
18	16.2	1.06 s	16.2	1.06 s	16.2	1.06 s	16.2	1.06 s	16.1	1.07 s
19	11.8	0.64 s	11.8	0.65 s	11.8	0.63 s	11.8	0.64 s	11.8	0.67 s
20	42.7	1.90 dq (7, 7)	42.6	1.92 dq (7, 7)	42.7	1.91 dq. (7, 7)	42.6	1.91 dq (7, 7)	42.6	1.92 dq. (7, 7)
21	14.0	1.34 d (7)	14.0	1.30 d (7)	14.0	1.30 d (7)	14.0	1.30 d (7)	13.9	1.30 d (7)
22	109.4	-	109.6	-	109.6	-	109.6	-	109.6	-
23_ax_	31.8	1.69 br dd (14, 8)	33.3	1.80 ddd (13, 13, 5)	33.3	1.79 ddd (13, 13, 5)	33.3	1.78 (o)	33.3	1.80 ddd (13, 13, 5)
23_eq_		1.61 ddd (14, 3, 3)		1.73 br dd (13, 6)		1.72 ddd (13, 5, 2)		1.72 (o)		1.73 br dd (13, 6)
24_ax_	29.3	1.54 (o) (2H)	29.0	2.68 br ddd (13, 13, 6)	29.0	2.69 br ddd (13, 13, 5)	29.0	2.68 br ddd (13, 13, 6)	29.0	2.69 br ddd (13, 13, 5)
24_eq_				2.23 br d (13)		2.23 br d (13)		2.23 br d (13)		2.23 br dd (13, 6)
25	30.6	1.56 (o)	144.4	-	144.4	-	144.4	-	144.3	-
26_ax_	67.0	3.46 dd (11, 11)	65.2	4.43 d (12)	65.2	4.43 d (13)	65.2	4.43 d (12)	65.1	4.43 d (12)
26_eq_		3.56 dd (11, 4)		4.02 d (12)		4.02 d (13)		4.01 d (12)		4.02 d (12)
27a	17.4	0.67 d (6)	108.9	4.80 br s	108.9	4.80 s	108.9	4.80 s	108.9	4.80 br s
27b				4.76 br s		4.77 s		4.76 s		4.77 br s

a Assignments were confirmed by ^1^H–^1^H-COSY and 1D- and 2D-TOCSY, HSQC, HSQC-TOCSY, and HMBC experiments.

b o: overlapped with other signals.

**Table 3:** ^13^C and ^1^H NMR Data (*J* in Hz) of the Sugar Portions of Compounds **1**–**5** (pyridine-*d*_*5*_)^a^,^b^

	coloratoside A (1)			coloratoside B (2)			coloratoside C (3)			coloratoside D (4)			coloratoside E (5)		
	δ_C_	δ_H _(C–H)	δ_H _(OH)	δ_C_	δ_H _(C–H)	δ_H (OH)_	δ_C_	δ_H _(C–H)	δ_H (OH)_	δ_C_	δ_H _(C–H)	δ_H (OH)_	δ_C_	δ_H _(C–H)	δ_H (OH)_
		β-d-Gal			β-d-Gal			β-d-Gal			β-d-Gal			β-d-Gal	
1	102.6	4.83 d (8)	-	102.6	4.84 d (8)	-	102.5	4.85 d (8)	-	102.6	4.84 d (8)	-	102.5	4.84 d (8)	-
2	73.2	4.40 dd (8, 10)		73.2	4.40 dd (8, 9)	6.84	73.3	4.40 dd (8, 9)		73.2	4.37 dd (9, 8)	6.81	73.2	4.40 dd (10, 8)	6.92
3	75.5	4.10 br d (10)		75.6	4.10 ddd (10, 9, 3)	5.09	75.7	4.10 (o)	5.04	75.6	4.10 m	5.01 d	75.5	4.09 dd (10, 3)	5.06
4	79.8	4.57 br d (3)	-	79.9	4.58 br d (3)	-	80.0	4.58 br d (3)	-	79.8	4.58 brd (3)	-	79.5	4.57 br d (3)	-
5	75.6	3.99 dd (9, 6)	-	75.6	3.99 dd (8, 5)	-	75.5	3.99 dd (9, 6)	-	75.5	3.99 brdd (9, 5)	-	75.6	3.99 dd (9, 5)	-
6	60.8	4.20 (o)		60.8	4.20 (o)	5.98	60.7	4.20 (o)	6.00	60.8	4.21 (o)	6.01	60.8	4.21 (o)	6.01
		4.64 dd (10, 9)			4.65 dd (11, 8)			4.67 dd (9, 9)			4.65 dd (10, 9)			4.65 dd (o)	
		β-d-Glc			β-d-Glc			β-d-Glc			β-d-Glc			β-d-Glc	
1	104.9	5.15 d (8)	-	104.9	5.15 d (8)	-	105.3	5.18 d (8)	-	105.0	5.17 d (8)	-	104.9	5.16 d (8)	-
2	80.8	4.33 dd (9, 8)	-	80.8	4.34 dd (9, 8)	-	81.5	4.41 dd (8, 9)	-	80.8	4.38 dd (8, 9)	-	81.1	4.30 dd (9, 8)	-
3	87.2	4.07 dd (9, 9)	-	87.2	4.07 dd (9, 9)	-	86.8	4.16 dd (9, 9)	-	86.8	4.12 dd (9, 9)	-	87.2	4.08 dd (9, 9)	-
4	70.4	3.78 dd (9, 10)		70.5	3.78 dd (9, 9)	5.32	70.6	3.82 dd (9, 9)	5.38	70.5	3.79 dd (9, 9)	5.35	70.4	3.79 dd (9, 8)	5.34
5	77.6	3.83 ddd (10, 8, 2)	-	77.7	3.83 ddd (9, 10, 3)	-	77.7	3.87 ddd (9, 7, 3)	-	77.9	3.85 ddd (9, 9, 2)	-	77.7	3.83 ddd (8, 10, 2)	-
6	63.0	4.04 dd (11, 8)		63.0	4.04 (o)	6.70	63.1	4.04 dd (11, 7)		63.1	4.05 (o)	6.70	63.0	4.05 (o)	6.71
		4.49 dd (11, 2)			4.49 brd (10)			4.50 br d (11)			4.49 brd (12)			4.49 dd (12, 2)	
		β-d-Glc'			β-d-Glc'			β-d-Glc'			β-d-Glc'			β-d-Glc'	
1	104.4	5.49 d (8)	-	104.4	5.49 d (8)	-	105.0	5.57 d (8)	-	104.1	5.56 d (8)	-	104.4	5.48 d (8)	-
2	75.5	4.03 (o)	6.76	75.3	4.03 (o)	6.75	76.4	4.06 dd (9, 9)	6.99	75.2	4.06 ddd (9, 8, 5)	6.90 d	76.2	3.98 m	6.72
3	84.5	4.03 (o)	-	84.5	4.03 (o)	-	77.8	4.10 dd (9, 9)	-	86.9	4.02 (o)	-	83.3	4.21 dd (9, 8)	-
4	69.5	3.98 (o)		69.5	3.98 (o)	5.99	71.1	4.19 dd (9, 9)		69.3	4.03 (o)	5.98	69.3	4.10 dd (10, 9)	6.81
5	78.4	3.72 ddd (10, 5, 2)	-	78.4	3.72 ddd (10, 5, 2)	-	78.9	3.91 ddd (9, 5, 2)	-	78.4	3.87 m	-	78.6	3.76 ddd (10, 4, 2)	-
6	62.3	4.26 dd (12, 5)		62.3	4.27 br d (11)		62.6	4.35 dd (12, 5)		62.3	4.27 dd (12,5)	6.17	62.3	4.34 dd (12, 4)	5.88
		4.44 dd (12, 2)			4.44 br d (11)			4.56 br d (12)			4.51 brd (12)			4.49 dd (12, 2)	
		β-d-Xyl			β-d-Xyl			β-d-Xyl			β-d-Xyl			β-d-Xyl	
1	105.0	5.12 d (8)	-	105.0	5.13 d (8)	-	105.1	5.23 d (8)	-	105.0	5.14 d (8)	-	105.0	5.12 d (8)	-
2	75.3	3.93 dd (8, 8)	8.15	75.3	3.94 ddd (8, 9, 4)	8.16	75.2	3.95 dd (8, 9)	8.32	75.2	3.93 dd (9, 8)	7.88	75.3	3.93 dd (9, 8)	8.32
3	78.6	4.01 dd (9, 9)		78.6	4.01 dd (9, 9)		78.8	4.06 dd (9, 9)		78.5	4.04 dd (9, 9)		78.5	3.99 dd (9, 9)	
4	70.7	4.09 ddd (10, 9, 6)		70.7	4.09 m	7.14	70.8	4.10 (o)		70.8	4.08 (o)		70.7	4.09 (o)	
5_ax_	67.3	3.63 dd (11; 10)	-	67.4	3.64 dd (11; 11)	-	67.5	3.66 dd (11; 11)	-	67.4	3.64 dd (11; 11)	-	67.3	3.63 dd (11; 11)	-
5_eq_		4.20 dd (11; 5)			4.20 dd (11; 5)			4.22 dd (11; 6)			4.20 dd (11; 5)			4.20 dd (11; 5)	
		β-d-Api			β-d-Api						β-d-Xyl'			α-l-Rha	
1	111.5	6.08 d (2)	-	111.5	6.08 d (2)	-				106.4	5.07 d (8)	-	102.8	6.08 d (2)	-
2	77.7	4.69 d (2)		77.6	4.69 br s	7.30				75.6	3.90 dd (8, 9)		72.4	4.65 br d (3)	
3	80.5	-		80.5	-					77.7	4.04 dd (9, 9)		72.7	4.45 dd (9, 3)	
4	65.7	4.12 brs (2H)		65.7	4.12 brs (2H)	6.62				70.9	4.09 ddd (10, 9, 5)	7.03	74.2	4.27 dd (9, 9)	
5	75.2	4.24 d (9)	-	75.2	4.24 d (9)	-				67.3	ax3.54 dd (11, 10)	-	69.8	4.88 dq (9, 6)	-
		4.63 d (9)			4.63 d (9)						eq 4.20 dd (11, 5)				
6													18.7	1.62 d (6)	-

a Assignments were confirmed by ^1^H–^1^H-COSY, 2D-TOCSY, HSQC, HSQC-TOCSY, and HMBC experiments.

b o: overlapped with other signals.

#### Coloratoside B (**2**)

[α]_Na_^25^ – 18.5 (*c* 0.20, MeOH); ^1^H and ^13^C NMR, see Tables 2 and 3; HRESIMS (negative ion mode) *m*/*z* 1223.5313 [M + CH_3_COO**^–^**]**^–^** (calcd for C_56_H_87_O_29_, 1223.5333); MS^E^*m*/*z* 1177 [M – H]^−^, 1045 [M – H – 132]^−^, 883 [M – H – 162–132]^−^, 751 [M – H – 162–132 × 2]^−^, 589 [M – H – 132 × 2–162 × 2]^−^

#### Coloratoside C (**3**)

[α]_Na_^25^ – 4.0 (*c* 0.13, MeOH); ^1^H and ^13^C NMR, see Tables 2 and 3; HRESIMS (negative ion mode) *m*/*z* 1091.4918 [M + CH_3_COO**^–^**]**^–^** (calcd for C_51_H_79_O_25_, 1091.4910); MS^E^*m*/*z* 1045 [M – H]^−^, 883 [M – H – 162]^−^, 751 [M – H – 162–132]^−^, 589 [M – H – 132–162 × 2]^−^

#### Coloratoside D (**4**)

[α]_Na_^25^ – 12.1 (*c* 0.05, MeOH); ^1^H and ^13^C NMR, see Tables 2 and 3; HRESIMS (negative ion mode) *m*/*z* 1223.5313 [M + CH_3_COO**^–^**]**^–^** (calcd for C_56_H_87_O_29_, 1223.5333); MS^E^*m*/*z* 1177 [M – H]^−^, 1045 [M – H – 132]^−^, 883 [M – H – 162–132]^−^, 751 [M – H – 162–132 × 2]^−^, 589 [M – H – 132 × 2–162 × 2]^−^

#### Coloratoside E (**5**)

[α]_Na_^25^ – 14.8 (*c* 0.05, MeOH); ^1^H and ^13^C NMR, see Tables 2 and 3; HRESIMS (negative ion mode) *m*/*z* 1237.5476 [M + CH_3_COO**^–^**]**^–^** (calcd for C_57_H_89_O_29_, 1237.5490); MS^E^*m*/*z* 1191 [M – H]^−^, 1045 [M – H – 132]^−^, 883 [M – H – 146–162]^−^, 751 [M – H – 132–146–162]^−^, 589 [M – H – 132–146–162 × 2]^−^

### UPLC-QTOF/MS^E^ Analysis and HMAI Method

The HMAI procedure and the method to obtain the exact masses and fragments of the saponins are described in the Supporting Information.^6^

## Results and Discussion

The most widely used technique to ascertain the contents of saponins in a given extract is UPLC/MS.^11−13^ The molecular ion [M – H]^−^ and the fragmentation patterns provide information on the sequential loss of sugar moieties, and the last and most intense fragment usually corresponds to the aglycone linked to the innermost monosaccharide.^10^

In the bioactive fractions of the four species from the subgenus *Agave* discussed here,^6^ the observed fragments ([aglycone – H + 162]^−^) correspond to spirostanols with additional oxygenations and unsaturations (Table 4). Sugar chains containing four and five sugar units were also identified in these saponin-rich fractions.

**Table 4:** Relative Abundance of the Aglycone Fragments [Aglycone – H + 162] of Saponins Found in Four Species from the *Agave* Subgenus Agave in the UPLC-QTOF/MS^E^ Analysis

aglycone fragments (Da)	*A. colorata*	*A. macroacantha*	*A. parryi*	*A. parrasana*
589 (A3 and/or A5)	10.1%	26%	11.2%	10.3%
591 (A1)	64.0%	39.9%	53.4%	24.6%
593 (A7)				15.0%
605 (A4 and/or A6)		7.2%	3.9%	19.6%
607 (A2)		17.7%	16.1%	20.8%
771 (A8)	2.6%	5.1%	13.6%	3.1%

Although some of the molecular ions and the fragmentations could correspond to saponins that have already been described for the genus *Agave*,^8^ nuclear magnetic resonance was proposed as a complementary technique to determine the structures of the major saponins unambiguously. The ^1^H NMR spectrum of a saponin-rich fraction shows two differentiated regions corresponding to the signals that are most representative of the structure (singlet and doublet signals for the methyl groups of the aglycone backbone in the higher-field region and the anomeric protons of sugar residues in the lower-field region). This enables the dereplication of aglycones and sugar chains separately, and in combination with the information provided by UPLC/MS analysis, the structures of saponins present in saponin-rich fractions can be proposed.

### Dereplication of Aglycones

**Figure 1:**
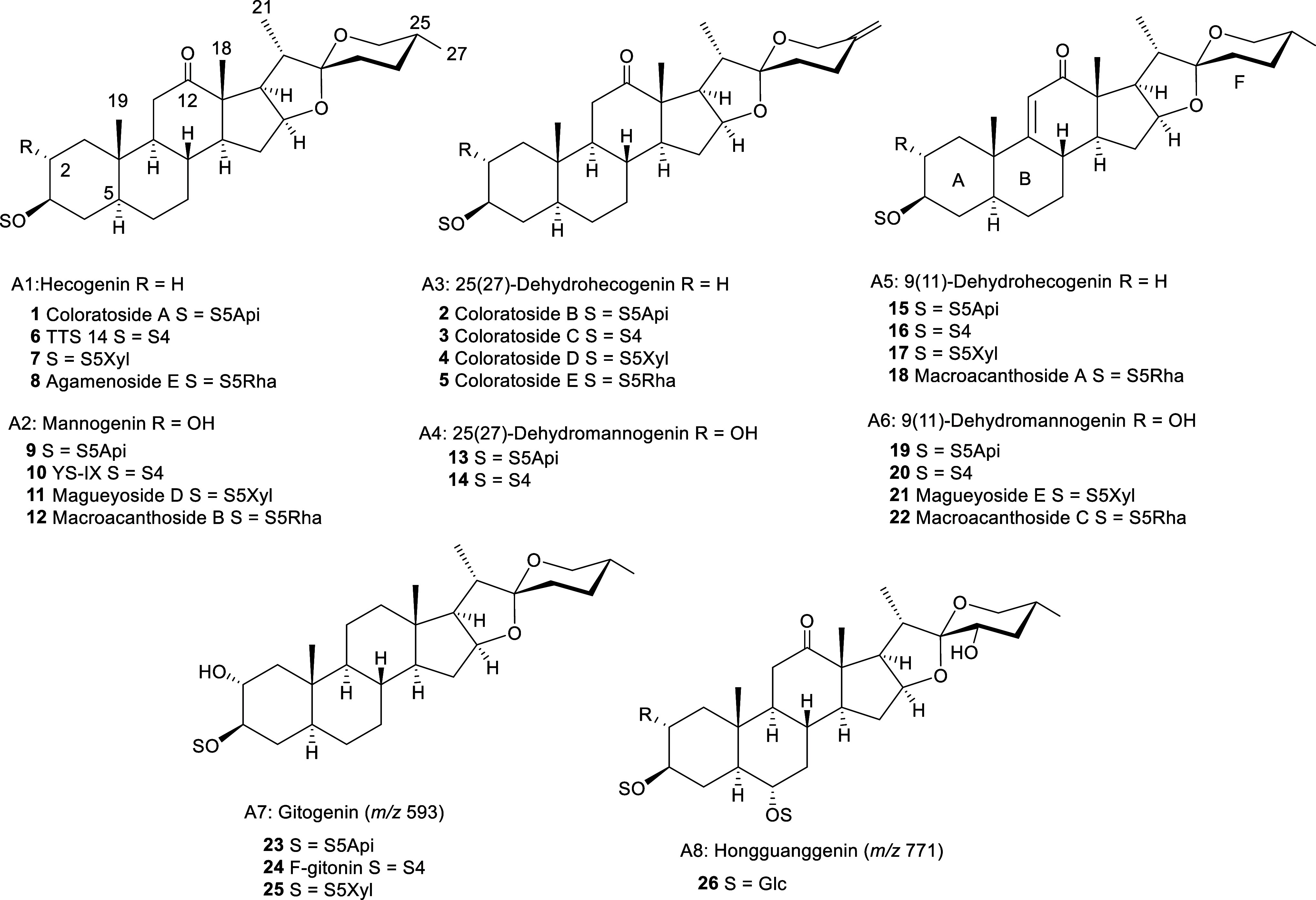
Aglycones and saponins of the four bioactive fractions of *Agave*.

The dereplication strategy employed in this study to identify the aglycones of the main saponins in the extracts involved the use of the HMBC method for aglycone identification (HMAI).^14^ This method was developed using ^13^C and ^1^H NMR data for aglycones from *Agave* saponins reported in the literature. The approach involves the use of ^1^H NMR and HMBC spectra to identify common structural features of aglycones. The presence of chiral centers or functionalization has a strong influence on proton and carbon chemical shifts at positions in close proximity and certain regularities could be observed. Since most of the functionalization found in *Agave* plants is up to four bonds away from methyl groups, the HMBC correlations of these methyl signals are proposed for aglycone identification. The signals of methyl groups are particularly intense in the ^1^H NMR spectra and are easy to recognize in a mixture. As a consequence, the set of HMBC correlations of two groups of signals, namely, singlets (C-18 and C-19) and doublets (C-21 and C-27), are analyzed using two flowcharts (Supporting Information). The information obtained from HMAI enables any structural features to be proposed that have already been described in the literature for *Agave* saponins. As in a previous study on *A. macroacantha*,^10^ the HMAI analysis of the saponin-rich fractions of *A. colorata*, *A. parryi*, and *A. parrasana* identified the structural features of the main aglycones (Table 5). A combination of the relative intensities of the methyl signals in the ^1^H NMR spectrum and the relative abundance of the aglycone fragments in MS^E^ (Table 4) allowed structures to be proposed for the aglycones that form part of the saponins present in the saponin-rich fractions. The aglycones identified were hecogenin (A1), mannogenin (A2), their unsaturated derivatives at C25(27) and C9(11) (A3–A6), gitogenin (A7), and hongguanggenin (A8) (Figure 1).

**Table 5:** Correlations between Methyl Groups and Nearby Carbons Found in the HMBC Spectrum of the SFs of *Agave colorata*, *A. parryi*, and *A.parrasana*; HMAI Interpretation; and Aglycone Identification

^1^H NMR signal	HMBC correlations			methyl assignment	flowchart information	aglycone
*A. colorata*						
*major signals*						
1.33 d (D8)	42.5	54.1 (D6)	109.2 (D5)	C21	SP C12 CO	A1
1.16 d (D10)	35.6	62.4	111.5 (D5)	C21	check data→SP C23 OHα	A8
1.05 s (S2)	54.1	55.5	212.7 (S1)	C18	SP C12 CO	A1
0.94 s	41.0	56.2	62.4 (S11)	C18	check data→C6 OGlc α	A8
0.72 d (D13)	31.6	38.7 (D13)	65.9	C27	SP C23 OHα	A8
0.66 d (D2)	29.0 (D1)	30.4	66.8 (D1)	C27	SP C25 R	A1
0.64–0.62 s (S18)	36.3	44.3	55.4 (S9)	C19	check data →H-5α	A1
0.59 s (S15)	36.8	50.7	53.7 (S13)	C19	C3 OGlcβ; C6 OGlcα^a^	A8
*minor signals*						
1.29 d (D8)	42.3	54.2 (D6)	109.3 (D5)	C21	SP C12 CO	A3
4.76, 4.79 (D14)					SP C25 DB	A3
*A. parryi*						
*major signals*						
1.32 d (D8)	42.4	54.0 (D6)	109.1 (D5)	C21	SP C12 CO	A1
1.31 d (D8)	42.4	54.0 (D6)	109.1 (D5)	C21	SP C12 CO	A2
1.15 d (D10)	35.6	62.3	111.4 (D5)	C21	check data→SP C23 OHα	A8
1.04 s (S2)	54.0	55.4	212.6 (S1)	C18	SP C12 CO	A1
1.03 s (S2)	54.0	55.3	212.4 (S1)	C18	SP C12 CO	A2
0.93 s (S12)	40.9	56.0 (S9)	62.3 (S11)	C18	check data→C6 OGlc α	A8
0.71 d (D13)	31.6	38.9 (D13)	65.8	C27	SP C23 OHα	A8
0.71–69 s (S17)	37.0	44.4	55.1 (S9)	C19	check data→H-5α C2 OHα	A2
0.66 d (D2)	28.9 (D1)	30.3	66.7 (D1)	C27	SP C25 R	A1–A6
0.64–61 s (S18)	36.1	44.1	55.3 (S9)	C19	check data→H-5α	A1
0.58 s	36.9	50.7 (S13)	53.7	C19	C3 OGlcβ C6 OGlcα^a^	A8
*minor signals*						
1.36 d (D8)	42.8	54.5 (D6)	109.2 (D5)	C21	SP C9 DB C12 CO	A5/A6
1.27 d (D8)	42.2	54.1 (D6)	109.3 (D5)	C21	SP C12 CO	A3/A4
0.97 s (S3)	51.1	52.6/54.2	204.2 (S3)	C18	SP C9 DB C12 CO	A5
0.96 s (S3)	51.1	52.4/54.2	204.0 (S3)	C18	SP C9 DB C12 CO	A6
0.86 s (S4)	40.3	42.8	170.3 (S4)	C19	H-5α C2 OHα C9 DB C12 CO	A6
0.78 s (S5)	40.3	42.1	171.2 (S5)	C19	H-5α C9 DB C12 CO	A5
4.75, 4.79 (D14)					SP C25 DB	A3/A4
*A. parrasana*						
*major signals*						
1.38 d (D8)	43.1	54.5 (D6)	109.5 (D5)	C21	SP C9 DB C12 CO	A5/A6
1.33 d (D8)	42.6	54.3 (D6)	109.4 (D5)	C21	SP C12 CO	A1/A2
1.10 d (D10)	42.1	63.0	109.3 (D5)	C21	check data→C21	A7
1.04 s (S2)	55.5	54.4	212.6 (S1)	C18	SP C12 CO	A1/A2
0.97 s (S3)	51.4	52.8/54.5	204.2 (S3)	C18	SP C9 DB C12 CO	A5/A6
0.85 s (S4)	40.6	42.5/43.5	170.5 (S4)	C19	H5α C2 OHα C9 DB C12 CO	A6
0.78 s (S5)	35.1	40.5	171.4 (S5)	C19	H5α C9 DB C12 CO	A4
0.77 s	40.6	56.3	63.0 (S11)	C18	check data→C18	A7
0.69 s	37.3	44.7	55.3 (S11)	C19	check data→C19	A2
0.66 s	36.9	44.8	54.3 (S11)	C19	check data→C19	A7
0.66 d (D2)	29.2 (D1)	30.6	67.0 (D1)	C27	SP C25 R	A1–A6
0.63–61 s	36.5	44.5	55.4 (S9)	C19	check data→H-5α	A1
*minor signals*						
1.29 d (D8)	42.5	54.3 (D6)	109.5 (D5)	C21	SP C12 CO	A3/A4
0.59 s	37.0	50.9 (S13)	53.8	C19	C3 OGlcβ C6 OGlcα	A8
4.76, 4.79 (D14)					SP C25 DB	A3/A4

a With a signal in the ^1^H NMR spectrum at 3.34 ppm; *brd*; 12 Hz (S15). Flowchart information: SP spirostanic structure, DB double bond, CO carbonyl group, OH hydroxyl group, OGlc glucopyranosyloxy group, R, α, β chiral center configuration, C# position in the aglycone, D# and S# decisions in the doublet or singlet flowchart.

The HMAI method enabled the identification of two isomeric aglycones in which the only difference was the position of the double bond. The presence of a double bond at C25(27) has only been described for one saponin from the genus *Agave*,^15^ although it is common in saponins from other genera of the Agavaceae family.^16^

### Dereplication of Sugar Chains

Sugar chains of saponin-rich fractions from *A. macroacantha*, *A. colorata*, *A. parryi*, and *A. parrasana* were selected for joint dereplication since they presented four common fragmentation patterns in the UPLC-QTOF/MS^E^ analysis that are repeated for the major saponins. One of these main saponins is common in all four species, namely, cantalasaponin-1 (**26**), which has previously been described in *A. macroacantha*(10) and is formed by hongguanggenin (A8) as the aglycone with glucose at positions C-3 and C-6. Furthermore, the remaining aglycones are glycosylated at C-3 with sugar chains that are distinguished by three fragmentation patterns, corresponding to sugar chains with four (S4) and five (S5Pent and S5Deox) units (Table 6).

**Table 6:** Relative Percentages of Cantalasaponin-1 (**26**) and Saponins with the Three Fragmentation Patterns Found in the UPLC-QTOF/MS^E^ Analysis

sugar chain	*A. colorata*	*A. macroacantha*	*A. parryi*	*A. parrasana*
S5Deox	15.9%	77.2%	5.5%	-
S5Pent	45.9%	9%	54.7%	36.4%
S4	14.8%	4.5%	25.3%	50.2%
cantalasaponin-1 (**26**)	21.8%	5.1%	12.8%	4.85%

By way of example, the retention times of the saponins derived from hecogenin (A1) and their fragmentation patterns are provided in Table 7. The three fragmentation patterns share identical fragments. Additionally, the loss of a deoxyhexose or a pentose unit is observed in S5Deox and S5Pent. Moreover, duplicate peaks appear with the S5Pent fragmentation patterns, so it is likely that isomeric sugar chains are present.

**Table 7:** Retention Time and Fragmentation of Saponins with Hecogenin (A1) as Aglycone Found in the UPLC-QTOF/MS^E^ Analysis

retention time (min)	[M – H]^−^	fragmentation (*m*/*z*)	sugar chain
3.47–49	1193.5541	1061(−132), 885(−146–162), 753(−132–146–162), 591	S5Deox
3.54–57 and 3.63–65	1179.5421	1047(−132), 885(−162–132), 753(−162–132 × 2), 591	S5Pent
3.69–72	1047.4993	915(−132), 885(−162), 753(−162–132), 591	S4

**Figure 2:**
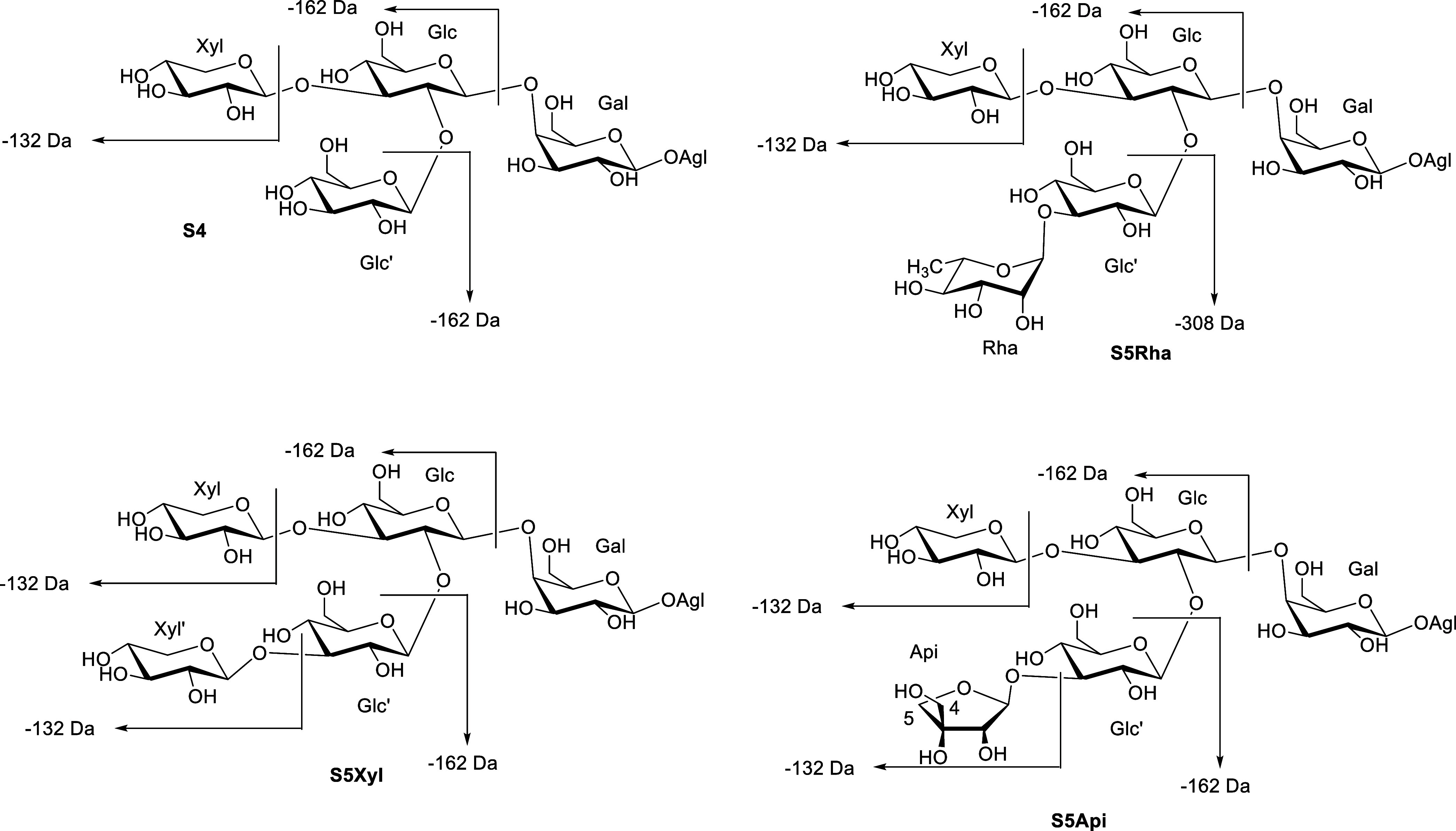
Sugar chains for the saponins of the four bioactive fractions of *Agave*.

The fragmentation patterns observed are consistent with some sugar chains described for spirostanic-type saponins with H-5α (Figure 1) for *Agave* species. The sugar chain S4 is formed by three hexoses and one pentose. According to the fragmentation pattern, one of the hexoses and one pentose are external, which indicates that this is a branched chain consistent with one described previously: β-d-glucopyranosyl-(1→2)-[β-d-xylopyranosyl-(1→3)-β-d-glucopyranosyl]-(1→4)-β-d-galactopyranoside (Figure 2).

Regarding the chain S5Deox, rhamnose is the only deoxyhexose described in *Agave* saponins. If the chain S4 is taken as a starting point, glycosylation of C-3 of glucose (Glc’) or C-4 of xylose has been described. In this case, the fragmentation observed shows a loss of 308 Da, which corresponds to one unit of glucose and rhamnose together, so the second possibility can be ruled out. Thus, the proposed structure for sugar S5Deox is α-l-rhamnopyranosyl-(1→3)-β-d-glucopyranosyl-(1→2)-[β-d-xylopyranosyl-(1→3)-β-d-glucopyranosyl]-(1→4)-β-d-galactopyranoside (S5Rha), which has previously been reported in *A. macroacantha*.^10^ Finally, the only reported chain derived from S4 that is consistent with the fragmentation observed for S5Pent possesses a xylose at position 3 of glucose (Glc’), so it is proposed that at least one of the chains is β-d-xylopyranosyl-(1→3)-β-d-glucopyranosyl-(1→2)-[β-d-xylopyranosyl-(1→3)-β-d-glucopyranosyl]-(1→4)-β-d-galactopyranoside (S5Xyl).

In order to confirm that S4, S5Rha, and S5Xyl (Figure 2) are part of the saponins from the species under investigation, the NMR chemical shifts of these chains described in the literature were analyzed and compared with the experimental data for the fractions in question. It can be considered that the signals from the same sugar chain of different saponins add up to give a single set of signals. However, in previous studies on the bioactive fraction of *A. macroacantha*,^10^ it was observed that the presence of a hydroxy group at the C-2 position of the aglycone had a significant influence on the chemical shifts of the inner sugars of the S5Rha chain. To determine whether the same effect occurs for the other chains, the published ^13^C NMR chemical shifts were compared and similar trends were observed for the galactose unit, but more marked differences were found for the Glc’ unit (Table 8).

**Table 8:** Influence of the Presence of a Hydroxy Group at the C-2 Position of the Aglycone on the Chemical Shifts of the Sugar Chains S4, S5Rha, and S5Xyl^a^

^13^C NMR	S4	S5Rha	S5Xyl
1Gal	+0.9	+0.7	+0.7
2Gal	–0.6	–0.6	–0.6
4Gal	–0.5	–0.8	–0.5
5Gal	+0.4	+0.4	+0.5
1Glu’	–0.3	–0.6	–0.5
3Glu’	–0.5	–0.3	-
4Glu’	+0.3	–0.3	-
5Glu’	+0.7	-	-

a These values are expressed as a difference in the ^13^C NMR chemical shift.

The chemical shift values for galactose^10,17−19^ did not differ significantly for the three chains studied – a finding that is understandable since galactose is glycosylated at C-4 with β-d-glucopyranosyl-(1→2)-β-d-glucopyranoside in all three cases (Figure 2). Thus, two differentiated sets of NMR signals for the galactose unit in these saponin-rich fractions will be observed, and they are related to the presence or absence of a hydroxy group at C-2 of the aglycone backbone regardless of the rest of the sugar chain.

The glucose unit linked at C-4 of the galactose is affected very little by the presence of a hydroxy group at C-2 of the aglycone, but a stronger influence is observed for the next glucose moiety (Glc’). This observation can be explained by chain-folding of the sugar chain, which places this glucose unit at a position closer to the aglycone.^10^ This influence is different (Table 8) for the three sugar chains S4, S5Xyl, and S5Rha because they are differentiated by the substitution at C-3 of the Glc’ unit, which could in turn affect their conformation (Figure 2). The anomeric carbon of the Glc’ unit is shielded in all three cases, but this influence is either insignificant on the remaining carbons of the glucose unit or it is of the opposite sign.

Analysis of the variations in the chemical shifts of ^13^C signals for S4 that generate the substitution of C-3 of the Glc’ unit (Table 9) to lead S5Rha and S5Xyl revealed that the two glucose residues are affected the most. It should be noted that in some cases, the influence is different if the aglycone has a hydroxy group at position C-2. The deshielding of C-3 of the Glc’ is noticeable, and this is around 5 ppm for S5Rha and about 8 ppm for S5Xyl.

**Table 9:** Influence of the Substitution of C-3 of the Glc’ Unit in the Sugar Chain S4

^13^C NMR	Rha	Rha (OH-2)	Xyl	Xyl (OH-2)
1Glc	–0.2	–0.3	–0.2	–0.3
2Glc	–0.2	–0.5	–0.6	–0.6
1Glc’	–0.4	–0.5	–0.8	–0.7
2Glc’	+0.4	–0.5	+0.4	–1.0
3Glu’	+4.5	+5.3	+8.3	+8.9
4Glu’	–1.8	–1.8	–1.9	–1.9
5Glu’	+0.7	–0.5	-	–0.6
6Glu’	–0.8	–0.4	–0.8	–0.4

Overall, the spectrum of a mixture of these three sugar chains will contain the sum of the signals of the galactose, and this is divided into two groups of signals in the case where some aglycones contain a hydroxy group at C-2. The signals of the rhamnose and xylose residues will not be strongly influenced by this substituent, and their intensity will be defined by all of the saponins that contain the corresponding chain. Otherwise, the second glucose (Glc’) moiety will be more influenced by the presence of a hydroxy group at the C-2 position of the aglycone and by the nature of the sugar chain, and as a consequence, greater variability will be observed.

**Figure 3:**
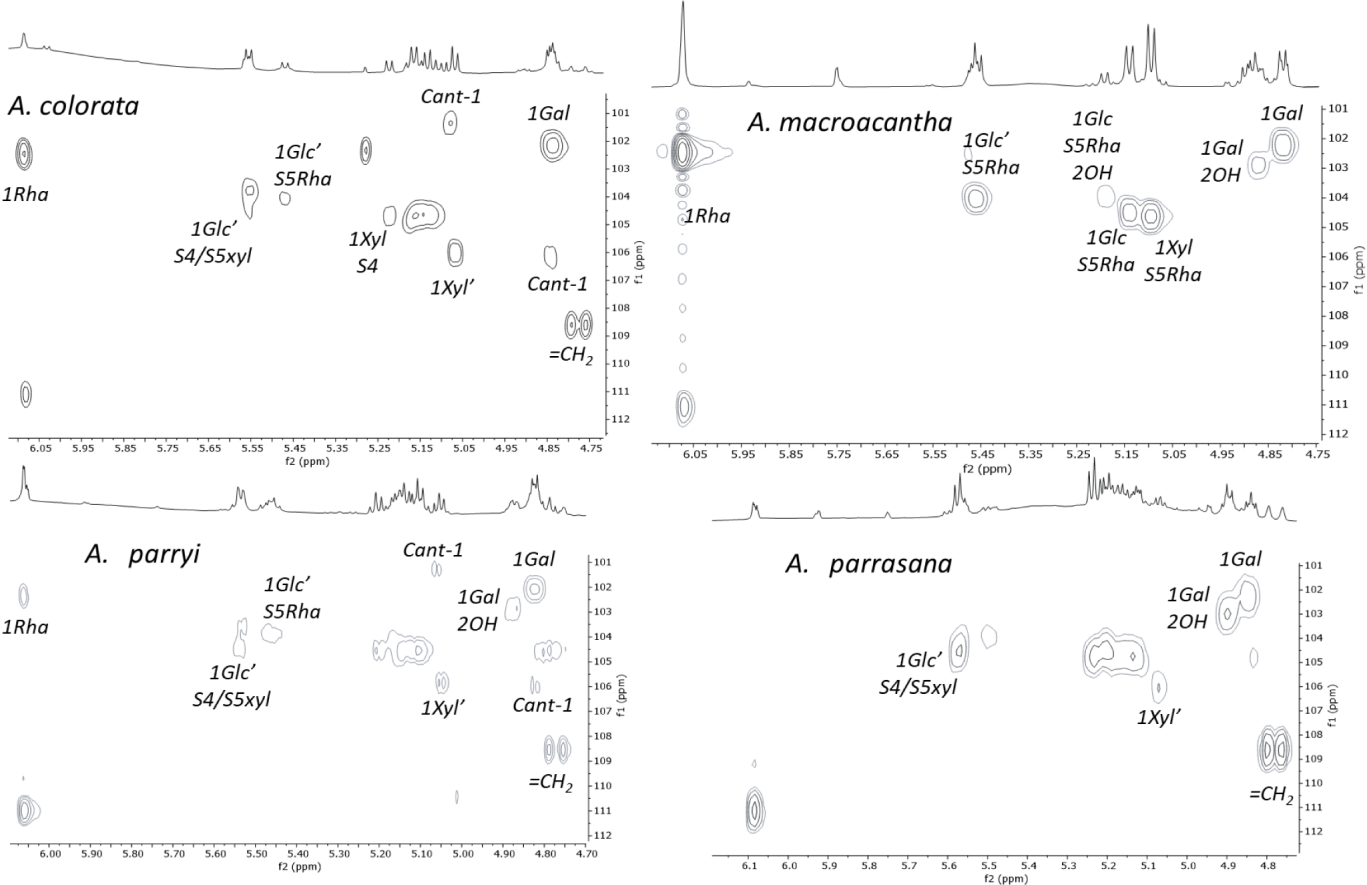
One-bond correlations of the anomeric positions obtained in the HSQC-TOCSY spectrum.

Taking into account the data discussed above, the HSQC-TOCSY and HMBC spectra of the saponin-rich fractions for these four species were analyzed. The HSQC-TOCSY experiment relates the chemical shifts of protons and carbons within the same spin system (Figure 3). First, correlations to one bond from the anomeric positions (C-1 of the monosaccharides) were examined. The chemical shifts reported in the bibliography for the three sugar chains^10,17−19^ are provided in Table 10. Moreover, cantalasaponin-1 (**26**) was found in all of the extracts,^6,20^ and this has two anomeric positions with the correlations 4.85/106.3 ppm and 5.09/101.6 ppm for the two glucose units linked at C-3 and C-6 of the aglycone backbone (Figure 1). Fortunately, these signals are far from the correlations of the sugar chains studied and can be easily distinguished.

**Table 10:** Chemical Shifts of the Anomeric Positions in the ^1^H and ^13^C NMR Spectra of the Sugar Chains S4, S5Xyl, and S5Rha Reported in the Literature

^1^H/^13^C	S4	S4 (OH-2)	S5Xyl	S5Xyl (OH-2)	S5Rha	S5Rha (OH-2)
1Gal	4.86	4.89	4.86	4.90	4.83	4.89
	102.4	103.3	102.6	103.3	102.6	103.2
1Glc	5.18	5.19	5.17	5.19	5.16	5.22
	105.2	104.7	104.9	104.5	104.9	104.3
1Glu’	5.57	5.56	5.59	5.60	5.49	
	104.9		104.0		104.4	
1Xyl	5.22	5.23	5.14	5.15	5.12	
	105.0		104.9		105.0	
1Xyl’			5.10	5.06		
			106.2			
1Rha’					6.10	
					102.8	

The study of these chemical shifts indicated that the anomeric positions of the rhamnose of S5Rha and the second xylose unit of S5Xyl, as well as the galactose residues, are easily distinguishable. Furthermore, the signals of the Glc’ unit of the three sugar chains are found between 5.49 and 5.60 ppm in the ^1^H NMR spectrum, while the signals of the inner glucose and the common xylose unit have similar values.

HSQC-TOCSY spectra (Figure 3) of three saponin-rich fractions showed duplicated correlations for the galactose, as one would expect for the presence of aglycones with and without a hydroxy group at C-2. Hydroxylated aglycones were not found in the saponin-rich fraction of *A. colorata* (Table 4), and consequently, correlations appear for a single set of signals.

The assignment of the rest of the correlations for *A. macroacantha* proved to be simple because its saponins mostly contain one sugar chain (S5Rha). *A. colorata* does not contain hydroxylated aglycones, and its pattern of correlations is therefore less complex, and the signal for 1Xyl from S4 can be distinguished.

The most complex region is between 5.12 and 5.22 ppm in the ^1^H NMR spectrum and between 104.0 and 105.2 ppm in the ^13^C NMR spectrum, where the correlations of the internal glucose (Glc) and xylose (Xyl) of all chains are found.

**Figure 4:**
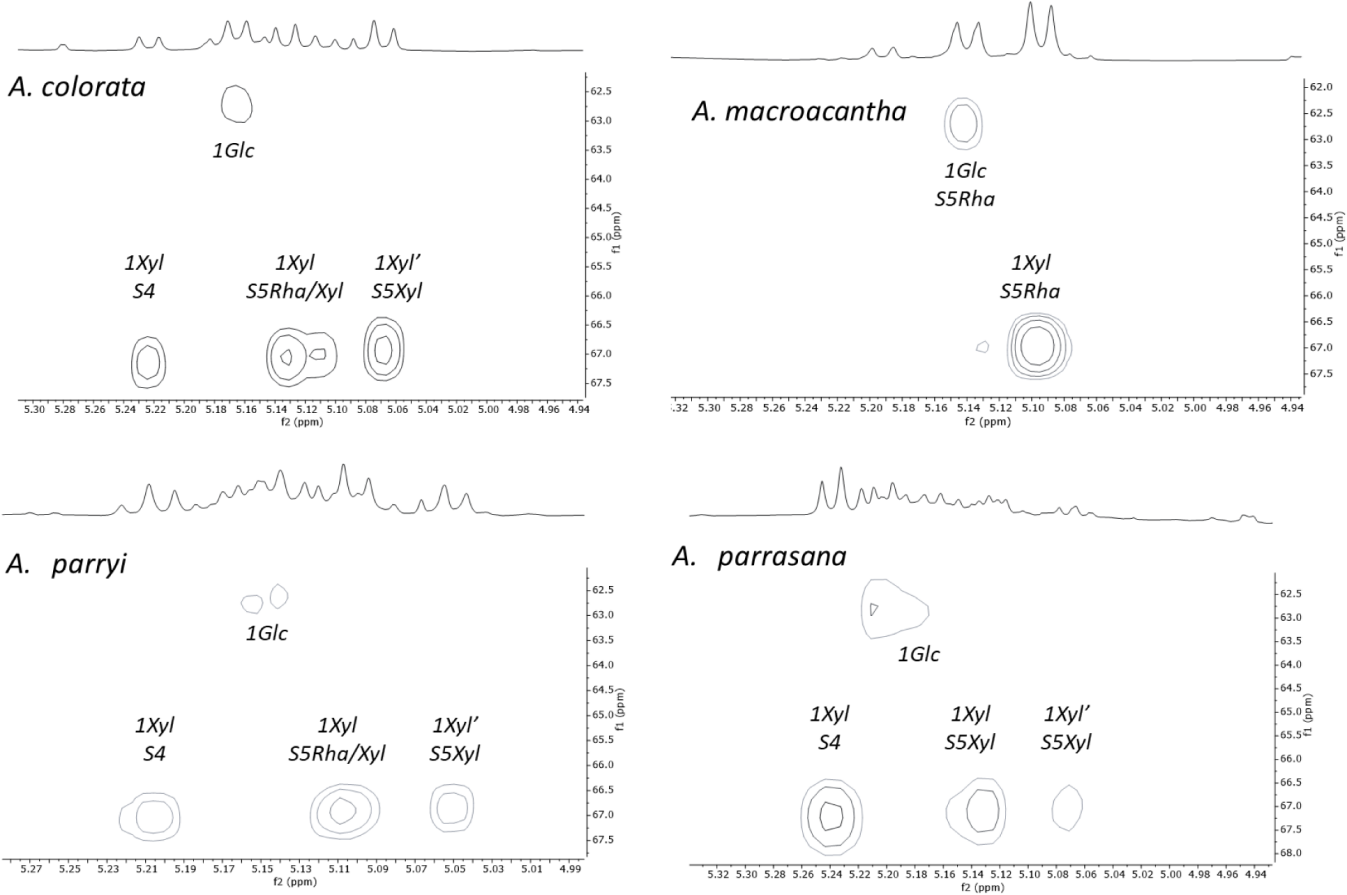
Correlations observed for the methylene groups of glucoses and xyloses with the anomeric protons in the HSQC-TOCSY spectrum.

To complete the identification of anomeric signals of the sugar units, an area of the spectrum corresponding to the methylene groups of the sugar units can be used (Figure 4) since their chemical shifts in the ^13^C NMR spectra are different for each type of monosaccharide. The signal for C-6 of galactose appears at 60.7 ppm, whereas for glucoses, these signals were observed between 62.3 and 63.0 ppm. The methylene signal in the case of xyloses is in the range of 67.1–67.4 ppm. These values are consistent with literature data. The correlation of the anomeric position of the xylose of S4 can be clearly distinguished from that of the glucoses for both *A. parryi* and *A. parrasana*, and in this way, a specific signal can be identified for S4.

**Figure 5:**
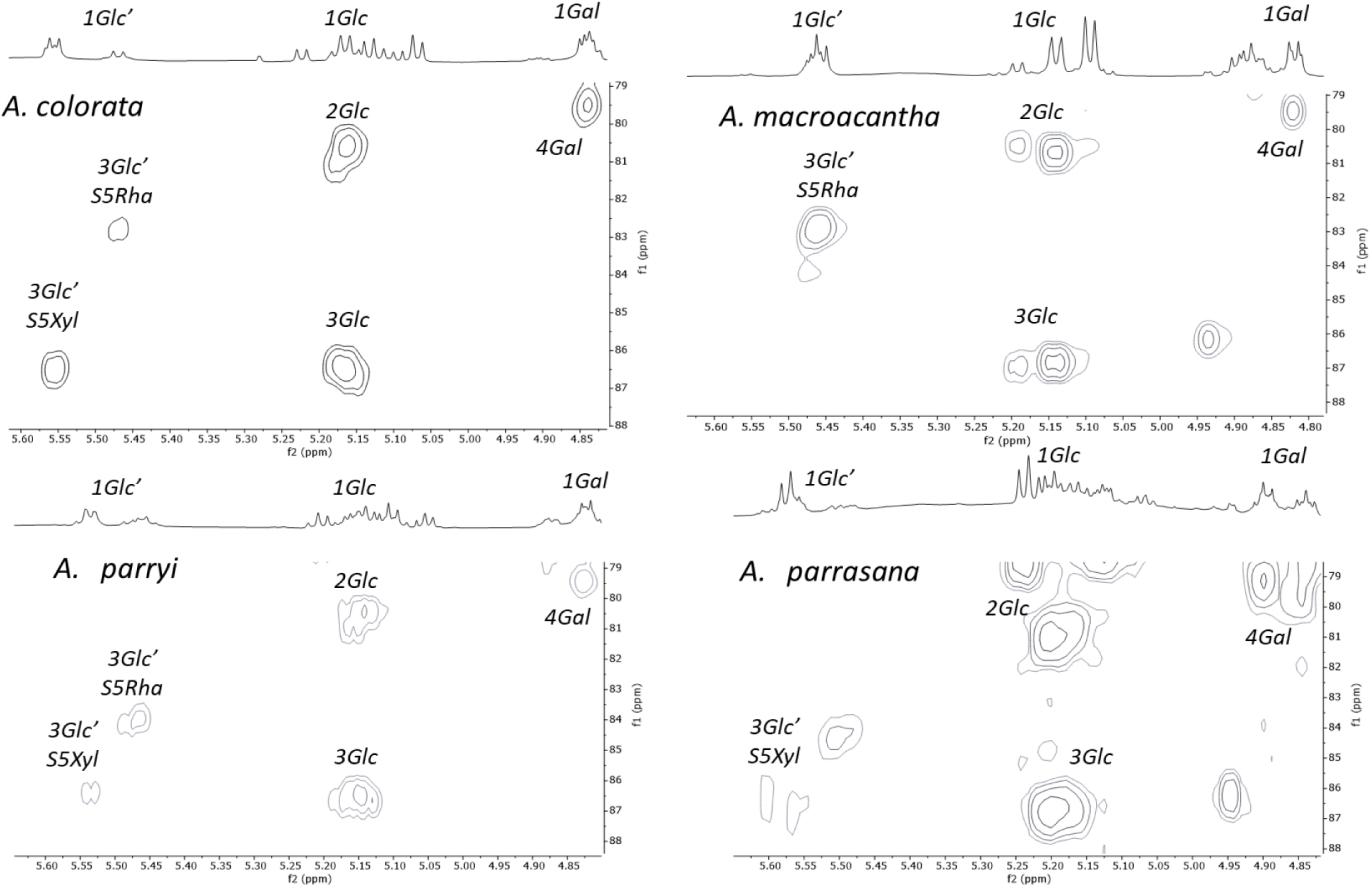
Correlations observed for the glycosylated positions of the glucose units with their anomeric protons in the HSQC-TOCSY spectrum.

The glycosylated carbons of sugar units (Figure 2) are highly deshielded and can be correlated with the anomeric positions in the HSQC-TOCSY spectrum (Figure 5). The correlations observed for the inner glucose unit are usually wide because they appear as the sum of the signals for all sugar chains. Thus, the correlation of 1Glc in the HSQC-TOCSY can appear from 5.16 to 5.22 ppm with 3Glc from 86.8 to 87.3 ppm and with 2Glc from 80.7 to 81.3 ppm.

The chemical shift of 3Glc’ is characteristic for each sugar chain in the ^13^C NMR spectrum. These signals are reported in the literature as being between 78.2 and 78.8 ppm for S4, 86.8 and 87.1 ppm for S5Xyl, and 83.2 and 83.5 ppm for S5Rha. The correlation of these carbons with the anomeric protons of their spin system can be observed in the HSQC-TOCSY spectrum (Figure 5).

These selected regions of the HSQC-TOCSY spectrum enabled the identification of the most significant chemical shifts of each monosaccharide for the three sugar chains, as well as the identification of the anomeric positions in the most congested area. In less congested areas of the spectra, good agreement between the correlations and those described in the literature was observed. In other areas of the spectrum, the correlations are shown as the average of the six possible options (Supporting Information), which arise from three sugar chains and the presence or absence of a hydroxy group at the C-2 position of the aglycone skeleton.

**Figure 6:**
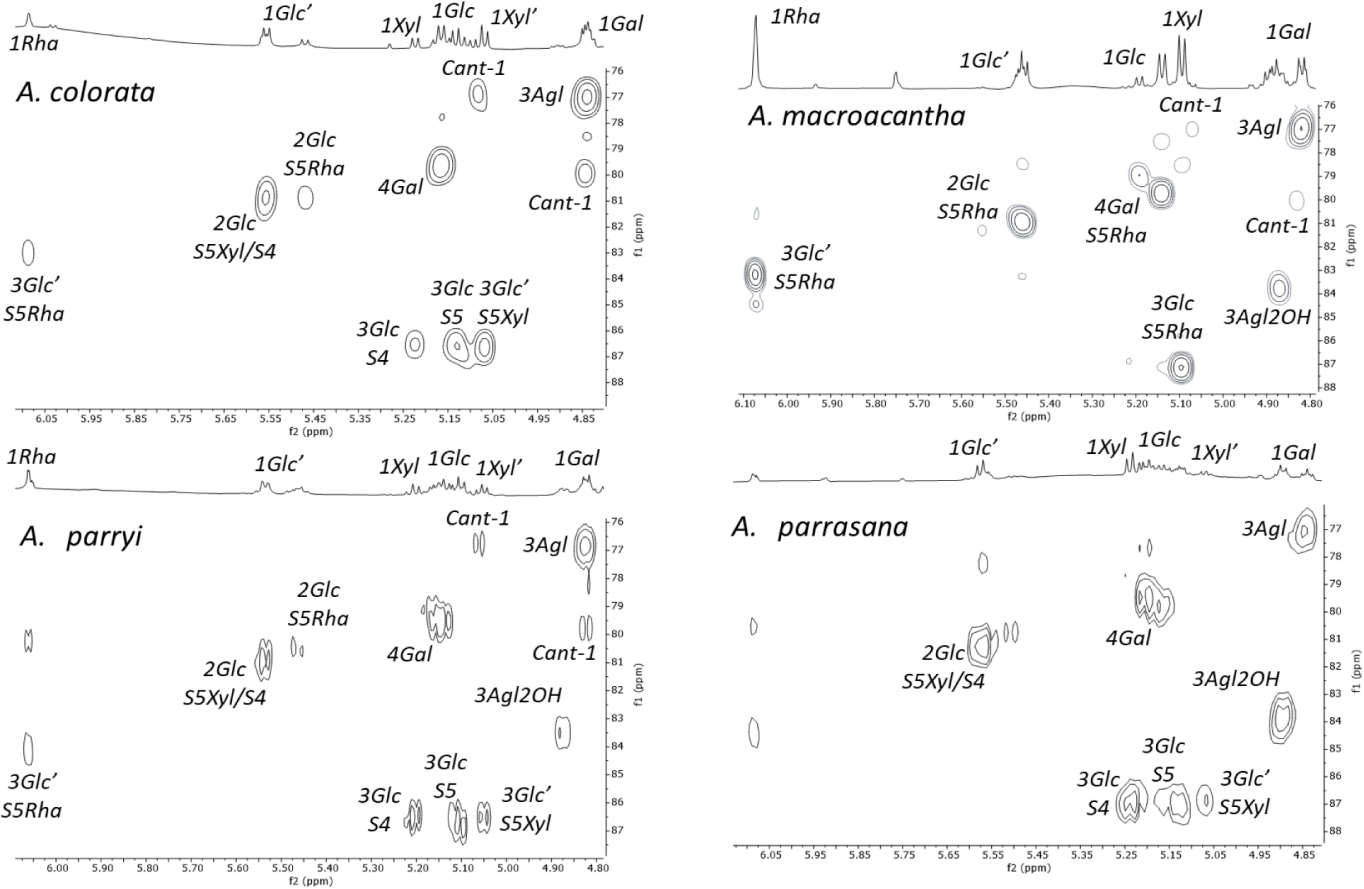
HMBC correlations that define the connections between sugars for S4, S5Rha, and S5Xyl sugar chains and C-3 of aglycone.

HMBC experiments (Figure 6) enable the correlation of protons and carbons up to three bonds away. This approach was employed to confirm the glycosidic bonds in the sugar chain. All of the correlations observed for the saponin-rich fractions are consistent with the literature data. Thereby, the S4, S5Rha, and S5Xyl chains are proposed to be present in the saponins of the saponin-rich fractions under investigation.

**Figure 7:**
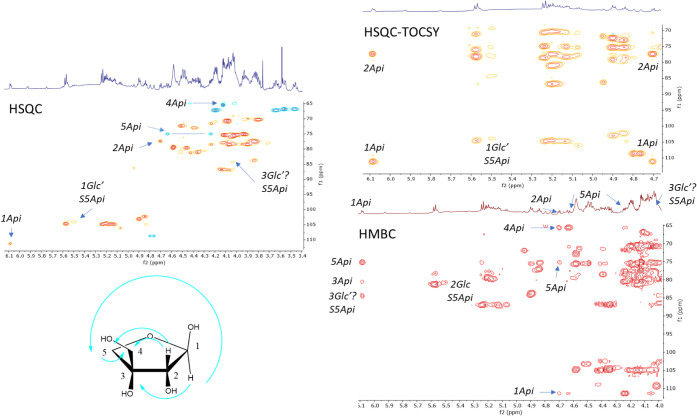
Observed correlations in the bidimensional spectra of *A. parrasana* fraction that justify the presence of an apiofuranose. The blue arrows show the most significant three bond correlations found in the HMBC spectrum.

On considering the bidimensional NMR spectra, an unassigned correlation was observed in the anomeric region for the four extracts (Figure 3) with the same chemical shift as the 1Rha signal in the ^1^H NMR spectrum and at 112.2 ppm in the ^13^C NMR spectrum. The sugar chain S5Rha was not found in *A. parrasana*, as supported by UPLC-QTOF/MS^E^ analysis (Table 6), so this fraction was selected for further study to ascertain the nature of a new monosaccharide using bidimensional HSQC, HSQC-TOCSY, and HMBC spectra (Figure 7). In the HSQC-TOCSY spectrum, the anomeric proton correlated with only one signal in the same spin system, namely, C-2 at 77.4/4.70 ppm. This finding indicates that C-3 is quaternary or that H-3 has a very small coupling constant with C-2. Moreover, two methylene correlations at 65.5/4.12 ppm and 75.1/4.64 and 4.24 ppm were not assigned in the HSQC spectrum of *A. parrasana*, and these could be attributed to a new monosaccharide. The HMBC spectrum shows three correlations at 75.1, 80.5, and 84.4 ppm with the anomeric proton signal, and the first of these places one of the methylene three bonds away from this anomeric proton. Proton H-2 shows correlations with the anomeric position (C-1, 112.2 ppm) and with two methylene groups at 75.1 and 65.5 ppm. In addition, one of the methylene groups correlates with the other through the signals at 4.64 and 4.24 ppm. These correlations confirm that these two methylene groups belong to the same monosaccharide unit (Figure 7) and are consistent with the presence of an apiose unit.^21^ The naturally occurring d-apiose has only been described once in the genus *Agave*,^22^ and it is not common in steroidal saponins.^23−26^ However, when the bioactive fractions were dereplicated, this monosaccharide was found in all cases.

**Figure 8:**
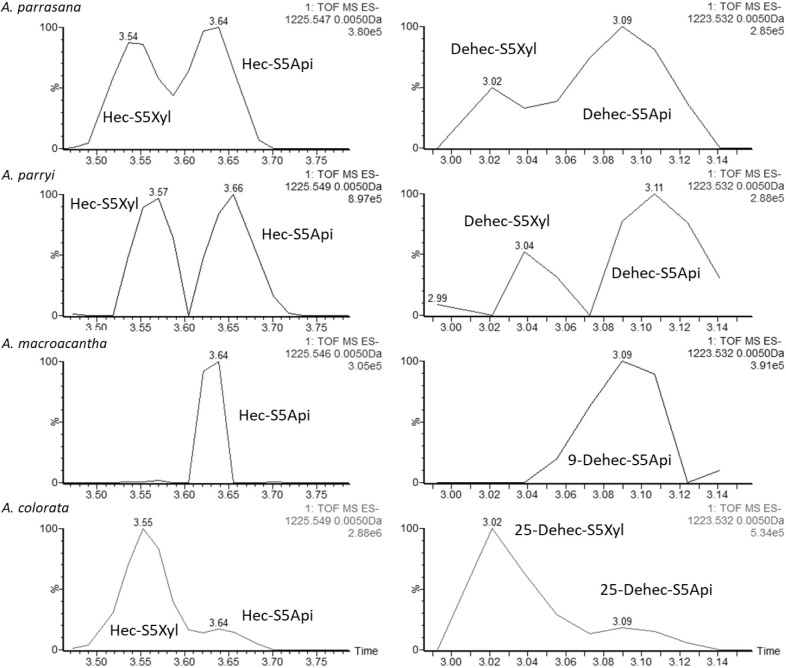
Traces from UPLC-QTOF/MS^E^ analyses for saponins with the molecular ion [M – H]^−^ at 1225 Da (hecogenin-A1 with S5Xyl and S5Api chains) and [M – H]^−^ at 1223 Da (dehydrohecogenin-A3/A5 with S5Xyl and S5Api chains).

Apiofuranose is an isomer of xylopyranose and the loss of a pentose unit (132 Da) would be observed in the UPLC-QTOF/MS^E^ analysis in both cases. This fact could explain the duplicity of peaks observed for some molecular ions (Figure 8). For instance, [M – H]^−^ 1225 and 1223 Da will correspond to saponins with hecogenin (A1) and its dehydroderivative (A3/A5) as aglycones and the sugar chain S5Xyl or S5Api. Moreover, duplicities were not observed for the *A. macroacantha* saponin-rich fraction, and this is consistent with the absence of the sugar chain S5Xyl correlations observed in the HSQC-TOCSY spectrum (Figures 3–6). Comparison of the remaining fractions allowed it to be deduced that, for each pair, the peak with the highest retention time corresponds to saponins with S5Api and the one with the lowest retention time corresponds to that with S5Xyl.

### Isolation of Hidden Saponins

**Figure 9:**
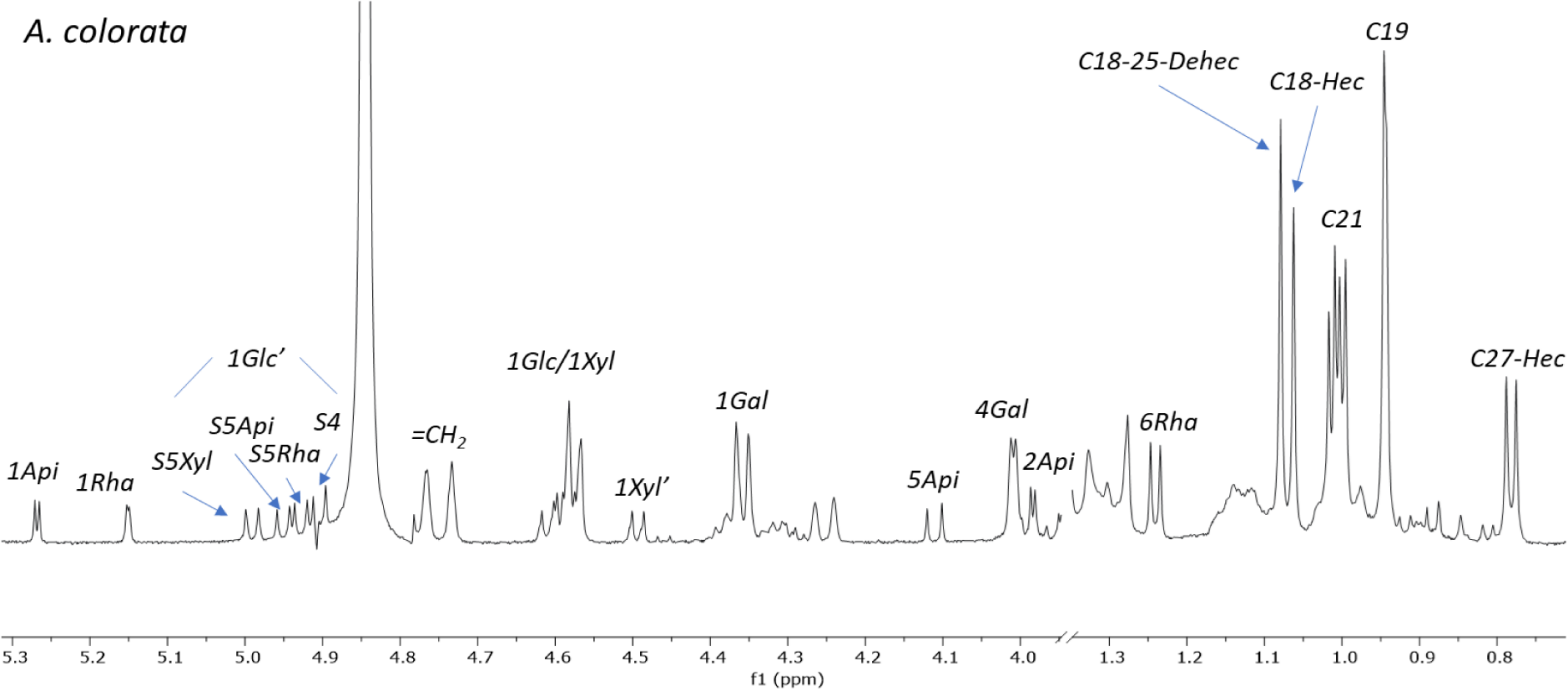
Selected areas of the ^1^H NMR spectrum of the fraction RP18–70% of *A. colorata* dissolved in methanol-*d*_4_ for monitoring of the isolation steps, where the characteristic signals for the four sugar chains and the two types of aglycones are appreciated.

Regarding the position in which apiose would be located in an S5Api chain, it is reasonable to consider that it would be at the C-3 position of Glc’ as in the other S5 chains, and in fact, certain correlations observed in the two-dimensional spectra of *A. parrasana* are consistent with this conclusion (Figure 7). However, this sugar chain has not been reported previously in the bibliography, and spectroscopic data are not available. For this reason, several of the saponins were purified in order to determine unequivocally the nature of the S5Api chain. The *A. colorata* fraction was selected for the purification of saponins with unusual characteristics, including 25(27)-dehydroaglycones. The separation of isomeric saponins that contain double bonds is a very complex task, and this fraction does not contain 9(11)-dehydroderivatives. Fortunately, in a second collection carried out four years later on the same plant, a fraction was obtained with a higher proportion of the saponins of interest. In this case, the purification of saponins with three different sugar chains of five units (S5Xyl, S5Rha, and S5Api) proved to be extremely challenging. The fractions were monitored by ^1^H NMR spectroscopy on samples in methanol-*d*_4_ during the different purification steps. The use of this deuterated solvent enables the anomeric protons of S5Rha and S5Api to be distinguished (Figure 9). Several chromatographic procedures in the normal phase were carried out to separate four pairs of saponins with the different sugar chains, and each pair was subsequently chromatographed in the reverse phase. Monitoring of the separation steps by NMR spectroscopy enabled us to observe a progressive decrease in the proportion of 25(27)-dehydrosaponins – a trend that indicates that some degradation had occurred. Signals consistent with a carbonyl group at C-25 were detected, and this would arise from the oxidation of the C-25(27) double bond.

Eight pure saponins were isolated after a laborious purification process. There were three known saponins derived from hecogenin (A1) and S4, namely, TTS 14^27^ (**6**), S5Xyl^20^ (**7**), and S5Rha, which was named agameroside E^10^ (**8**). Five of these saponins are described for the first time, namely, coloratosides A–E (**1**–**5**, Figure 1).

Coloratosides C–E (**3**–**5**) are the 25(27)-dehydroderivatives of the known saponins. An exhaustive study of the one- and two-dimensional NMR spectra of the pure saponins enabled the description of each of the ^1^H and ^13^C signals of the sugar chain and the aglycone (Tables 2 and 3). A comparison of all the chemical shifts of the known saponins (**6**–**8**) with the new saponins (**3**–**5**) was carried out. The identical chemical shifts of the sugar chains and A/B rings of the aglycone allowed us to establish that the absolute configurations of the monosaccharides are the same as those of the known compounds d-glucose, d-galactose, d-xylose, and l-rhamnose, since the presence of any enantiomeric monosaccharide would generate a diastereoisomer and significant changes in the spectroscopic data of the neighboring units or the aglycone.^28^

Finally, 25(27)-dehydrohecogenin-{β-d-glucopyranosyl-(1→2)-*O*-[β-d-xylopyranosyl-(1→3)-*O*-β-d-glucopyranosyl-(1→4)]-*O*-β-d-galactopyranoside}, 25(27)-dehydrohecogenin-{β-d-xylopyranosyl-(1→3)-*O*-β-d-glucopyranosyl-(1→2)-*O*-[β-d-xylopyranosyl-(1→3)-*O*-β-d-glucopyranosyl-(1→4)]-*O*-β-d-galactopyranoside}, and 25(27)-dehydrohecogenin-{β-d-rhamnopyranosyl-(1→3)-*O*-β-d-glucopyranosyl-(1→2)-*O*-[β-d-xylopyranosyl-(1→3)-*O*-β-d-glucopyranosyl-(1→4)]-*O*-β-d-galactopyranoside} are coloratosides C–E (**3**–**5**), respectively.

A study of the one- and two-dimensional NMR spectra (Tables 2 and 3) of the latter two compounds (**1**, **2**) confirmed the presence of apiose in the sugar chain. The only enantiomer that has been found in plants is d-apiose, and this shares with d-xylose the same metabolic origin from d-glucuronic acid.^29^ As a consequence, it is deduced that this enantiomer is present. The presence of the cyclized form β-d-apiofuranose was established by comparison with the ^13^C NMR signals of the isomeric furanose forms.^30^

A study of the HMBC and NOESY correlations between H-1_Api_ (δ 6.08) and C-3_Glc’_ (δ 84.5)/H-3_Glc’_ (δ 4.03), H-1_Glc’_ (δ 5.49) and C-2_Glc_ (δ 80.8)/H-2_Glc_ (δ 4.34), H-1_Glc_ (δ 5.15) and C-4_Gal_ (δ 79.8)/H-4_Gal_ (δ 4.58), H-1_Xyl_ (δ 5.13) and C-3_Glc_ (δ 87.2)/H-3_Glc_ (δ 4.07), and H-1_Gal_ (δ 4.84) and C-3 (δ 77.2)/H-3 (δ 3.86) of the aglycone enabled the sequence of sugars to be established. Thus, the sugar chain S5Api was determined to be β-d-apiofuranosyl-(1→3)-*O*-β-d-glucopyranosyl-(1→2)-*O*-[β-d-xylopyranosyl-(1→3)-*O*-β-d-glucopyranosyl-(1→4)]-*O*-β-d-galactopyranoside (Figure 2). Finally, coloratosides A (**1**) and B (**2**) are hecogenin-{β-d-apiofuranosyl-(1→3)-*O*-β-d-glucopyranosyl-(1→2)-*O*-[β-d-xylopyranosyl-(1→3)-*O*-β-d-glucopyranosyl-(1→4)]-*O*-β-d-galactopyranoside} and 25(27)-dehydrohecogenin-{β-d-apiofuranosyl-(1→3)-*O*-β-d-glucopyranosyl-(1→2)-*O*-[β-d-xylopyranosyl-(1→3)-*O*-β-d-glucopyranosyl-(1→4)]-*O*-β-d-galactopyranoside}, respectively.

### Dereplication of the Whole Content of Saponin-Rich Fractions

Once it had been confirmed that the combination of UPLC/MS analysis and two-dimensional NMR techniques HSQC-TOCSY and HMBC enabled the elucidation of the structures of the aglycones and the nature of the sugar chains, the identification of all saponins was carried out. In addition to cantalasaponin-1 (**26**), the combination of the seven aglycones with four sugar chains provides a total of 28 saponins, of which 25 were identified with more than 1% relative abundance in the corresponding fraction (Table 11). The identification of some saponins that do not have isomeric alternatives in the sugar chain (S4 and S5Rha) or aglycones (A1, A2, A7) was easily achieved by considering the fragmentations in the UPLC/MS analysis. In this way, the following saponins (Figure 1) could be identified: TTS 14 (**6**),^27^ agameroside E (**8**),^27^ YS-IX (**10**),^31^ macroacanthoside B (**12**),^10^ and F-gitonin (**24**).^32^ In contrast, the sugar chains S5Xyl and S5Api did not give distinguishable fragmentation patterns, although duplicity of the peaks was observed in the chromatogram (Figure 8). The fact that the *A. macroacantha* fraction only contains saponins with S5Api enabled the assignment of each saponin and the identification of those saponins with an S5Xyl chain (Figure 1), which were compound **7**,^20^ magueyoside D (**11**),^19^ and compound **25**.^33^ Saponins with the S5Api sugar chain have not been reported previously. In the current study, it is proposed that this sugar chain is combined with hecogenin-A1 (**1**), mannogenin-A2 (**9**), and gitogenin-A7 (**23**). It is worth noting that in this work, compound **1** was isolated from *A. colorata* and was named coloratoside A.

**Table 11:** Identified Saponins with >1% Relative Abundance in Active Fractions of *Agave colorata*, *A. macroacantha*, *A. parryi*, and *A. parrasana*

					relative abundance in bioactive fractions (%)				
*t*_ret_	[M – H]^−^	aglycone	sugar chain in C-3	compound	*A. colorata*	*A. macroacantha*	*A. parryi*	*A. parrasana*	name or CAS number
1.38	771.4127	A8	glucose	**26**	21.8	5.1	12.8	3.1	cantalasaponin-1
2.09	1207.5341	A6	S5Rha	**22**	-	6.0	-	-	macroacanthoside C
2.13	1193.5232	A6	S5Xyl	**21**	-	-	1.2	-	magueyoside E
2.21	1193.5232	A6	S5Api	**19**	-	1.1	1.2	4.5^a^	-
2.21	1193.5232	A4	S5Api	**13**	-	-	-	4.5^a^	-
2.26	1061.4778	A6	S4	**20**	-	-	1.3	6.4^a^	-
2.26	1061.4778	A4	S4	**14**	-	-	-	6.4^a^	-
2.39	1209.5521	A2	S5Rha	**12**	-	13.9	-	-	macroacanthoside B
2.44	1195.5371	A2	S5Xyl	**11**	-	-	3.0	1.0	magueyoside D
2.52	1195.5371	A2	S5Api	**9**	-	1.6	-	1.2	-
2.58	1063.4946	A2	S4	**10**	-	2.2	7.2	18.6	YS-IX
2.94	1191.5410	A3	S5Rha	**5**	1.9	-	-	-	coloratoside C
2.97	1191.5414	A5	S5Rha	**18**	-	21.4	-	-	macroacanthoside A
3.04	1177.5269	A3	S5Xyl	**4**	6.1	-	-	1.7^a^	coloratoside B
3.04	1177.5269	A5	S5Xyl	**17**	-	-	-	1.7^a^	310883–03–5
3.09	1177.5269	A3	S5Api	**2**	1.0	-	8.1^a^	2.1^a^	coloratoside D
3.09	1177.5269	A5	S5Api	**15**	-	3.5	8.1^a^	2.1^a^	-
3.14	1045.4843	A3	S4	**3**	2.5	-	2.5^a^	3.9^a^	coloratoside A
3.14	1045.4843	A5	S4	**16**	-	1.0	2.5^a^	3.9^a^	168778–20–9
3.49	1193.5599	A1	S5Rha	**8**	14.0	35.8	5.5	-	agamenoside E
3.55	1179.5421	A1	S5Xyl	**7**	32.7	-	15.4	6.1	310883–02–4
3.64	1179.5421	A1	S5Api	**1**	4.2	2.8	17.7	6.9	coloratoside E
3.73	1047.4963	A1	S4	**6**	12.8	1.2	14.2	9.7	TTS 14
5.03	1181.5570	A7	S5Xyl	**25**	-	-	-	2.0	119459–80–2
5.13	1181.5570	A7	S5Api	**23**	-	-	-	2.2	-
5.25	1049.5138	A7	S4	**24**	-	-	-	9.0	*F*-gitonin

a The relative abundance corresponds to the mixture of dehydroderivate saponins with the same sugar chain and the same molecular ion.

The saponins 25(27)- and 9(11)-dehydroderivatives (A3/A5 and A4/A6) coeluted, but the use of the HMAI method for the identification of aglycones indicated that only 9(11)-isomers are found in the bioactive fraction of *A. macroacantha*. In addition to the saponins already identified, macroacanthosides A (**18**) and C (**22**),^10^ which contain the S5Rha chain, we propose the presence of compound **16**,^18^ which contains the S4 chain, and saponins in which the S5Api chain is combined with the aglycones 9(11)-dehydrohecogenin-A5 and 9(11)-dehydromannogenin-A6 (**15** and **19**, respectively) (Figure 1). In contrast, in the bioactive fraction of *A. colorata*, only 25(27)-dehydrohecogenin-A3 was found and the saponins that combine it to each sugar chain were isolated and named coloratosides B–E (**2**–**5**) (Figure 1).

**Figure 10:**
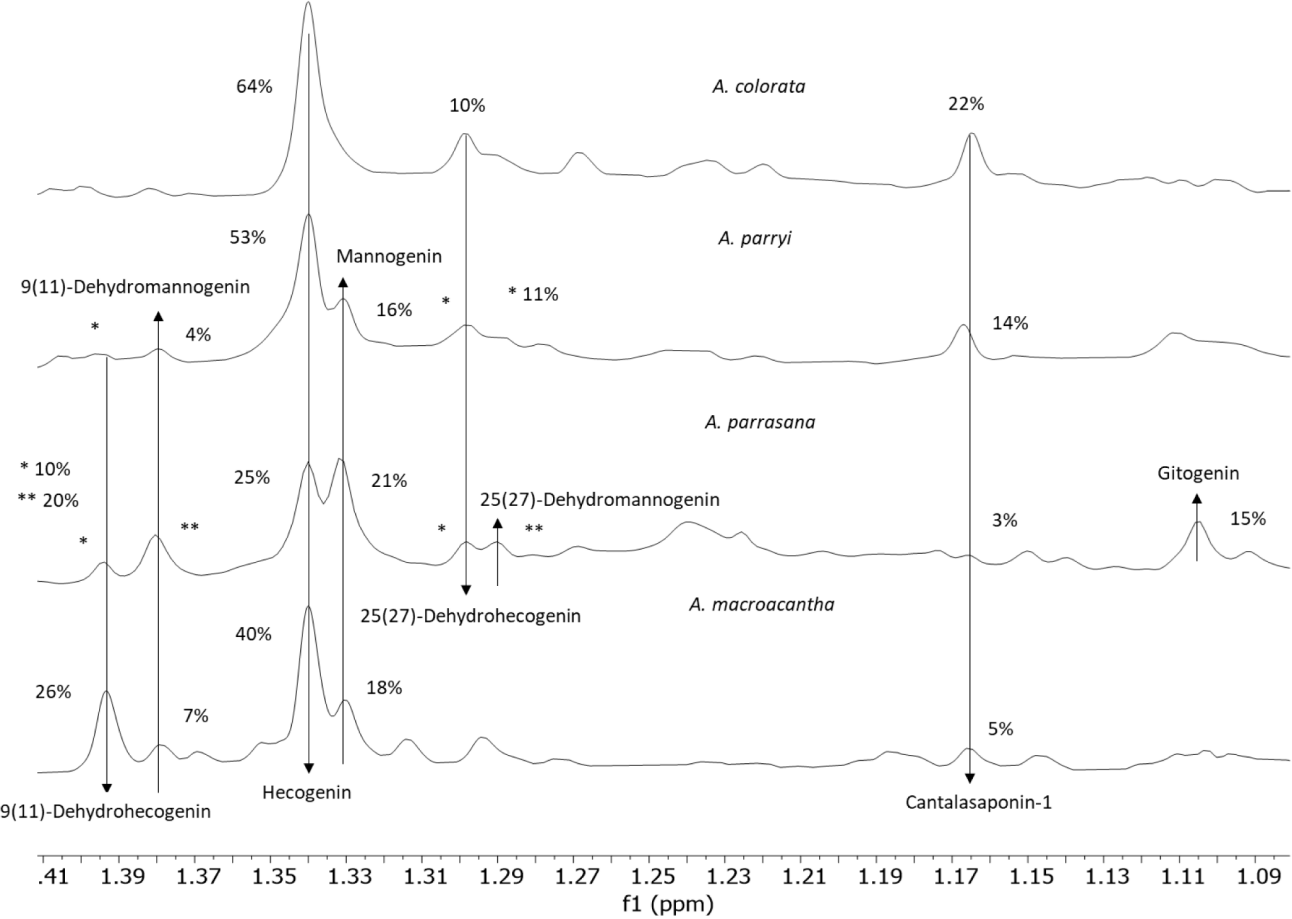
Selected region of the pure shift 1D spectra of the four bioactive fractions analyzed, indicating the signals corresponding to the C-21 methyl groups of cantalasaponin-1 (**26**) and for the other aglycones identified, as well as their relative percentage found in the UPLC/MS.

Finally, the fractions from *A. parrasana* and *A. parryi* are more complex and contain saponins with the two possible unsaturated positions. On applying the HMAI method, it was observed that the signal of methyl C-21 in the ^1^H NMR spectrum is representative of each of the possible aglycones (Table 5) since it is sensitive to the presence of a double bond at C9(11) and C25(27) as well as to the presence of a hydroxy group at C-2.^14^ Pure shift 1D spectra of the four bioactive fractions were therefore evaluated (Figure 10). This experiment avoids proton coupling and enables the C-21 methyl doublet signals to be observed as singlets.

The intensities of the signals for the C-21 methyl group of 9(11)-dehydrohecogenin-A5 (1.39 ppm), 9(11)-dehydromannogenin-A6 (1.38 ppm), hecogenin-A1 (1.34 ppm), mannogenin-A2 (1.33 ppm), 25(27)-dehydrohecogenin-A3 (1.30 ppm), 25(27)-dehydromannogenin-A4 (1.29 ppm), and gitogenin-A7 (1.10 ppm) are consistent with the relative intensities of species found by UPLC/MS. It is believed that the peaks observed in the UPLC/MS chromatogram that correspond to dehydroderivatives contained the aglycones described for each fraction. Thus, magueyoside E (**21**)^34^ and compound **17**(35) were identified. Saponins in which the sugar chain S4 is combined with 9(11)-dehydromannogenin-A6 (**20**) and 25(27)-dehydromannogenin-A4 (**14**), as well as the chain S5Api with 25(27)-dehydromannogenin-A4 (**13**), are proposed.

Finally, application of the dereplication method to the four bioactive fractions allowed the identification of a total of 26 saponins (Table 11); 9 in *A. colorata*, 12 in *A. macroacantha*, 14 in *A. parryi*, and 20 in *A. parrasana*. This group of 26 saponins encompasses a large number of isomers, although only 15 different molecular ions were observed in the UPLC-QTOF/MS^E^ analysis. Futhermore, some of these saponins exhibited identical fragmentation patterns.

Furthermore, besides cantalaponin-1 (**26**), which was structurally different, the other saponins arise from a combination of seven aglycones and four sugar chains that are linked by an *O*-glycosidic bond to C-3 of the aglycone. Thus, the challenge was reduced to the identification of seven aglycones by the HMAI method^6,14^ and four sugar chains.

It should be highlighted that certain saponins have remained hidden as far as traditional phytochemistry is concerned because they are minor components that are difficult to separate chromatographically as they undergo a degradation process in the purification steps – such as the case of 25(27)-dehydroderivatives – or they present overlapped signals or unusual chemical shifts in comparison to more common monosaccharides, as is the case for apiose. Nevertheless, the application of the dereplication strategy to the saponin-rich fractions led to the identification of these minor saponins in all cases. For instance, coloratoside A (**1**), with S5Api as the sugar chain, was found to be present at levels of 4.2% in *A. colorata*, 2.8% in *A. macroacantha*, 17.7% in *A. parryi*, and 6.9% in *A. parrasana*.

The simultaneous dereplication of the fractions makes it easier to identify isomeric saponins with the same characteristics in the UPLC/MS analysis, and in some cases, up to four isomeric saponins were identified (**2**, **4**, **15**, **17**). Dereplication of the fraction from *A. macroacantha* in a previous study^10^ led to the identification of five main compounds. On carrying out this simultaneous dereplication on the other fractions, it was possible to identify seven minor saponins.

The dereplication strategy reported here is not based on a comparison with standards or with UPLC-MS or NMR databases^9^ because this information is not fully available. However, the combined interpretation of the UPLC/MS analysis, NMR spectra, and bibliographic data for saponins of the same genus enabled the identification of 14 saponins that have already been described and the proposal of 12 new structures. The isolation of eight saponins, including examples with uncommon structural features such as a double bond at 25(27) or an apiose unit, confirmed their previous identification and demonstrates the efficacy of the dereplication method proposed here. This dereplication strategy is a powerful tool for unequivocal identification of saponins found in *Agave* extracts. The noteworthy phytotoxicity values and selectivity shown on weeds versus tomato or cress make these saponin-rich fractions attractive candidates for use as bioherbicides.

## Supporting Information Available

Certificate of plant origin; procedures for UPLC-MS and HMAI methods, HMAI flowcharts and tables; selected areas of the HMBC to HMAI method; tables with expected and experimental correlations of HSQC-TOCSY and HMBC of sugar chains in saponin-rich fractions; 2D-NMR spectra of saponin-rich fractions; ^1^H and ^13^C NMR spectra of new compounds **1**–**5**; 1D TOCSY spectra of coloratoside B (**2**) as an example (PDF)
