# Supplementary material for: Dereplication of Bioactive Agave Saponin Fractions: The Hidden Saponins
Source: J Agric Food Chem. 2024 Jun 5;72(24):13740–56. doi: 10.1021/acs.jafc.4c02308 (PMC11191682; doi:10.1021/acs.jafc.4c02308)
Supplement: Supplementary file 1 — jf4c02308_si_001.pdf [file jf4c02308_si_001.pdf]

Supporting information

DEREPLICATION OF BIOACTIVE *AGAVE* SAPONIN FRACTIONS. THE HIDDEN SAPONINS.

Ana M. Simonet*, Alexandra G. Durán, Francisco A. Macías

[1]*Allelopathy Group, Department of Organic Chemistry, Institute of Biomolecules (INBIO), Campus de Excelencia Internacional (ceiA3), School of Science, University of Cadiz, C/ República Saharaui, 7, 11510 Puerto Real, Cadiz, Spain.*

[*]**Corresponding author**: Ana M. Simonet – Associate Professor of Organic Chemistry;

*Email: ana.simonet@uca.es

Table of Contents

Certificate of plants origin from Desert City company 1

Procedure of UPLC-MS analysis and HMAI method 2

Table S1. HMAI Table for doublets 3

Table S2. HMAI Table for singlets 4

Table S3. Expected and experimental correlations of HSQC-TOCSY of sugar chains (S4, S5Xyl, S5Rha, and S5Api) of *Agave* saponin-rich fractions 5

Table S4. Expected and experimental correlations of HMBC of sugar chains (S4, S5Xyl, S5Rha, and S5Api) of *Agave* saponin-rich fractions 6

Figure S1. HMAI Flowchart of doublets 7

Figure S2. HMAI Flowchart of singlets 8

Figure S3. HMBC of saponin-enriched fraction of *Agave colorata.* Selected area of methyl group signals of aglycone. (600 MHz, Pyridine-*d5*). 9

Figure S4. HMBC of saponin-enriched fraction of *Agave macroacantha.* Selected area of methyl group signals of aglycone. (600 MHz, Pyridine-*d5*). 10

Figure S5. HMBC of saponin-enriched fraction of *Agave parryi.* Selected area of methyl group signals of aglycone. (600 MHz, Pyridine-*d5*). 11

Figure S6. HMBC of saponin-enriched fraction of *Agave parrasana.* Selected area of methyl group signals of aglycone. (600 MHz, Pyridine-*d5*). 12

Figure S7. HSQC-TOCSY of saponin-enriched fraction of *Agave colorata.* Selected area of sugar chain signals. (600 MHz, Pyridine-*d5*). 13

Figure S8. HMBC of saponin-enriched fraction of *Agave colorata.* Selected area of sugar chain signals. (600 MHz, Pyridine-*d5*). 14

Figure S9. HSQC-TOCSY of saponin-enriched fraction of *Agave macroacantha.* Selected area of sugar chain signals. (600 MHz, Pyridine-*d5*). 15

Figure S10. HMBC of saponin-enriched fraction of *Agave macroacantha.* Selected area of sugar chain signals. (600 MHz, Pyridine-*d5*). 16

Figure S11. HSQC-TOCSY of saponin-enriched fraction of *Agave parryi.* Selected area of sugar chain signals. (600 MHz, Pyridine-*d5*). 17

Figure S12. HMBC of saponin-enriched fraction of *Agave parryi.* Selected area of sugar chain signals. (600 MHz, Pyridine-*d5*). 18

Figure S13. HSQC-TOCSY of saponin-enriched fraction of *Agave parrasana.* Selected area of sugar chain signals. (600 MHz, Pyridine-*d5*). 19

Figure S14. HMBC of saponin-enriched fraction of *Agave parrasana.* Selected area of sugar chain signals. (600 MHz, Pyridine-*d5*). 20

Figure S15. HSQC of saponin-enriched fraction of *Agave parrasana.* Selected area of sugar chain signals. (600 MHz, Pyridine-*d5*) 21

Figure S16. HRESI MS^E (negative mode) of Coloratoside A (1) 22

Figure S17. ^{1}H NMR spectrum of Coloratoside A (1) (700 MHz, Pyridine-*d5*) 23

Figure S18. ^{13}C NMR spectrum of Coloratoside A (1) (700 MHz, Pyridine-*d_5*) **24**

Figure S19. HRESI MS^E (negative mode) of Coloratoside B (2) **25**

Figure S20. ^1H NMR spectrum of Coloratoside B (2) (700 MHz, Pyridine-*d_5*) **26**

Figure S21. ^{13}C NMR spectrum of Coloratoside B (2) (700 MHz, Pyridine-*d_5*) **27**

Figure S22. HRESI MS^E (negative mode) of Coloratoside C (3) **28**

Figure S23. ^1H NMR spectrum of Coloratoside C (3) (700 MHz, Pyridine-*d_5*) **29**

Figure S24. ^{13}C NMR spectrum of Coloratoside C (3) (700 MHz, Pyridine-*d_5*) **30**

Figure S25. HRESI MS^E (negative mode) of Coloratoside D (4) **31**

Figure S26. ^1H NMR spectrum of Coloratoside D (4) (700 MHz, Pyridine-*d_5*) **32**

Figure S27. ^{13}C NMR spectrum of Coloratoside D (4) (700 MHz, Pyridine-*d_5*) **33**

Figure S28. HRESI MS^E (negative mode) of Coloratoside E (5) **34**

Figure S29. ^1H NMR spectrum of Coloratoside E (5) (700 MHz, Pyridine-*d_5*) **35**

Figure S30. ^{13}C NMR spectrum of Coloratoside E (5) (700 MHz, Pyridine-*d_5*) **36**

Figure S31. Example of ^1H NMR spectra comparison: Coloratoside D (4) and previous fraction (60% of 7 and 40% of 4) with the same structure of sugar chain for both compounds (700 MHz, Pyridine-*d_5*) **37**

Figure S32. Example of 1D TOCSY spectra (120 ms): Sugar chain of Coloratoside B (2). (700 MHz, Pyridine-*d_5*) **38**

Figure S33. Example of 1D TOCSY spectra (120 ms): Aglycone of Coloratoside B (2). (700 MHz, Pyridine-*d_5*) **39**

Certificate of plants origin from Desert City company.

Desert CITY

San Sebastián de los Reyes, October 27th, 2021

CERTIFICATION

This document certifies that the experimental biological material of *Agave* species below described proceed from cultivated specimens in the registered xerophytic plants nursery and botanical garden Desert CITY.

Subgenera	Specie	Fresh weight
Agave	*Agave americana*	110.1
	Agave parrasana	143.2
	Agave macroacantha	32.8
	Agave colorata	117.2
	Agave parryi	124.7
	Agave weberi	85.1
	Agave cupreata	182.8
Littaea	*Agave geminiflora*	60.2
	Agave lophantha	52.8
	Agave nigra	81.7
	Agave xylonacantha	41.1
	Agave bracteosa	125.0
	Agave victoria-reginae	281.1
	Agave fernandi regis	49.8
	Agave celsii	38.6
	Agave triangularis	71.4
	Agave stricta	41.3

Desert CITY is an authorized professional operator for growing and commercialize plants according to the European Plant Health Regulation 2016/2031, with the number of ROPVEG ES-13-28-0490 by the Spanish Ministry of Agriculture.

The biological material was delivered to the Organic Chemical Department of University of Cadiz on November 27th , 2017.

Desert CITY
Desert City S.L • Autovía A1, Km 25 • 28708
San Sebastián de los Reyes, Madrid • CIF: B866914[illegible]

Mercedes García Bravo
Founder of Desert CITY
Pharmacist and Agronomic Engineer.
Nº of Professional membership 5155
Colegio Oficial de Ingenieros Agrónomos de Madrid, Centro y Canarias (COIAMCC)

Desert CITY, S.L.
Biotechnological Xerophytic Plant Nursery
Autovía A1 km 25 San Sebastián de los Reyes 28708 Madrid SPAIN
CIF: B- 86691474

Procedure of UPLC-MS analysis.

A UPLC-QTOF ESI (Waters Xevo G2, Manchester, UK) high-resolution mass spectrometer (HRESI-TOFMS) was used to measure the accurate mass and analysis of saponins of the different saponin-rich fractions. An analysis procedure was performed using the methodology described in previous work (Durán et al., Agronomy, 2021: 11, 2404). Sample solutions (5 µL) were injected into an Acquity UPLC HSS T3 1.8 µm, 2.1 × 5 mm VanGuard precolumn attached to an Acquity UPLC HSS T3 1.8 µm, 2.1 × 100 mm column, maintained at 45 °C. The mobile phase consisted of water (A) and acetonitrile (B), each containing 0.1% (*v*/*v*) formic acid, with the following gradient: 0−0.5 min, 60% A; 0.5−6.0 min, 60%−50% A; 6.0−7.0 min, 50%−95% A; 7.0−7.5 min, 95% A; 7.5−8.0 min, 95%−60% A, and maintenance in 60% A (8.0−10.0 min) to condition the column for the next injection. The flow rate was established at 0.4 mL/min. The temperature in the autosampler was set at 10 °C.

Electrospray Ionization in the negative polarity mode (ESI^-) was used with the following settings: sample probe capillary voltage 2800 V, sampling cone voltage 30 V, source temperature 120 °C and desolvation temperature 450 °C. Desolvation and cone gas with flow rates of 850 and 10 L/h were used, respectively. The data were acquired in the centroid mode using MS^E (low collision energy, 6 eV; high collision energy ramp, 20−80 eV) over a mass range of *m*/*z* 100−2000 and a retention time range of 0–10.0 min with a 0.5 s scan time. Data acquisition and processing were carried out with MassLynx version 4.1 (Waters Inc. Milford, MA, USA, 2013). The stock solutions (1000 ppm) of the saponin-rich fractions were prepared in water:acetonitrile (6:4). All the samples were injected as a dilution 1:15 (66.7 ppm) and filtered through a PTFE syringe filter (0.22 µm) prior to analysis.

Procedure of HMAI method.

Two flowcharts (below) are tools for the identification of aglycones of saponins from the *Agave* species by ^{1}H-NMR and HMBC experiments. The decisions (inside diamonds) are named with D or S and an integer number, and they use both ranges of chemical shifts and absolute values of HMBC correlations in the flowchart. In this last case, values within the error range ±0.04 ppm and ±0.4 ppm for ^{1}H- and ^{13}C-NMR signals should be considered. Spectra should be referenced to deuterated pyridine (7.55 ppm and 135.6 ppm for ^{1}H- and ^{13}C-NMR, respectively).

Prior assignment is not needed and only proton signals (for three equivalent protons) should be distinguished between 1.6 ppm and 0.5 ppm. These signals are readily recognisable and correspond to secondary methyl groups at C-21 and C-27, which are doublets and singlets for angular methyl groups C-18 and C-19. The flowchart should be started with the methyl doublets that provide information on rings C–F. The doublet that is more shielded would be analysed first (usually C-27).

Secondly, methyl groups that give rise to singlets should be investigated and the most deshielded position will be applied in the flowchart to assign each methyl group.

In some cases, the flowchart indicates that HMBC values for a specific methyl should be revised. In this situation, taking into consideration the structural features of the remaining methyls, HMAI Tables (below) should be used.

In a case where the HMBC signals are different to those indicated in the tables, the saponin should have other structural characteristics that will require elucidation.

Table S1. HMAI table for doublets. v 2024. Reprinted with permission from Simonet *et al.*, *Phytochem. Anal.*, 2021: *32*, 38-61 (DOI: 10.1002/pca.2946). Copyright 2021 John Wiley & Sons. Order Number: 5776410703091.

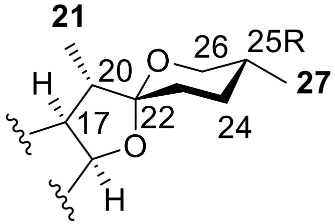

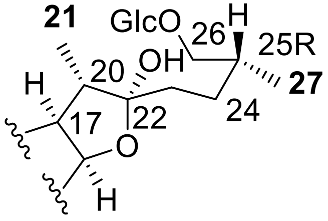

Structural Features						HMBC signals							
C-9	C-12	C-23	C-24	C-22	C-25	**H-27**	C-24	C-25	C-26	**H-21**	C-17	C-20	C-22
				SP	R	0.67	29.1	30.6	66.7	1.12	62.9	41.9	109.3
				SP	DB	-	-	-	-	1.08	63.2	41.9	109.4
				F	R	0.96	28.5	34.4	75.3	1.30	63.9	40.8	110.7
				FM	R	0.98	28.2	34.2	75.2	1.16	64.1	40.5	112.7
		OHα		SP	R	0.72	38.9	31.8	66.0	1.16	62.6	35.9	111.7
			OGlc*β*	SP	R*	1.12	81.5	38.2	65.1	1.02	62.3	42.1	111.6
		OHα	OGlc*β*	SP	R*	1.19	87.9	37.9	64.1	1.15	62.0	34.6	112.7
	CO			SP	S	1.05	26.2	27.5	65.2	1.35	54.2	43.1	109.8
	CO			SP	R	0.64	29.2	30.5	66.9	1.31	54.3	42.6	109.3
	CO			F	R	0.96	28.4	34.3	75.3	1.53	54.9	41.3	110.9
	CO			F	S	1.01	28.3	34.4	75.3	1.51	54.8	41.3	110.8
DB	CO			SP	R	0.67	29.2	30.5	67.0	1.38	54.5	43.0	109.5
	OH*β*			SP	R	0.67	29.4	30.7	66.9	1.41	63.0	43.1	109.6

OH: hydroxyl; DB: double bond; CO: carbonyl; SP: spirostanic; F: furostanic; R/S/*α*/*β*: chiral center configuration.

* R is the relative configuration; S is the absolute configuration because a glucopyranosyloxy moiety is at C-24.

Table S2. HMAI table for singlets. Reprinted with permission from Simonet *et al.*, *Phytochem. Anal.*, 2021: *32*, 38-61 (DOI: 10.1002/pca.2946). Copyright 2021 John Wiley & Sons. Order Number: 5776410703091.

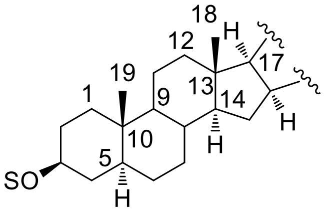

Structural Features								HMBC signals									
C-2	C-5	C-6	C-9	C-12	C-22	C-23	C-24	**H-18**	C-12	C-13	C-14	C-17	**H-19**	C-1	C-5	C-9	C-10
	α				SP			0.80	40.0	40.5	56.1	62.9	0.62	36.9	44.4	54.1	35.6
	α				SP	OH*α*	OGlc*β*	1.01	40.7	41.4	56.6	62	0.75	37.5	45.6	54.6	35.9
	α			CO	SP			1.03	212.8	55.4	55.9	54.3	0.61	36.6	44.4	55.5	36.3
	α		DB	CO	SP			0.98	204.3	51.3	52.7	54.5	0.79	35.0	42.5	171.3	39.5
OH *α*	α		DB	CO	SP			0.97	204.3	51.4	52.7	54.6	0.86	43.5	42.5	170.5	40.6
	α			OH*β*	SP			1.06	79.3	46.6	55.2	63.0	0.64	37.2	44.7	53.6	35.9
OH *α*	α				SP			0.78	40.0	40.6	56.3	63.0	0.69	45.6	44.6	54.3	36.8
	β				SP			0.79	40.3	40.9	56.5	63.1	0.84	30.8	36.9	40.2	35.2
OH *β*	β				SP			0.77	40.2	40.8	56.3	63.1	0.87	40.5	36.4	41.4	36.9
	DB				SP			0.80	39.9	40.5	56.7	62.9	0.85	37.5	141.1	50.3	37.1
	DB			CO	F			1.13	212.9	55.4	56.0	54.9	0.91	37.0	140.9	52.4	37.6
OH *α*	DB				SP			0.78	39.8	40.5	56.5	62.9	0.91	45.8	140.1	50.2	38.0
OH *α*	DB				SP		OGlc*β*	0.71	39.7	40.4	56.5	62.3	0.91	45.7	140.1	50.1	37.9
OH *α*	DB				F			0.85	39.9	40.8	56.5	63.9	0.92	45.8	140.1	50.3	38.0
OH *α*	DB				FM			0.77	39.6	40.8	56.4	64.1	0.91	45.7	140.1	50.2	37.9
	α	OH*α*			SP			0.81	40.0	40.7	56.2	62.9	0.68	37.6	52.1	54.0	36.4
	α	OGlc*α*			SP			0.76	40.0	40.8	56.4	63.0	0.68	37.5	50.9	53.8	36.7
	α	OGlc*α*			SP	OH*α*		0.96	22.7	41.4	56.5	62.6	0.74	37.8	51.3	54.0	36.8

OH: hydroxyl; DB: double bond; CO: carbonyl; SP: spirostanic; F: furostanic; *α*/*β*: chiral center configuration.

Table S3. Expected (data from pure compounds) and experimental correlations of HSQC-TOCSY of sugar chains (S4, S5Xyl, S5Rha, and S5Api) of *Agave* saponin-rich fractions.

NMR data	Pure compounds	*A. colorata*	*A. macroacantha*	*A. parryi*	*A. parrasana*
**Gal-1 (C-2 H)**	**4.83-4.86**	**4.84**	**4.82**	**4.82**	**4.85**
**Gal-1 (C-2 H)**	102.5	102.2	102.2	102.1	102.2
**Gal-2 (C-2 H)**	73.2	72.9	72.8	72.8	73.1
**Gal-3 (C-2 H)**	75.6	75.3	75.3	75.2	75.2
**Gal-4 (C-2 H)**	79.8	79.5	79.5	79.5	79.5
**Gal-6 (C-2 H)**	60.8	60.4	60.5	60.3	60.6
**Gal-1 (C-2 OH)**	**4.89**	-	**4.87**	**4.87**	**4.90**
**Gal-1 (C-2 OH)**	103.2	-	102.9	102.9	103.0
**Gal-2 (C-2 OH)**	72.5	-	72.2	72.0	72.3
**Gal-3 (C-2 OH)**	75.6	-	75.3	75.1	75.4
**Gal-4 (C-2 OH)**	79.0	-	78.7	-	79.1
**Gal-6 (C-2 OH)**	60.8	-	60.5	60.3	60.6
**Xyl'-1 (S5Xyl)**	**5.06**	**5.06**	-	**5.05**	**5.07**
**Xyl'-1 (S5Xyl)**	106.1	106.0	-	105.8	106.1
**Xyl'-2 (S5Xyl)**	75.4	75.3	-	75.1	75.4
**Xyl'-3 (S5Xyl)**	77.7	77.6	-	77.5	77.6
**Xyl'-4 (S5Xyl)**	70.7	70.5	-	70.4	70.6
**Xyl'-5 (S5Xyl)**	67.0	67.0	-	66.9	67.0
**Xyl-1 (S5)**	**5.11-5.15**	**5.12**	**5.10**	**5.10**	**5.13**
**Xyl-1 (S5)**	104.8-105.0	104.7	104.6	104.5	104.7
**Xyl-2 (S5)**	75.1-75.3	74.9	75.0	74.8	75.1
**Xyl-3 (S5)**	78.4-78.7	78.2	78.3	78.1	78.3
**Xyl-4 (S5)**	70.7	70.5	70.4	70.3	70.6
**Xyl-5 (S5)**	67.3	67.0	67.0	67.0	67.0
**Glc-1**	**5.15-5.22**	**5.16**	**5.14, 5.19**	**5.13-5.17**	**5.16-5.20**
**Glc-1**	104.3-105.3	104.6	104.0, 104.5	104.5	104.5
**Glc-2**	80.7-81.5	80.6	80.5, 80.7	80.4	80.9
**Glc-3**	86.8-87.3	86.5	86.9	86.5	86.7
**Glc-4**	70.4-70.6	70.2	70.1	70.1	70.4
**Glc-5**	77.5-77.9	77.4	77.3, 77.4	77.2	77.4
**Glc-6**	63.0	62.8	62.7	62.7	62.8
**Xyl-1 (S4)**	**5.22-5.23**	**5.22**	**5.22**	**5.21**	**5.24**
**Xyl-1 (S4)**	104.9-105.1	104.7	-	104.5	104.7
**Xyl-2 (S4)**	75.1	74.8	-	74.7	75.1
**Xyl-3 (S4)**	78.7	78.5	-	78.3	78.6
**Xyl-4 (S4)**	70.7	70.5	-	70.4	70.6
**Xyl-5 (S4)**	67.3-67.5	67.1	-	67.0	67.2
**Glc´-1 (S4/5Xyl)**	**5.56-5-57**	**5.56**	**5.56**	**5.53, 5.54**	**5.57, 5.58**
**Glc´-1 (S5Rha/Api)**	**5.48-5.49**	**5.47, 5.48**	**5.46**	**5.46, 5.48**	**5.50**
**Glc´-1 (S4)**	105.0-104.7	104.6	-	104.5	104.5
**Glc´-1 (S5Xyl)**	103.9-104.1	103.8	-	103.8	-
**Glc´-1 (S5 Rha/Api)**	104.4	104.1	104.1	103.9	104.0
**Glc´-2**	75.5-76.6	74.9, 76.1	76.1	75.2, 75.8	75.3, 76.0
**Glc´-3 (S4)**	77.8-78.1	78.2	78.0	78.1	78.2
**Glc´-3 (S5Xyl)**	86.8-87.1	86.5	-	86.4	86.8
**Glc´-3 (S5Rha)**	83.2-83.5	82.8	82.9	82.7	-
**Glc´-3 (S5Api)**	84.5	84.1	84.2	84.1	84.2
**Glc´-4**	69.2-71.1	68.8, 70.7	69.0	68.7, 70.7	69.4, 71.2
**Glc´-5**	77.7-78.5	78.2	78.2	77.9, 78.1	78.2
**Glc´-6**	63.0-62.3	61.9	62.0	62.0	62.6
**Rha-1 (S5Rha)**	**6.08-6.10**	**6.09**	**6.07**	**6.06**	**-**
**Rha-1 (S5Rha)**	102.8	102.5	102.5	102.4	-

**Rha-2 (S5Rha)**	72.5	72.1	72.1	72.0	-
**Rha-3 (S5Rha)**	72.7	72.3	72.3	72.1	-
**Rha-4 (S5Rha)**	74.2	73.8	73.8	73.7	-
**Rha-5 (S5Rha)**	69.8	69.5	69.5	69.4	-
**Rha-6 (S5Rha)**	**1.62**	**1.62**	**1.60**	**1.59**	**-**
**Rha-6 (S5Rha)**	18.7	18.4	18.4	18.2	-
**Api-1 (S5Api)**	**6.08**	**6.08**	**6.07**	**6.06**	**6.08**
**Api-1 (S5Api)**	111.5	111.1	111.1	111.0	111.2
**Api-2 (S5Api)**	77.7	77.3	77.3	77.2	77.4
**Api-5 (S5Api)**	**4.12**	4.12	4.11	4.10	4.12
**Api-5 (S5Api)**	65.7	65.4	65.3	65.2	65.5

Table S4. Expected (data from pure compounds) and experimental correlations of HMBC of sugar chains (S4, S5Xyl, S5Rha, and S5Api) of *Agave* saponin-rich fractions.

NMR data	Pure compounds	*A. colorata*	*Agave macroacantha*	*A. parryi*	*A. parrasana*
**Gal-1 (C-2 H)**	**4.83-4.86**	**4.84**	**4.82**	**4.83**	**4.84**
**Gal-3/5 (C-2 H)**	75.6, 75.5	75.3	75.5	75.1	75.3
**Aglic-3 (C-2 H)**	76.7-77.2	77.0	77.0	76.9	77.1
**Gal-1 (C-2 OH)**	**4.89**	-	**4.87**	**4.87**	**4.90**
**Gal-3/5 (C-2 OH)**	75.9	-	75.6	75.3	75.6
**Aglic-3 (C-2 OH)**	83.5-83.9	-	83.7	83.5	83.9
**Xyl'-1 (S5Xyl)**	**5.06**	**5.07**	-	**5.04**	**5.07**
**Xyl'-3 (S5Xyl)**	77.7	-	-	77.5	-
**Xyl'-5 (S5Xyl)**	67.0	67.0	-	66.9	-
**Glc´-3 (S5Xyl)**	86.8-87.1	86.6	-	86.6	87.0
**Xyl-1 (S5)**	**5.11-5.15**	**5.12**	**5.10**	**5.10-5.12**	**5.13**
**Xyl-3 (S5)**	78.4-78.7	78.2	78.6	78.2	78.4
**Xyl-5 (S5)**	67.3	67.2	67.2	67.0	67.3
**Glc-3**	86.8-87.3	86.6	87.1	86.6	87.0
**Glc-1**	**5.15-5.22**	**5.17**	**5.14, 5.19**	**5.13-5.17**	**5.15-5.20**
**Glc-3**	86.8-87.3	86.6	87.1	86.6	87.0
**Glc-5**	77.1-77.9	77.8	77.5, 77.6	77.5	77.9
**Gal-4 (C-2 H)**	79.8	79.5	79.7	79.6	79.8
**Gal-4 (C-2 OH)**	79.0	-	78.9	79.1	79.4
**Xyl-1 (S4)**	**5.22-5.23**	**5.22**	**5.22**	**5.21**	**5.24**
**Xyl-3 (S4)**	78.7	78.6	-	78.4	78.7
**Xyl-5 (S4)**	67.3-67.5	67.3	-	67.2	67.6
**Glc-3**	86.8-87.3	86.6	86.8	86.6	87.0
**Glc´-1 (S4/5Xyl)**	**5.56-5-57**	**5.56**	**5.55**	**5.54**	**5.57**
**Glc´-1 (S5Rha/Api)**	**5.48-5.49**	**5.47, 5.48**	**5.46**	**5.47**	**5.50**
**Glc´-3 (S4)**	77.8-78.1	77.9	-	78.0	78.2
**Glc´-3 (S5Rha)**	83.2-83.5	-	83.2	-	-
**Glc´-5**	77.7-78.9	78.8	78.4	-	78.2
**Glc-2**	80.7-81.5	80.9	80.9, 81.3 (S4)	80.4	80.8, 81.3 (S4)
**Rha-1 (S5Rha)**	**6.08-6.10**	**6.09**	**6.07**	**6.06**	**-**
**Rha-3 (S5Rha)**	72.7	72.5	72.5	72.2	-
**Rha-5 (S5Rha)**	69.8	69.5	69.8	69.4	-
**Glc´-3 (S5Rha)**	83.2-83.5	83.0	83.2	83.0	-
**Api-1 (S5Api)**	**6.08**	**6.08**	**6.07**	**6.06**	**6.09**
**Api-3 (S5Api)**	80.5	-	-	80.2	80.5
**Api-5 (S5Api)**	75.2	75.2	75.1	74.9	75.1
**Glc´-3 (S5Api)**	84.5	84.5	84.5	84.1	84.4

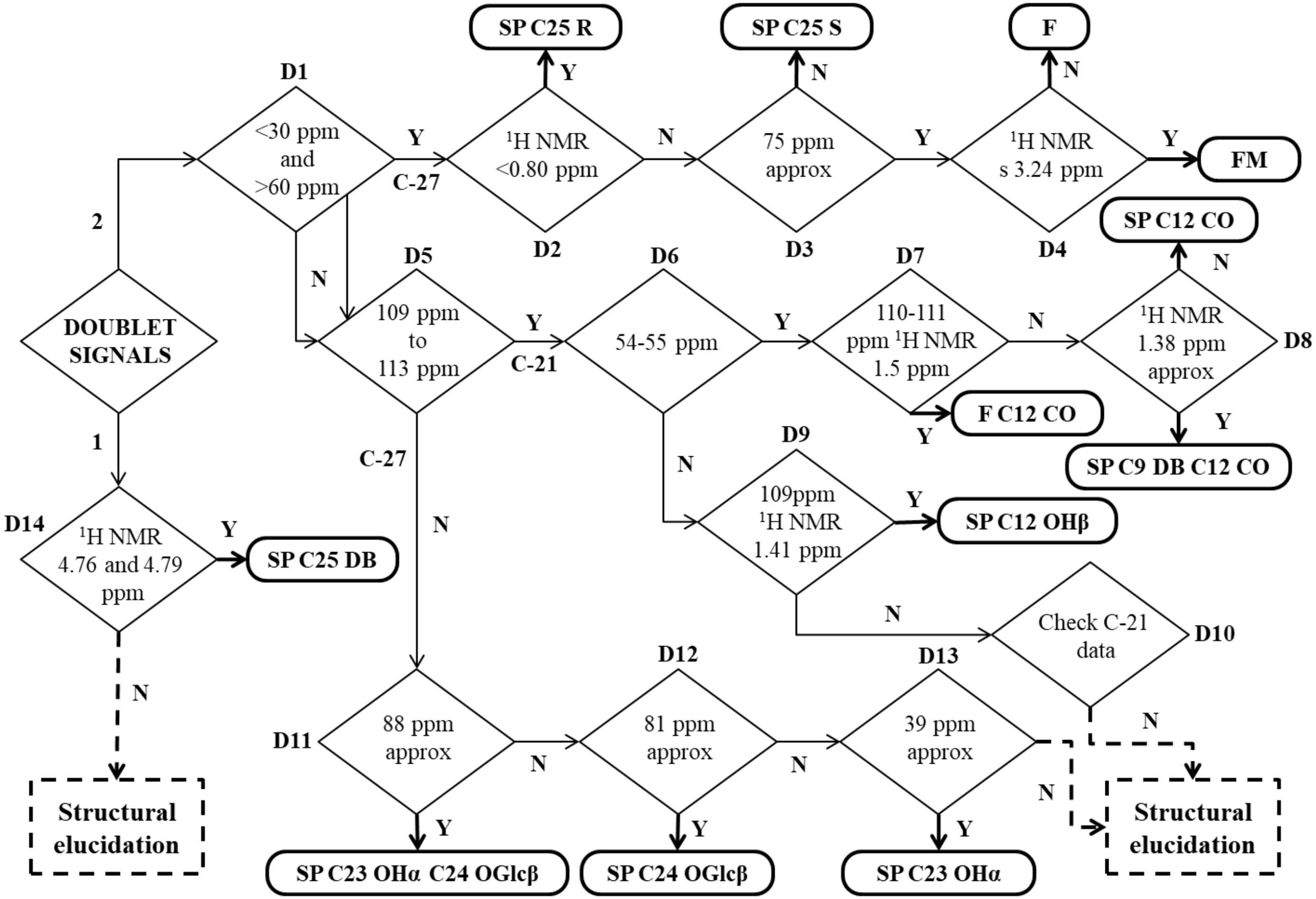

SP: spirostanic; F furostanic; FM: methoxyfurostanic; R/S/α/β quiral configuration; CO: carbonyl group; OH: hydroxyl group; DB: double bound; Oglc: glucopyranosyloxy; Y: Yes; N: Not.

Figure S1. HMAI Flowchart of doublets. v 2024. Reprinted with permission from Simonet *et al., Phytochem. Anal.*, 2021: *32*, 38-61 (DOI: 10.1002/pca.2946). Copyright 2021 John Wiley & Sons. Order Number: 5776410703091.

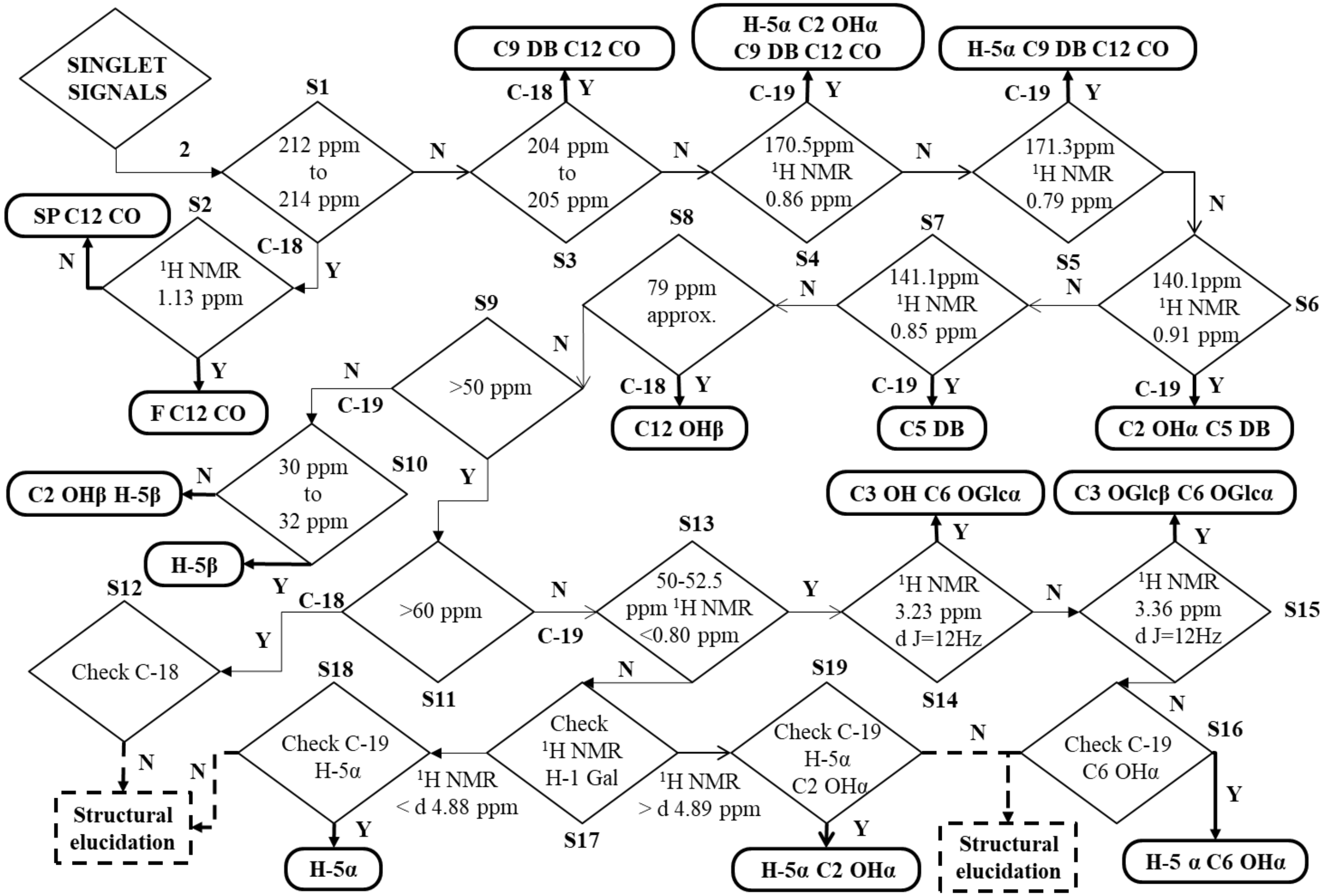

SP: spirostanic; F furostanic; R/S/α/β quiral configuration; CO: carbonyl group; OH: hydroxyl group; DB: double bound; Oglc: glucopyranosyloxy; Y: Yes; N: Not.

Figure S2. HMAI Flowchart of singlets. v 2024. Reprinted with permission from Simonet *et al.*, *Phytochem. Anal.*, 2021: *32*, 38-61 (DOI: 10.1002/pca.2946). Copyright 2021 John Wiley & Sons. Order Number: 5776410703091.

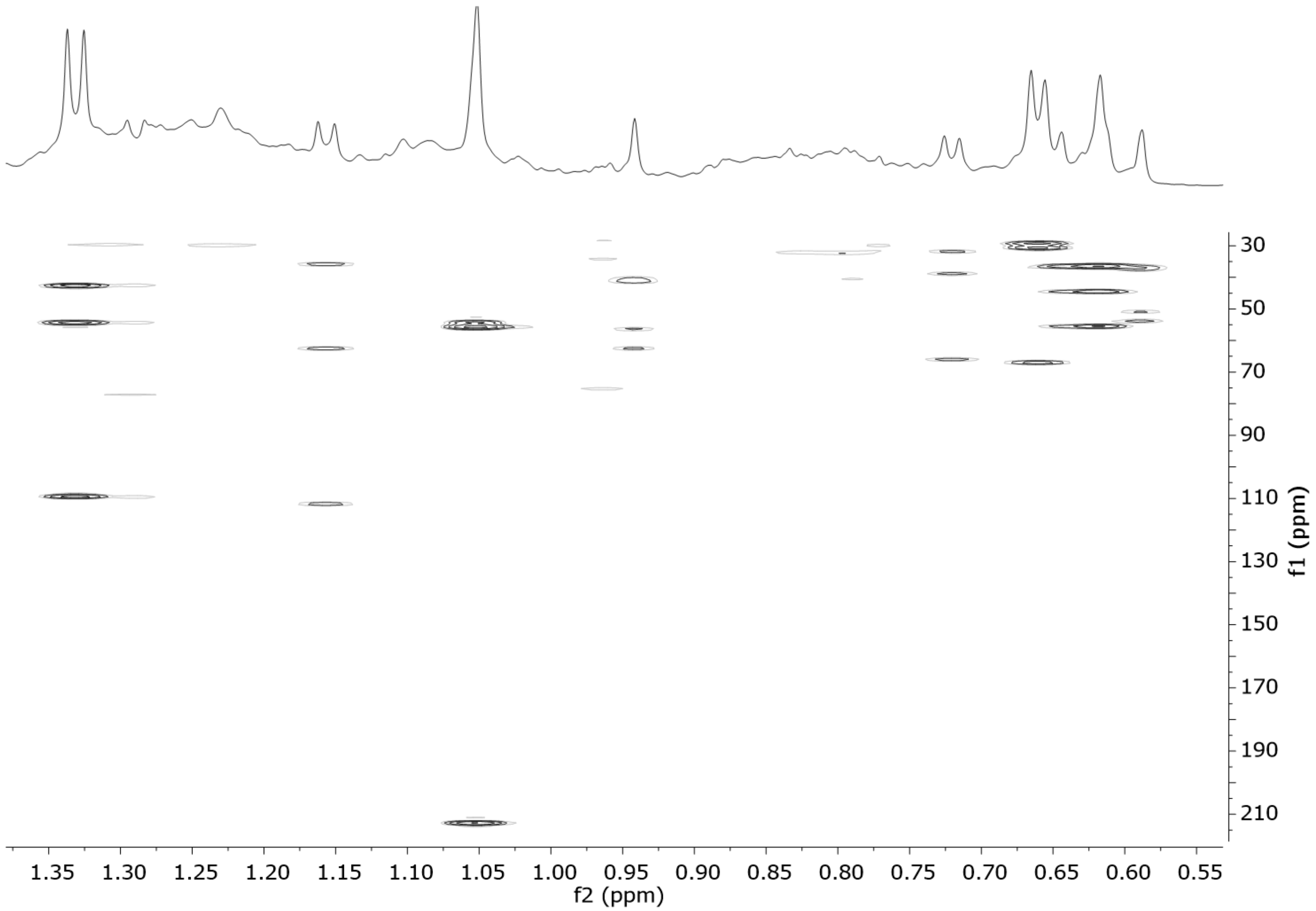

Figure S3. HMBC of saponin-enriched fraction of *Agave colorata.* Selected area of methyl group signals of aglycone. (600 MHz, Pyridine-d_5).

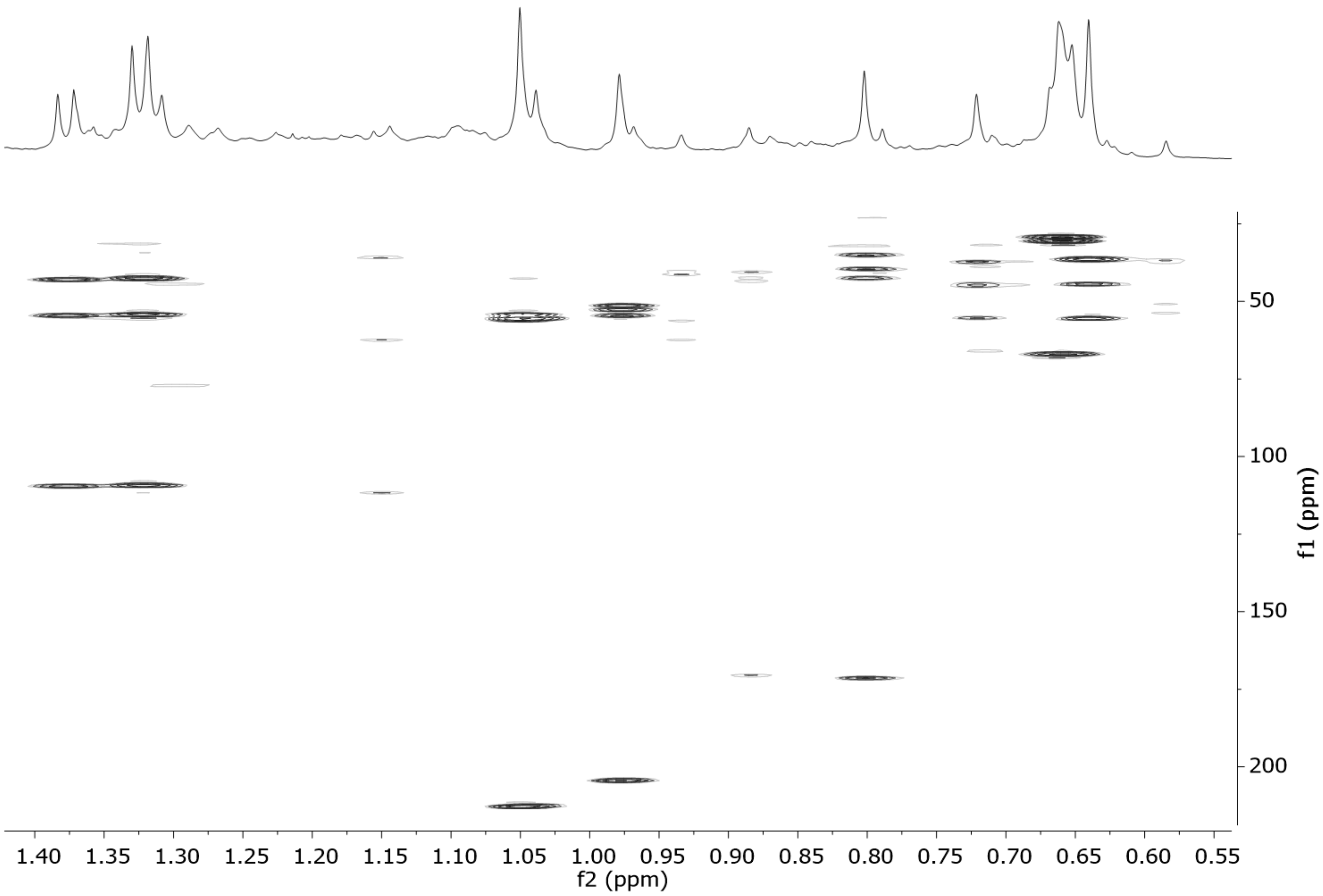

Figure S4. HMBC of saponin-enriched fraction of *Agave macroacantha.* Selected area of methyl group signals of aglycone. (600 MHz, Pyridine-*d*$_5$).

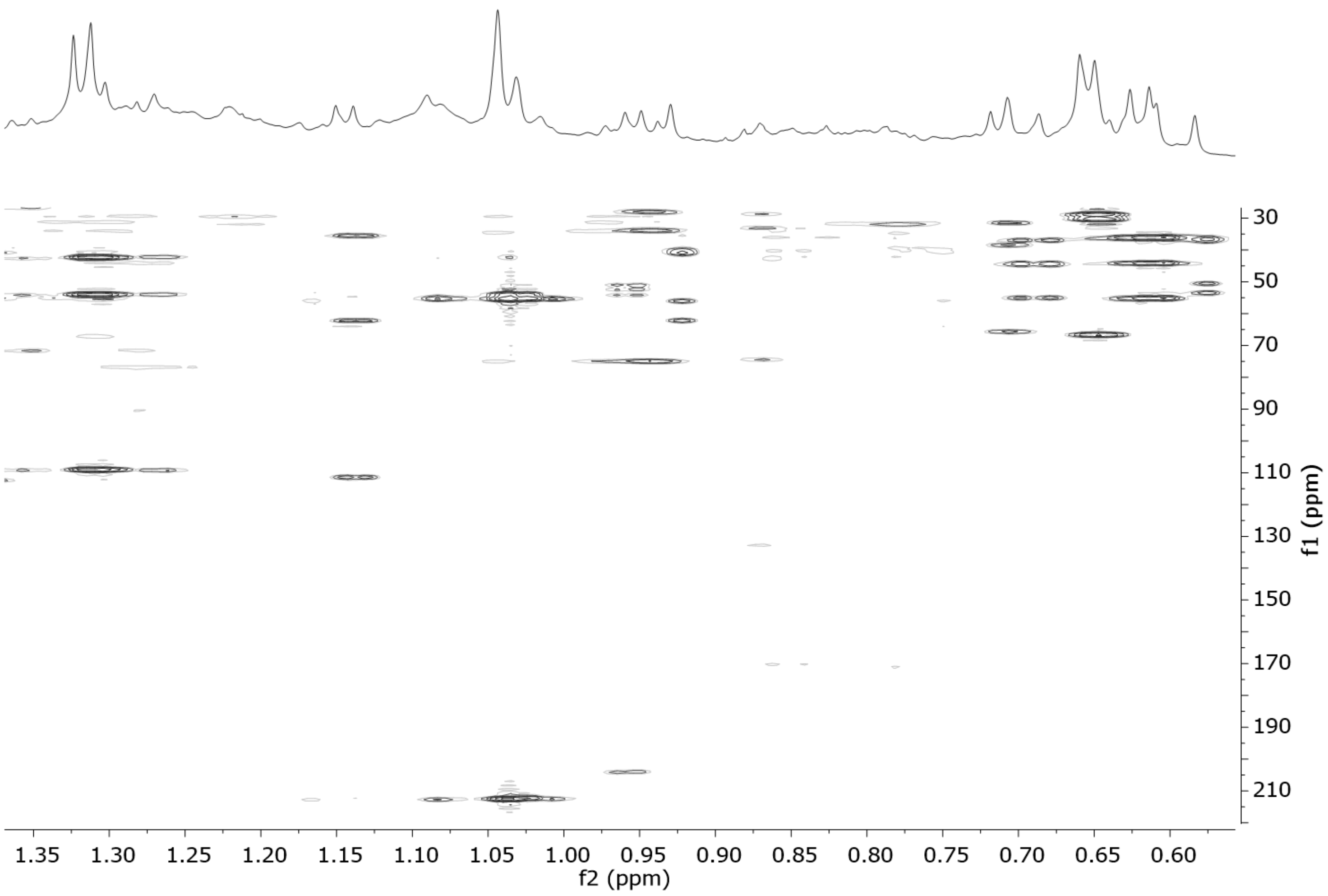

Figure S5. HMBC of saponin-enriched fraction of *Agave parryi.* Selected area of methyl group signals of aglycone. (600 MHz, Pyridine-d_5).

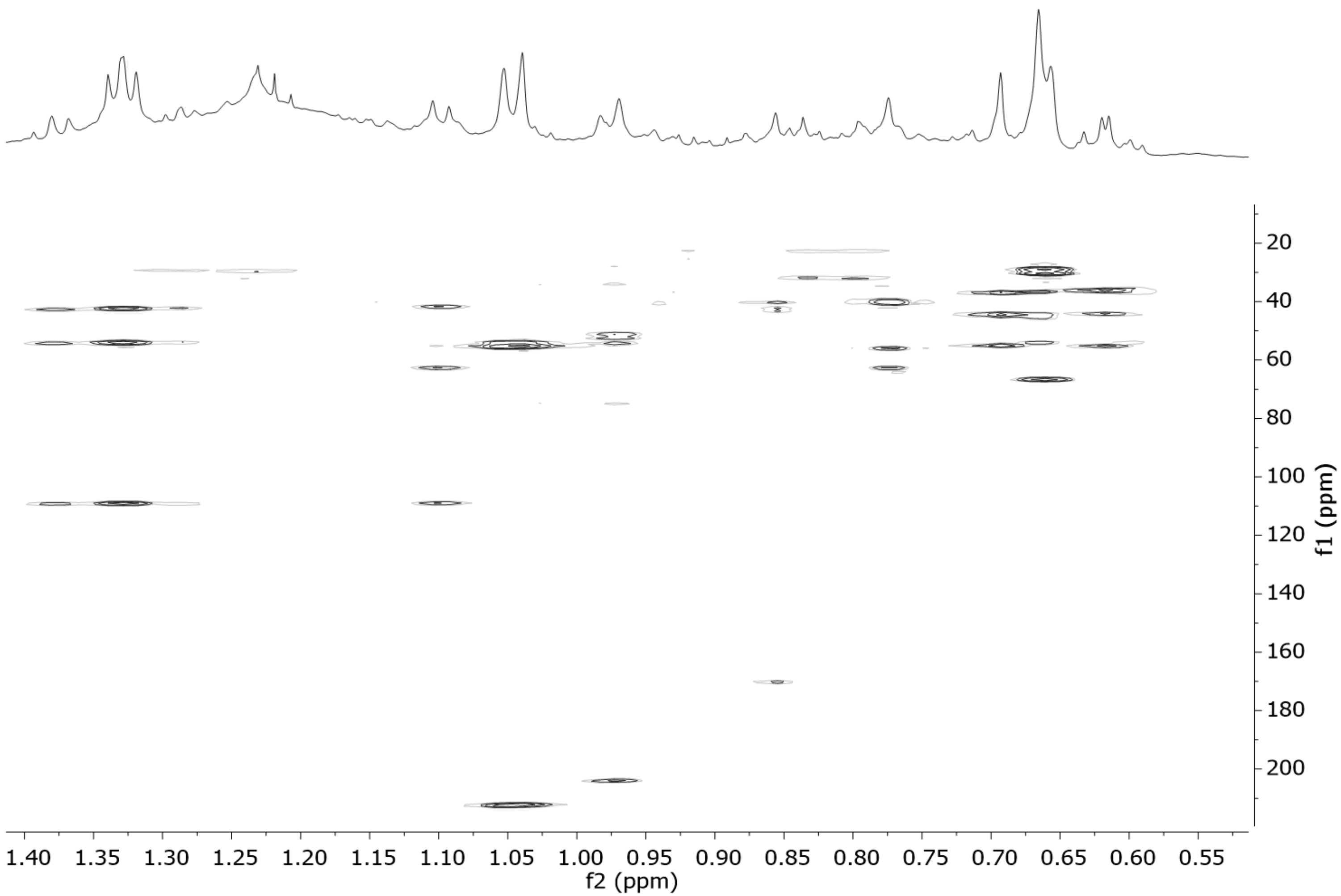

Figure S6. HMBC of saponin-enriched fraction of *Agave parrasana.* Selected area of methyl group signals of aglycone. (600 MHz, Pyridine-d_5).

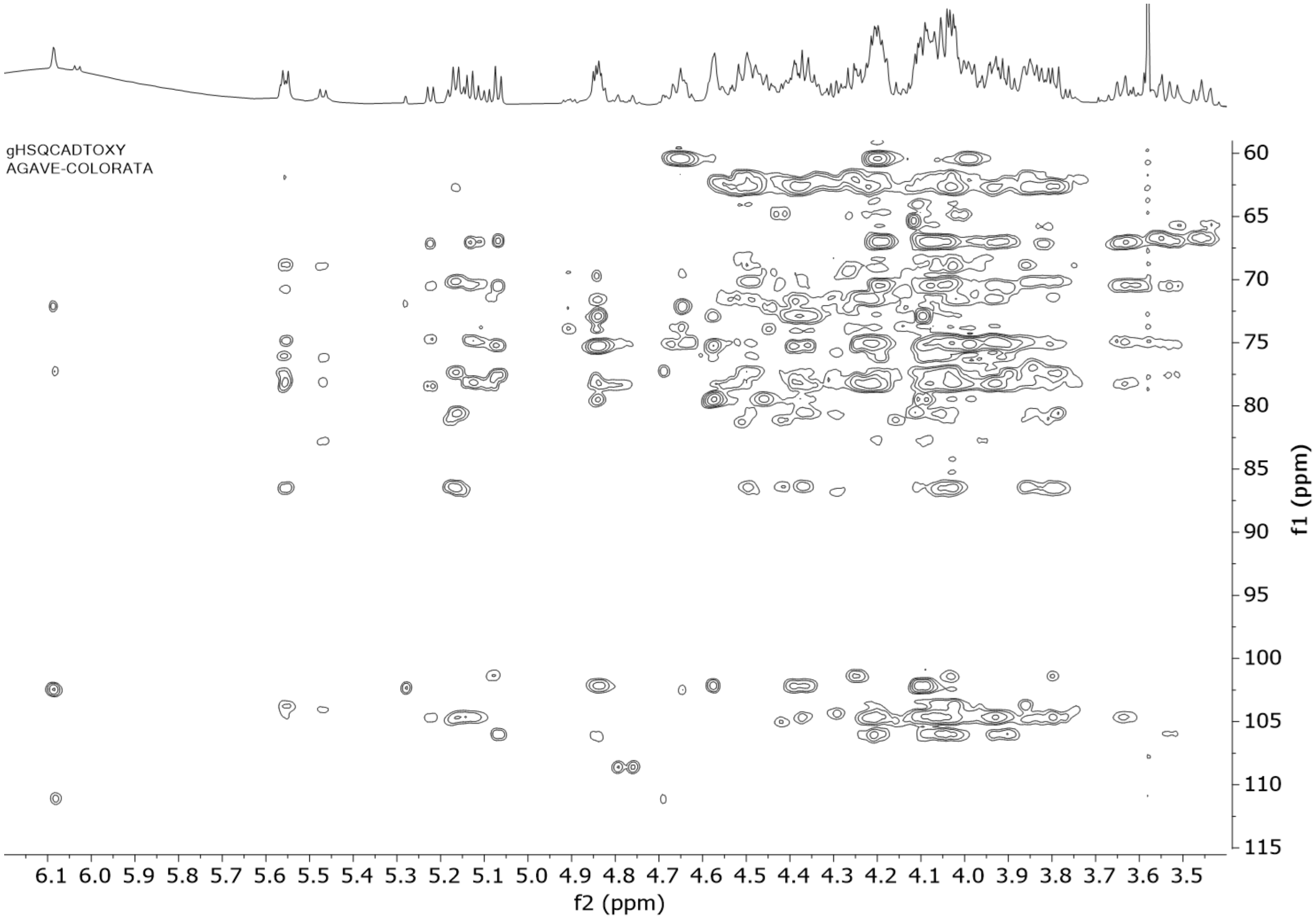

Figure S7. HSQC-TOCSY of saponin-enriched fraction of *Agave colorata.* Selected area of sugar chain signals. (600 MHz, Pyridine-d_5).

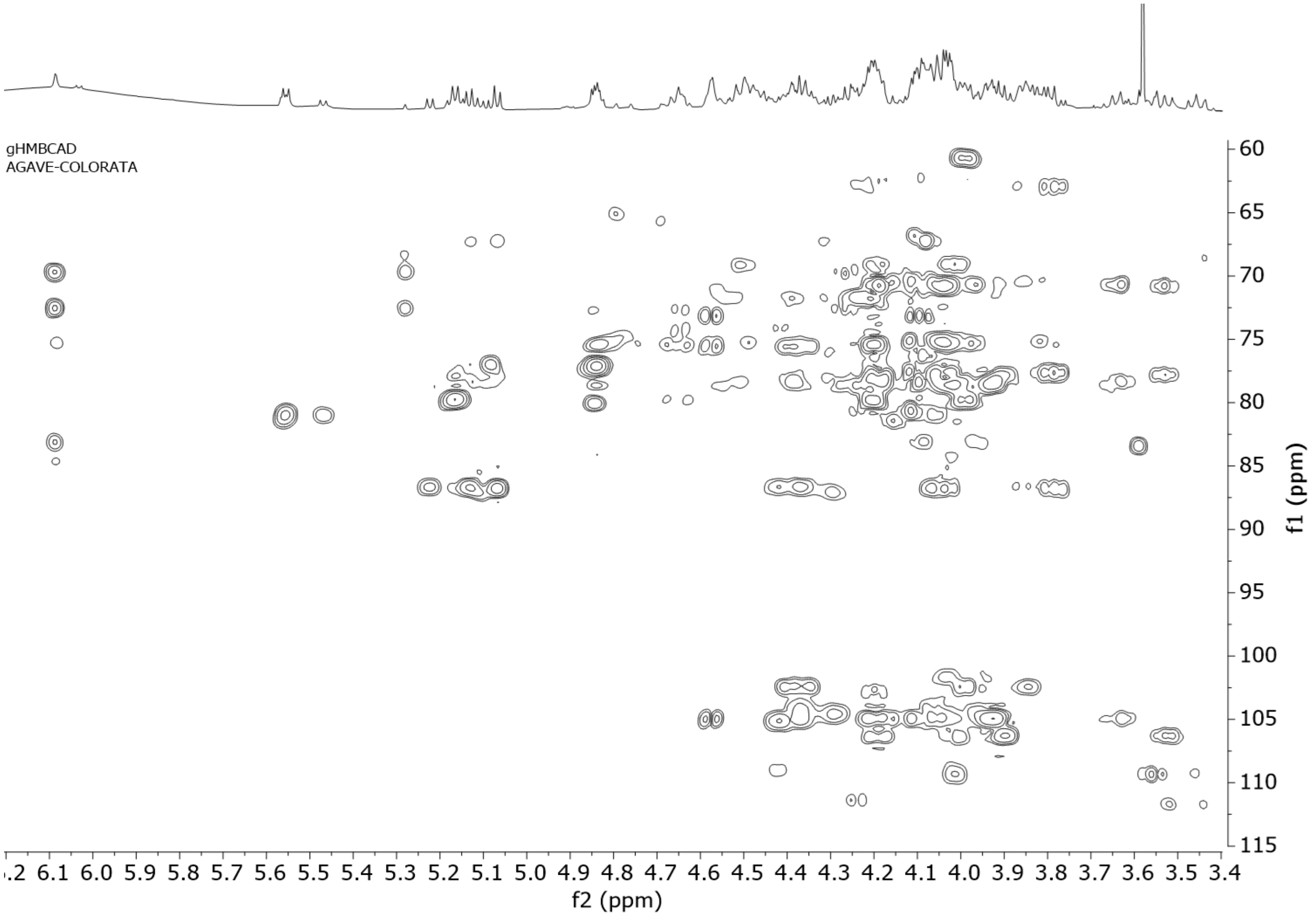

Figure S8. HMBC of saponin-enriched fraction of *Agave colorata.* Selected area of sugar chain signals. (600 MHz, Pyridine-d_5).

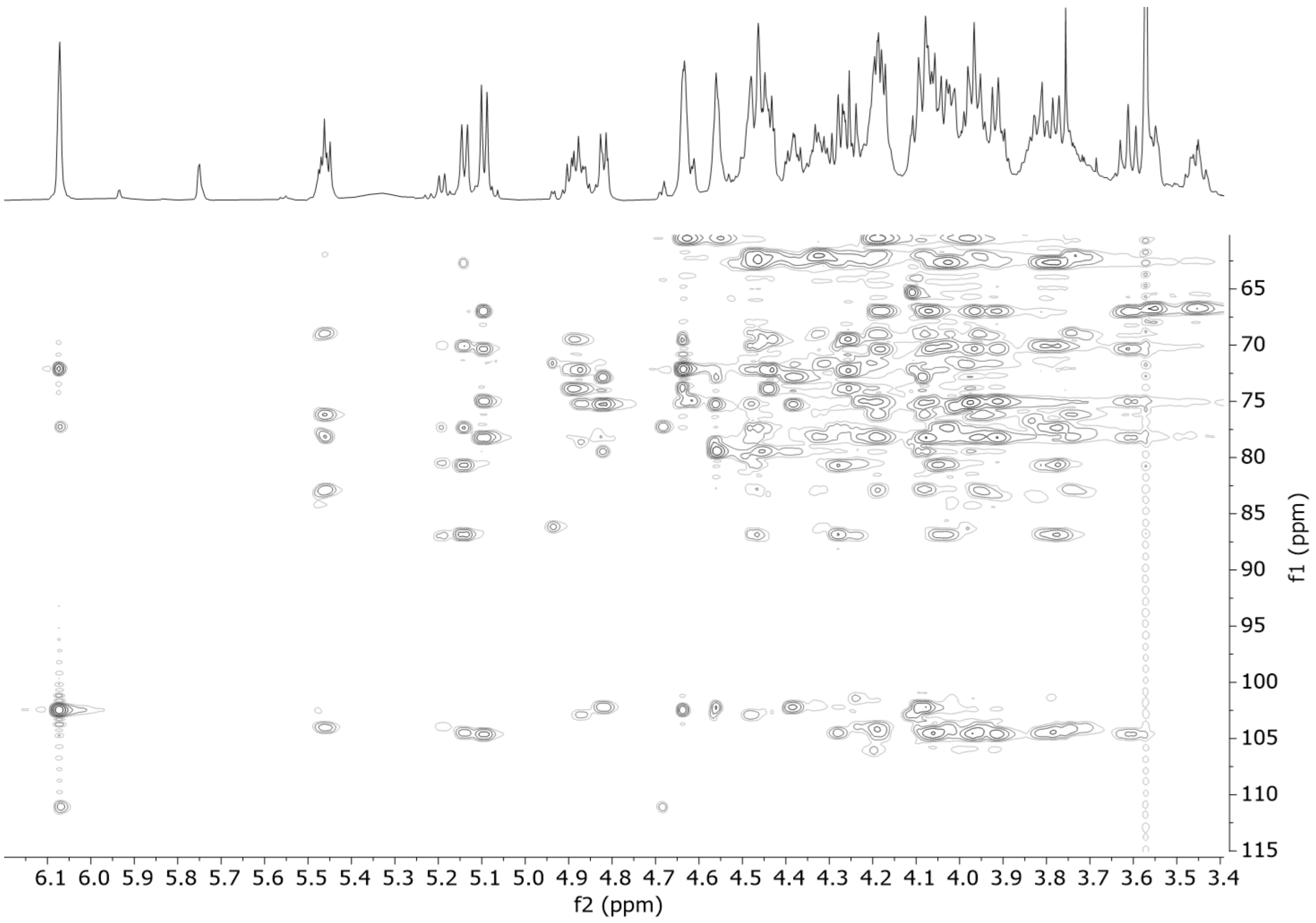

Figure S9. HSQC-TOCSY of saponin-enriched fraction of *Agave macroacantha.* Selected area of sugar chain signals. (600 MHz, Pyridine-d_5).

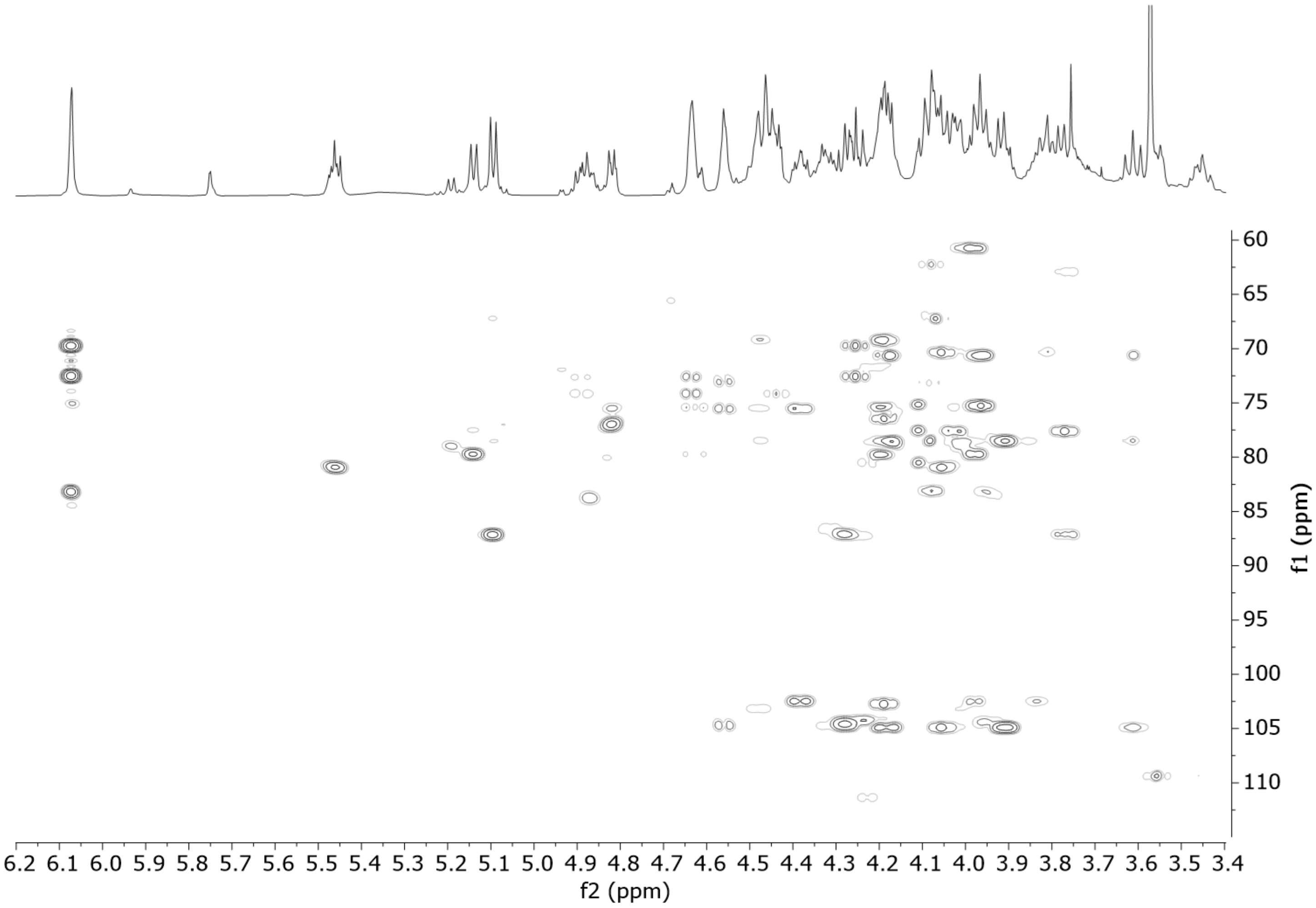

Figure S10. HMBC of saponin-enriched fraction of *Agave macroacantha.* Selected area of sugar chain signals. (600 MHz, Pyridine-d_5).

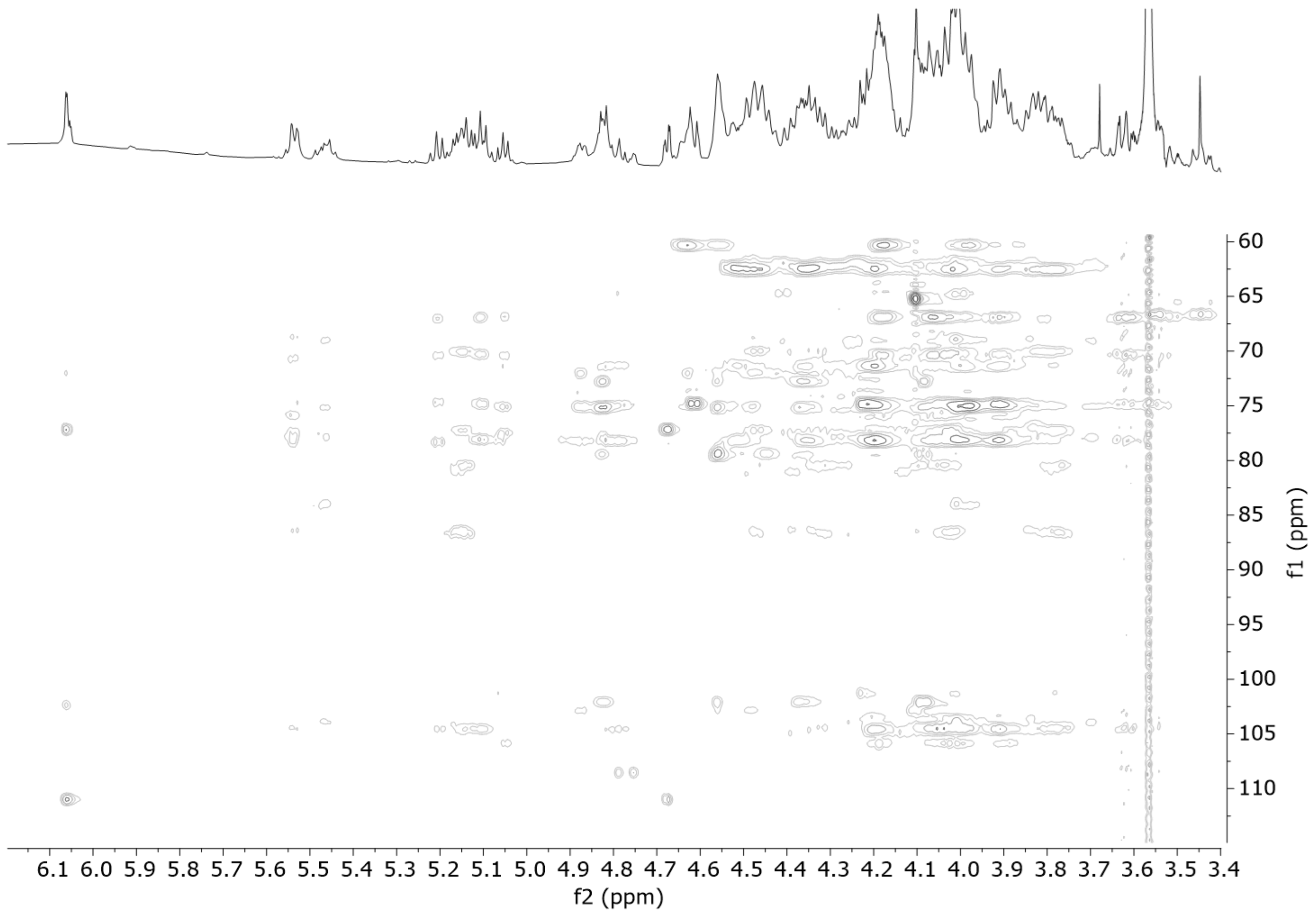

Figure S11. HSQC-TOCSY of saponin-enriched fraction of *Agave parryi.* Selected area of sugar chain signals. (600 MHz, Pyridine-d_5).

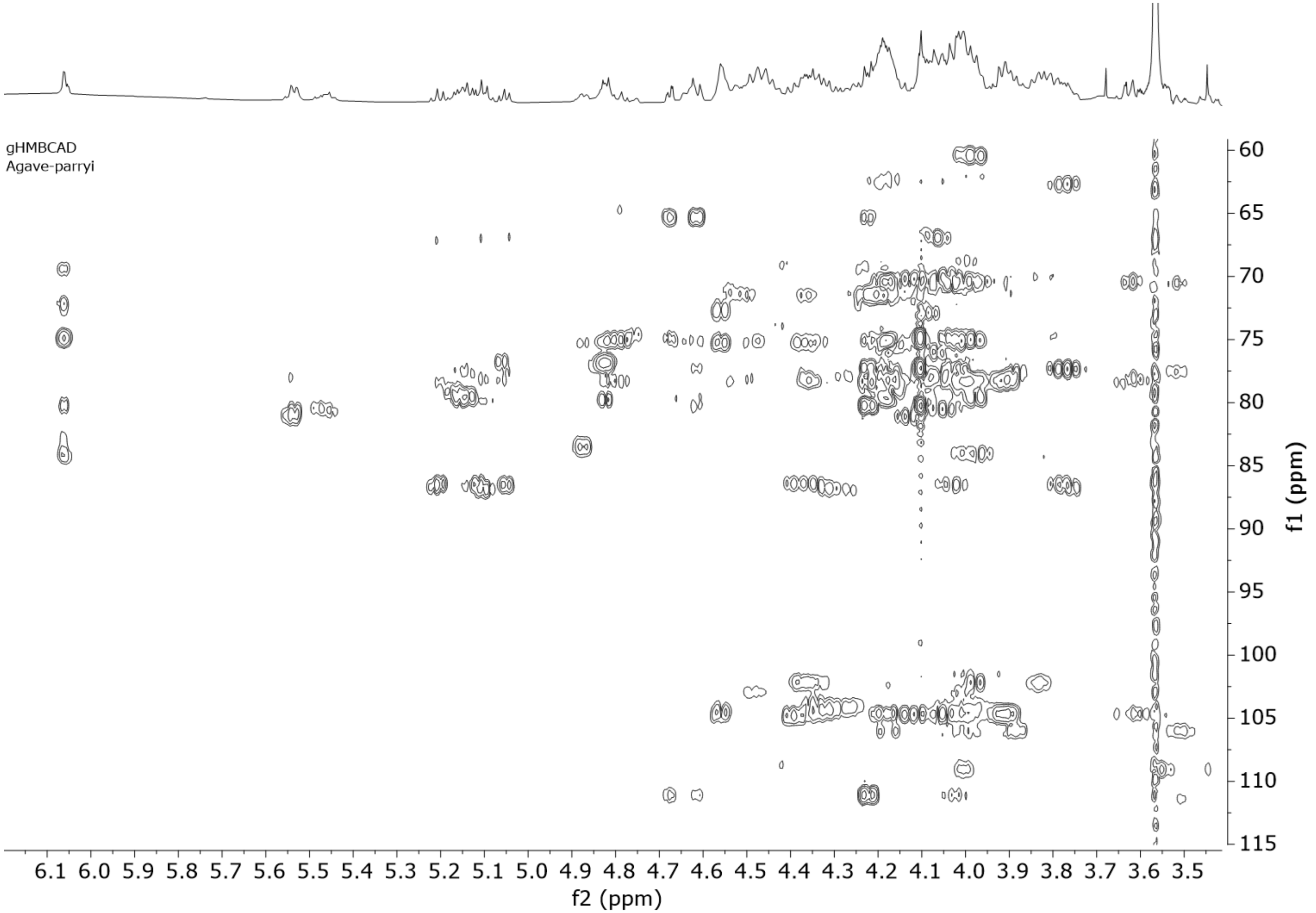

Figure S12. HMBC of saponin-enriched fraction of *Agave parryi.* Selected area of sugar chain signals. (600 MHz, Pyridine-d_5).

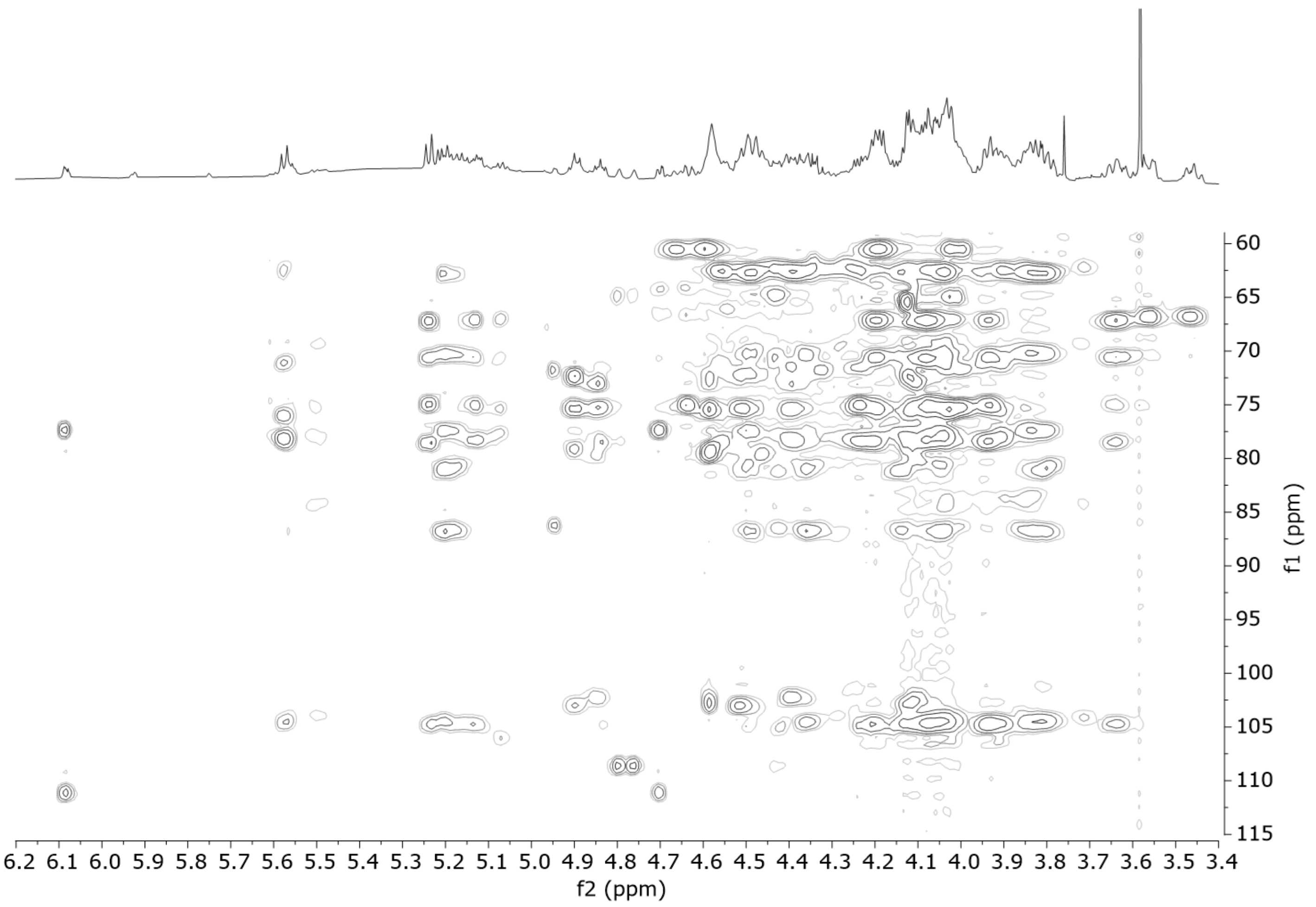

Figure S13. HSQC-TOCSY of saponin-enriched fraction of *Agave parrasana.* Selected area of sugar chain signals. (600 MHz, Pyridine-d_5).

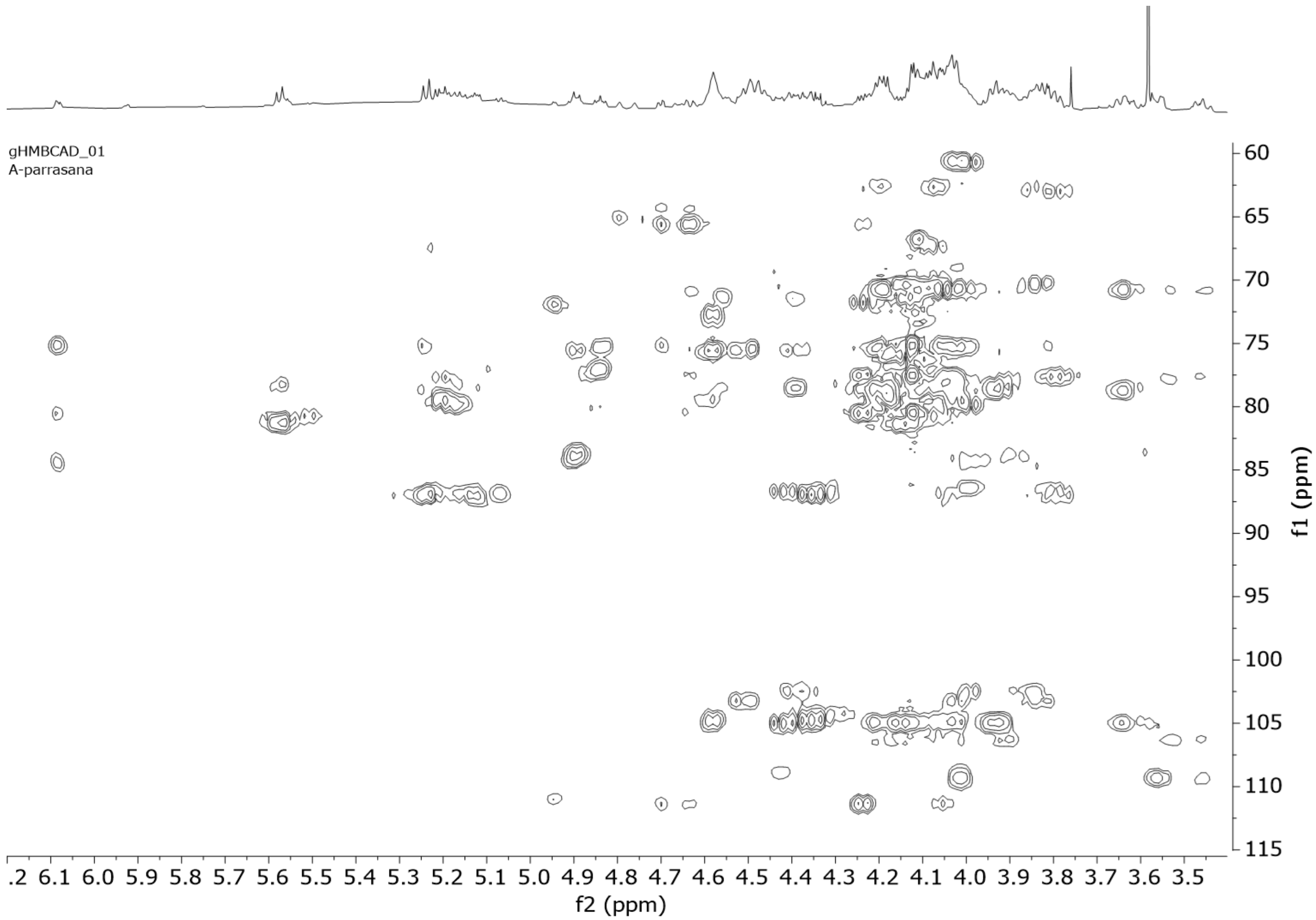

Figure S14. HMBC of saponin-enriched fraction of *Agave parrasana.* Selected area of sugar chain signals. (600 MHz, Pyridine-d_5).

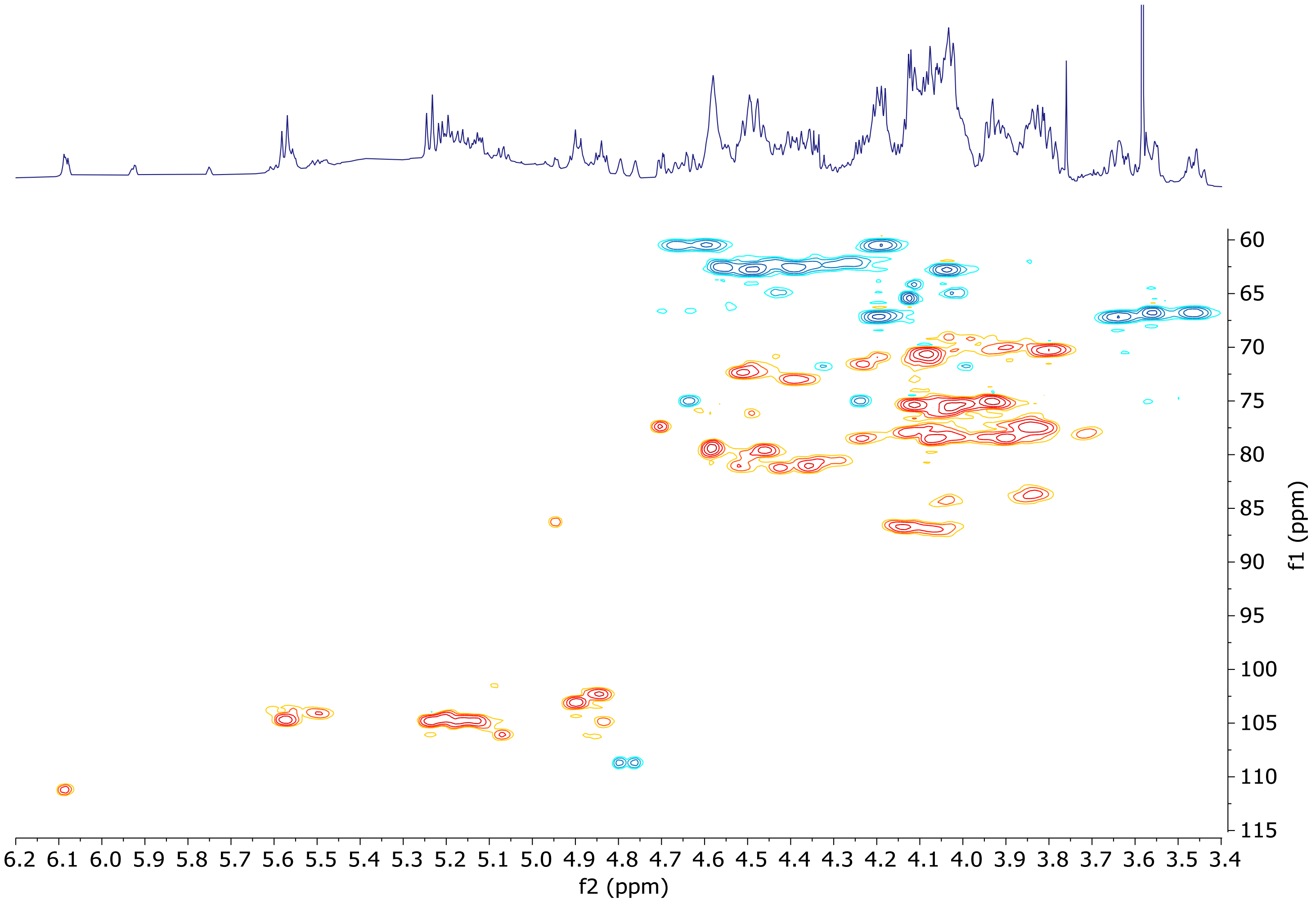

Figure S15. HSQC of saponin-enriched fraction of *Agave parrasana.* Selected area of sugar chain signals. (600 MHz, Pyridine-d_5).

Elemental Composition Report

Tolerance = 5.0 mDa / DBE: min = -1.5, max = 50.0
Element prediction: Off
Number of isotope peaks used for i-FIT = 3

Monoisotopic Mass, Even Electron Ions
477 formula(e) evaluated with 3 results within limits (up to 5 best isotopic matches for each mass)
Elements Used:
C: 0-80 H: 0-1000 O: 0-200

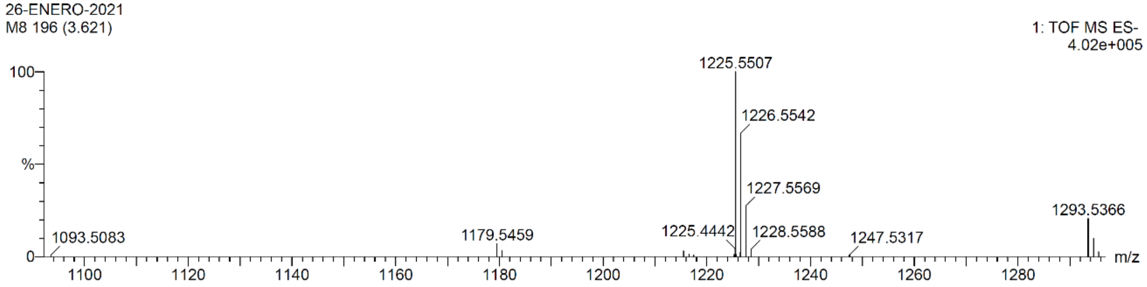

Minimum:	70.00				-1.5				
Maximum:	100.00		5.0	10.0	50.0				
Mass	RA	Calc. Mass	mDa	PPM	DBE	i-FIT	Norm	Conf(%)	Formula
1225.5507	100.00	1225.5490	1.7	1.4	12.5	59.3	0.011	98.88	C56 H89 O29
		1225.5525	-1.8	-1.5	34.5	64.0	4.747	0.87	C74 H81 O16
		1225.5548	-4.1	-3.3	3.5	65.3	5.986	0.25	C49 H93 O34

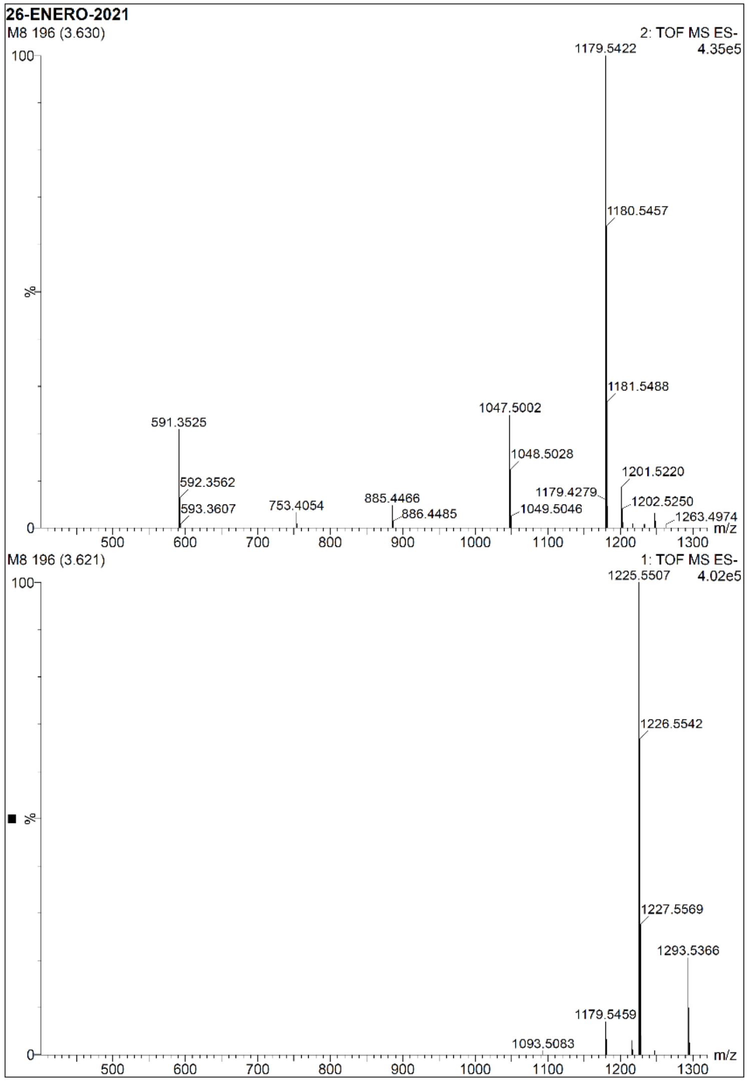

Figure S16. Elemental composition calculated for $C_{51}H_{79}O_{25}$ $[M + CH_3COO^-]^-$ and HRESI MS^E (negative mode) of Coloratoside A (1).

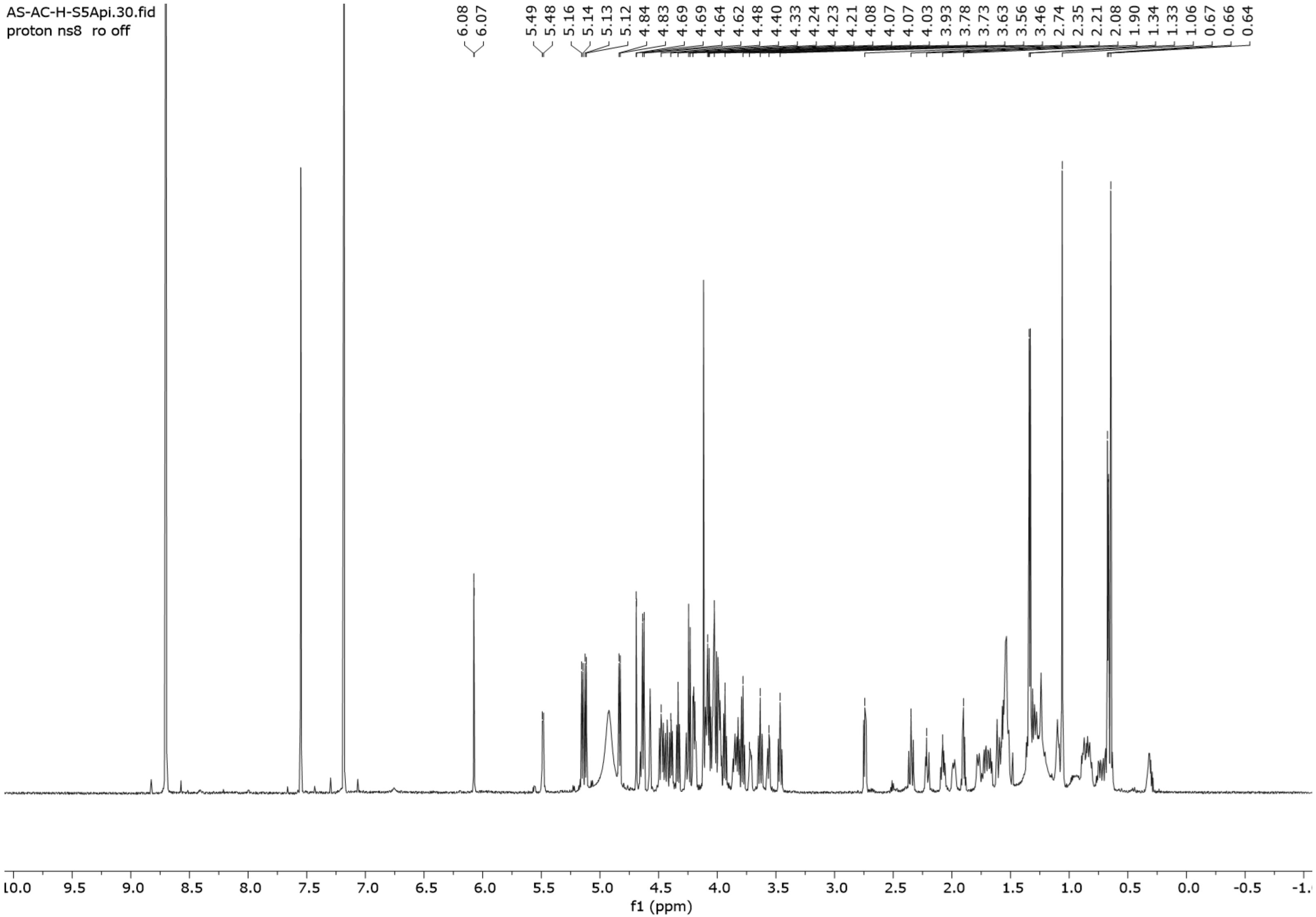

Figure S17. [13]H NMR spectrum of Coloratoside A (1) (700 MHz, Pyridine-d_5)

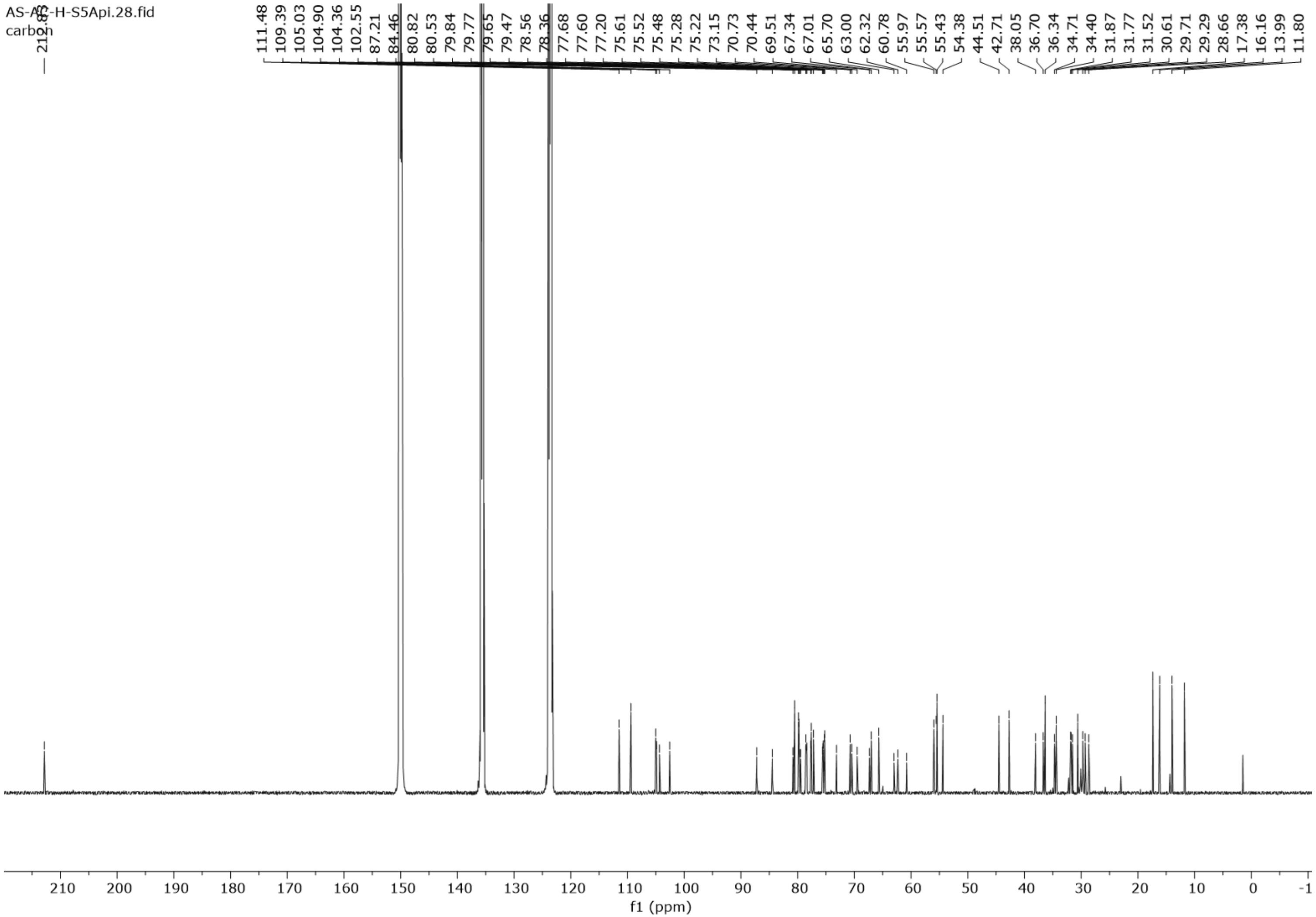

Figure S18. ^{13}C NMR spectrum of Coloratoside A (1) (700 MHz, Pyridine-d_5)

Elemental Composition Report

Tolerance = 5.0 mDa / DBE: min = -1.5, max = 50.0
Element prediction: Off
Number of isotope peaks used for i-FIT = 3

Monoisotopic Mass, Even Electron Ions
487 formula(e) evaluated with 2 results within limits (up to 5 best isotopic matches for each mass)
Elements Used:
C: 0-80 H: 0-1000 O: 0-200

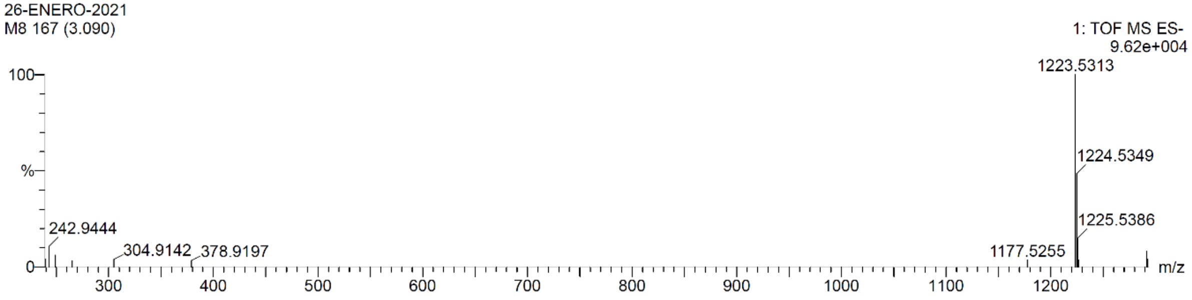

Minimum:	70.00				-1.5				
Maximum:	100.00		5.0	10.0	50.0				
Mass	RA	Calc. Mass	mDa	PPM	DBE	i-FIT	Norm	Conf(%)	Formula
1223.5313	100.00	1223.5333	-2.0	-1.6	13.5	26.2	0.050	95.16	C56 H87 O29
		1223.5274	3.9	3.2	22.5	29.2	3.028	4.84	C63 H83 O24

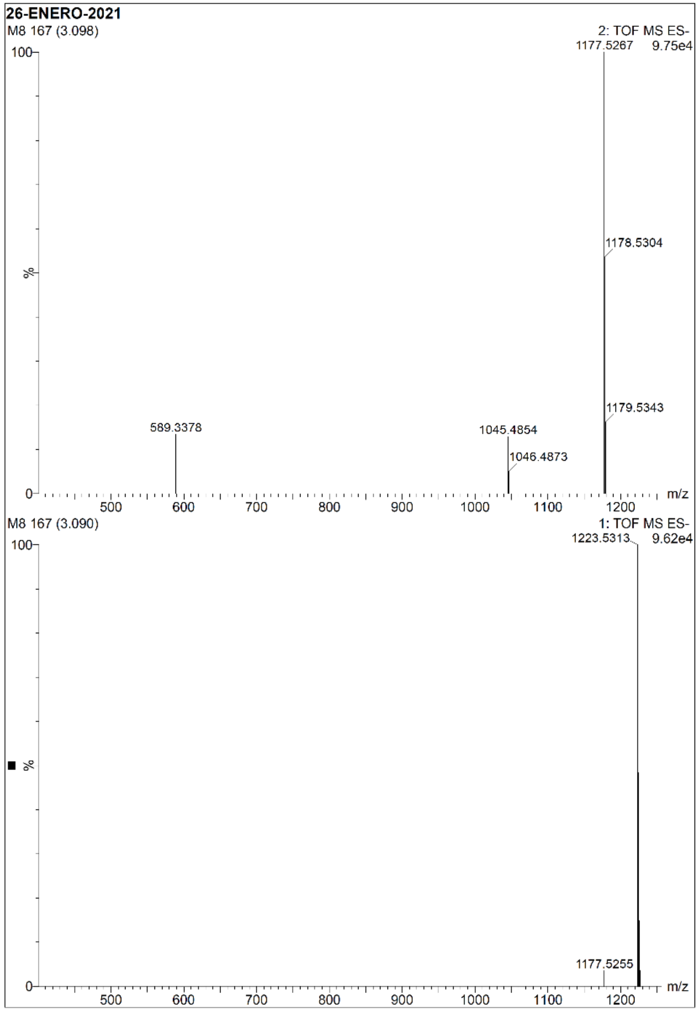

Figure S19. Elemental composition calculated for $C_{51}H_{79}O_{25}$ [M + CH_3COO^-]$^-$ and HRESI MS^E (negative mode) of Coloratoside B (2).

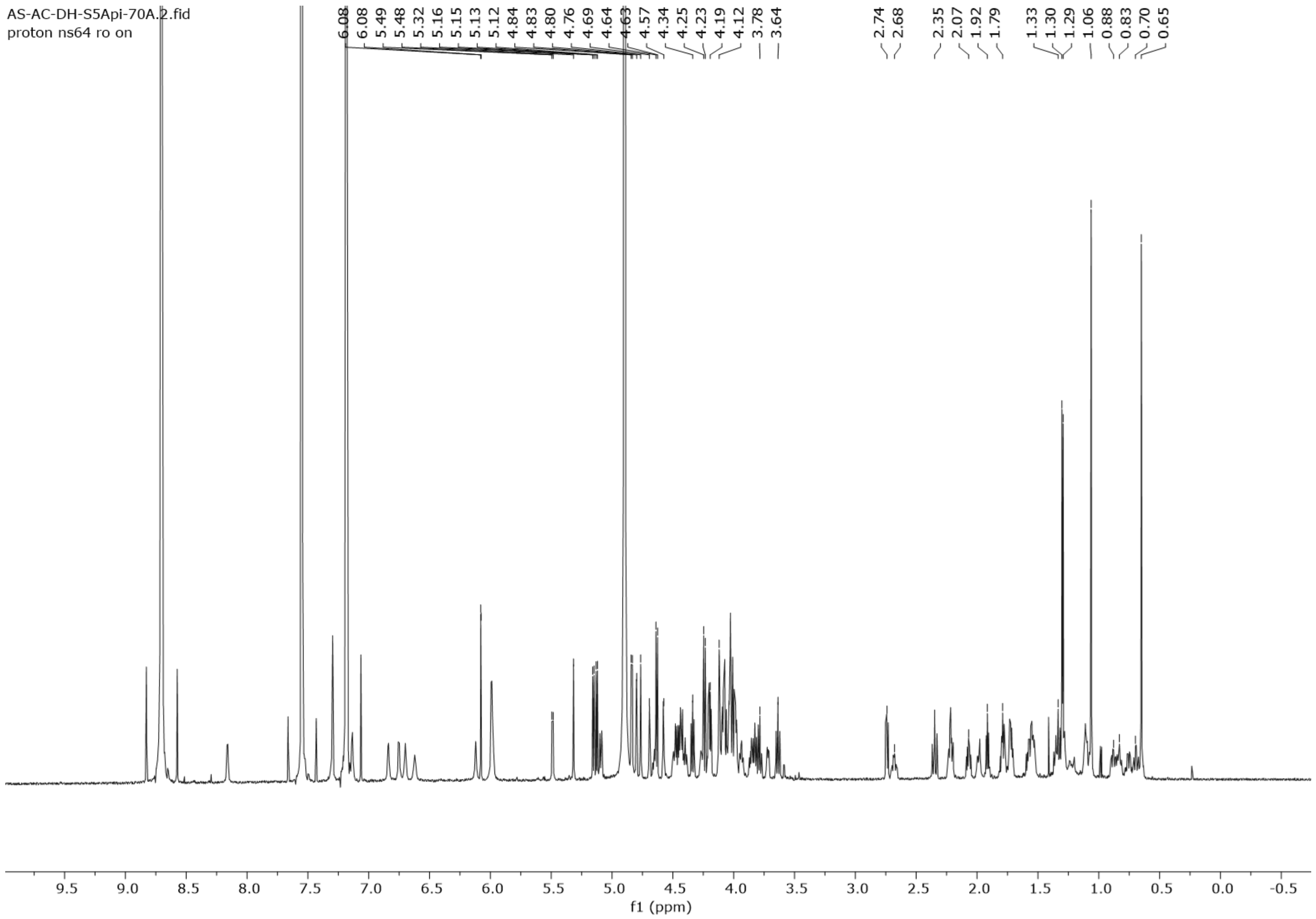

Figure S20. ^{1}H NMR spectrum of Coloratoside B (2) (700 MHz, Pyridine-d_5)

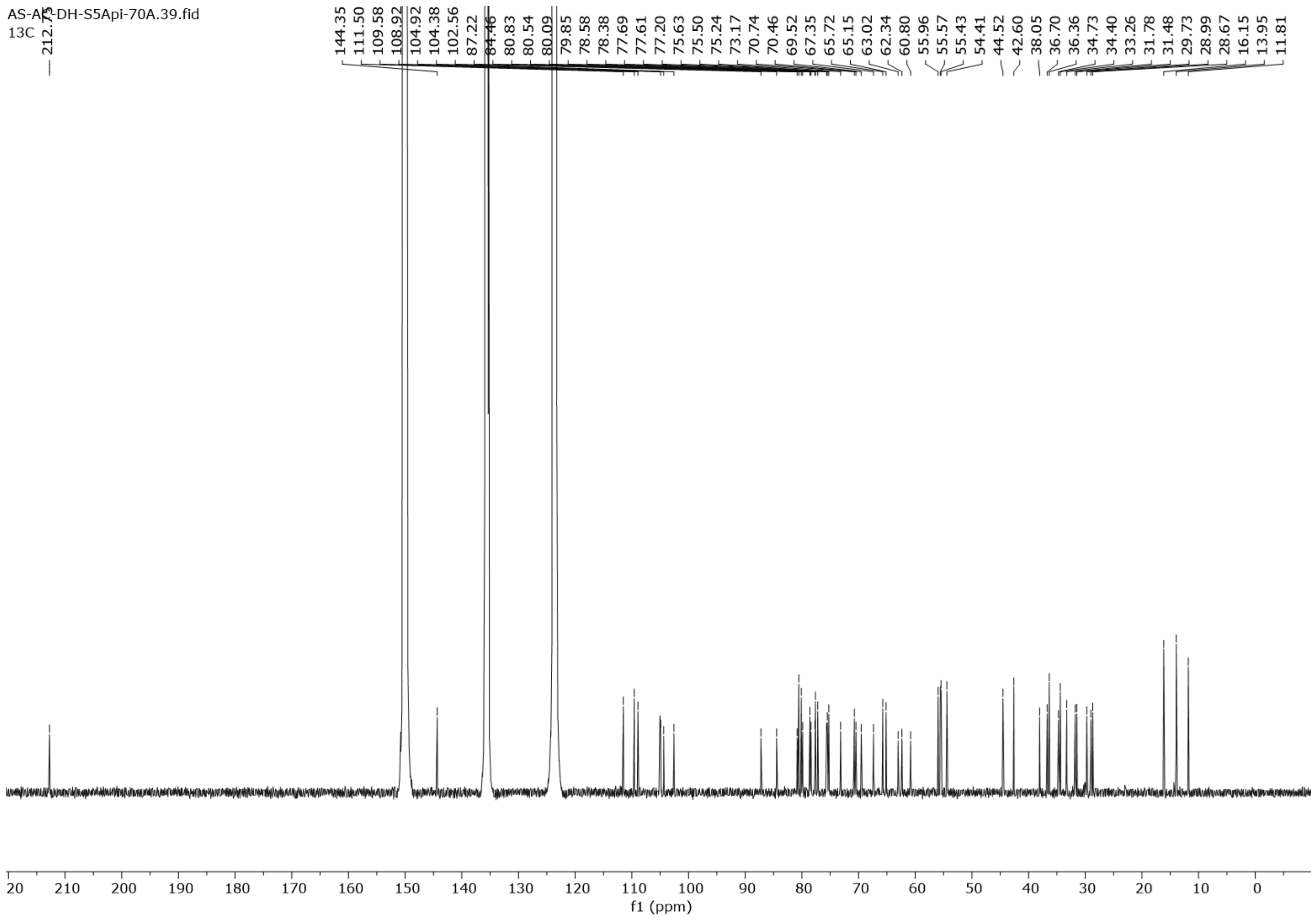

Figure S21. ^{13}C NMR spectrum of Coloratoside B (2) (700 MHz, Pyridine-d_5)

Elemental Composition Report **Page 1**

Tolerance = 5.0 mDa / DBE: min = -1.5, max = 50.0
Element prediction: Off
Number of isotope peaks used for i-FIT = 3

Monoisotopic Mass, Even Electron Ions
478 formula(e) evaluated with 3 results within limits (up to 5 best isotopic matches for each mass)
Elements Used:
C: 0-80 H: 0-1000 O: 0-200

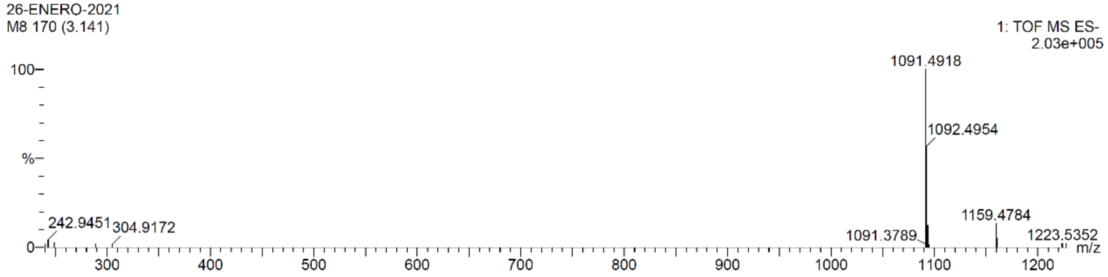

Minimum:	70.00				-1.5				
Maximum:	100.00		5.0	10.0	50.0				

Mass	RA	Calc. Mass	mDa	PPM	DBE	i-FIT	Norm	Conf(%)	Formula
1091.4918	100.00	1091.4910	0.8	0.7	12.5	27.7	0.073	93.00	C51 H79 O25
		1091.4946	-2.8	-2.6	34.5	30.5	2.879	5.62	C69 H71 O12
		1091.4887	3.1	2.8	43.5	31.9	4.285	1.38	C76 H67 O7

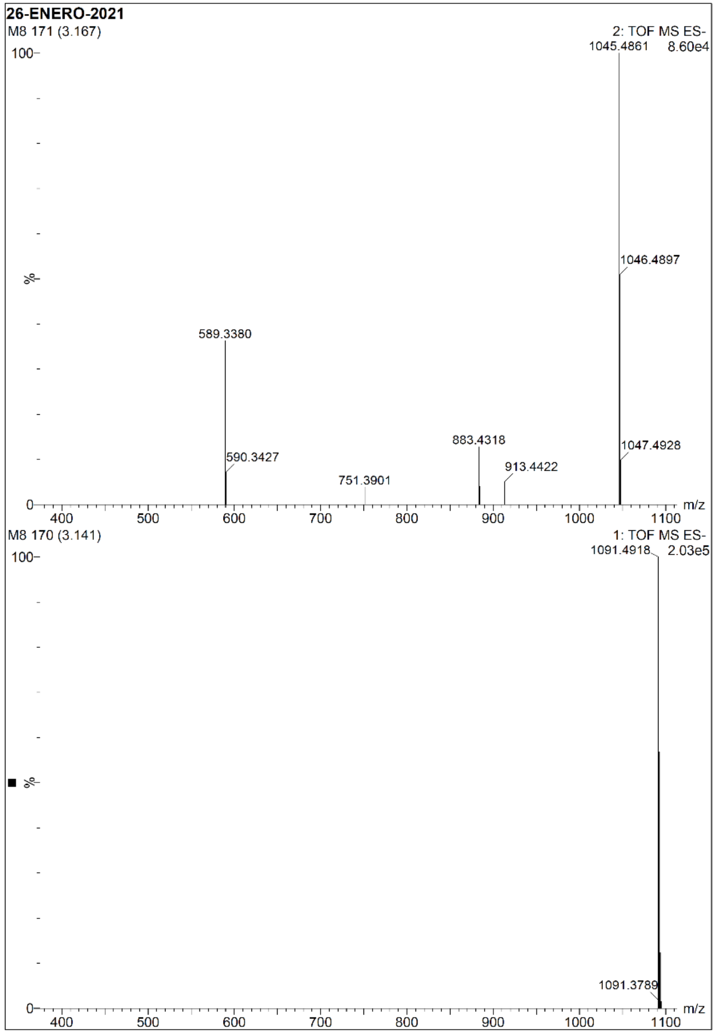

Figure S22. Elemental composition calculated for $C_{51}H_{79}O_{25}$ [M + CH_3COO^-]$^-$ and HRESI MS^E (negative mode) of Coloratoside C (3).

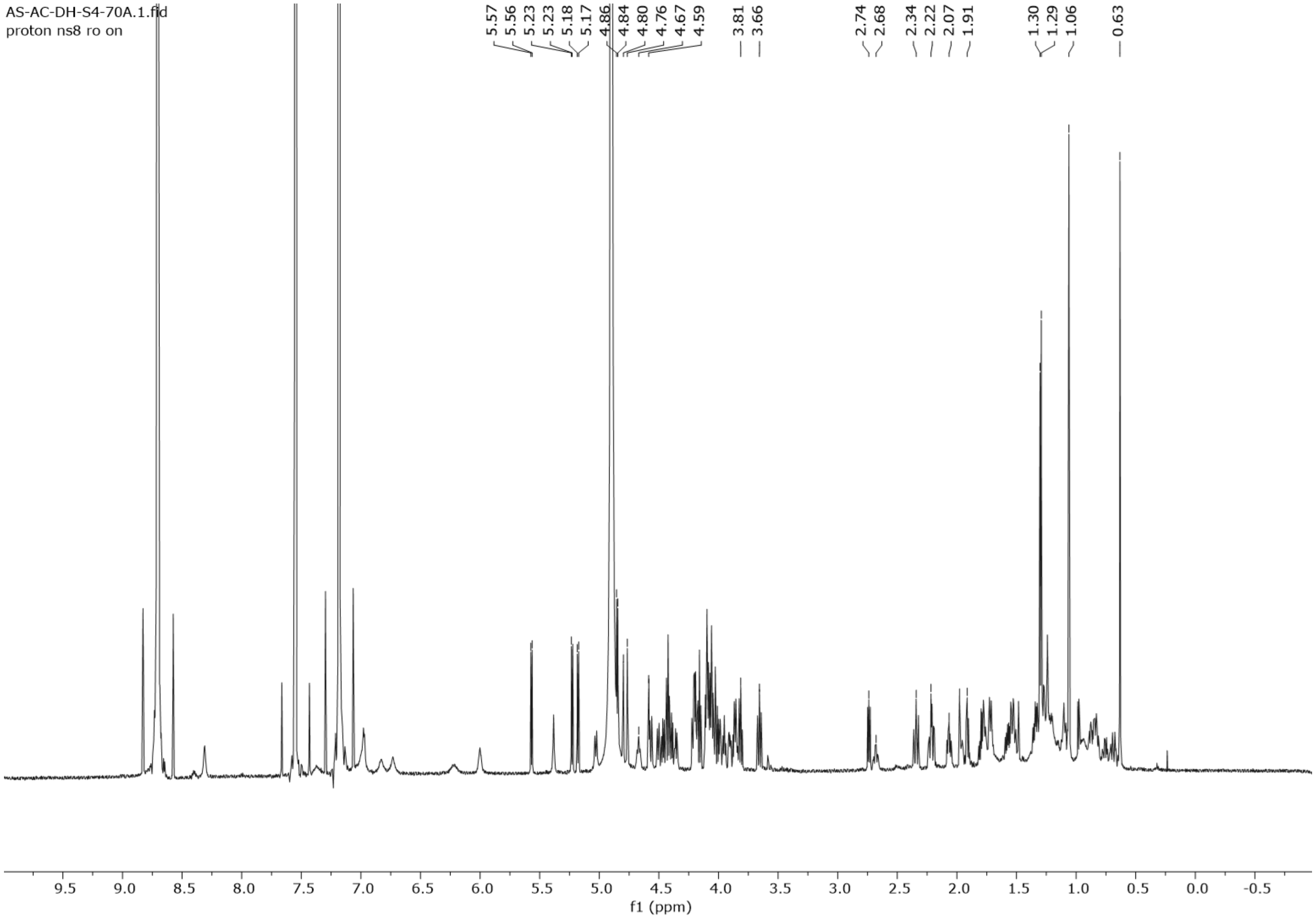

Figure S23. **^{1}H NMR spectrum of Coloratoside C (3) (700 MHz, Pyridine-d_5)**

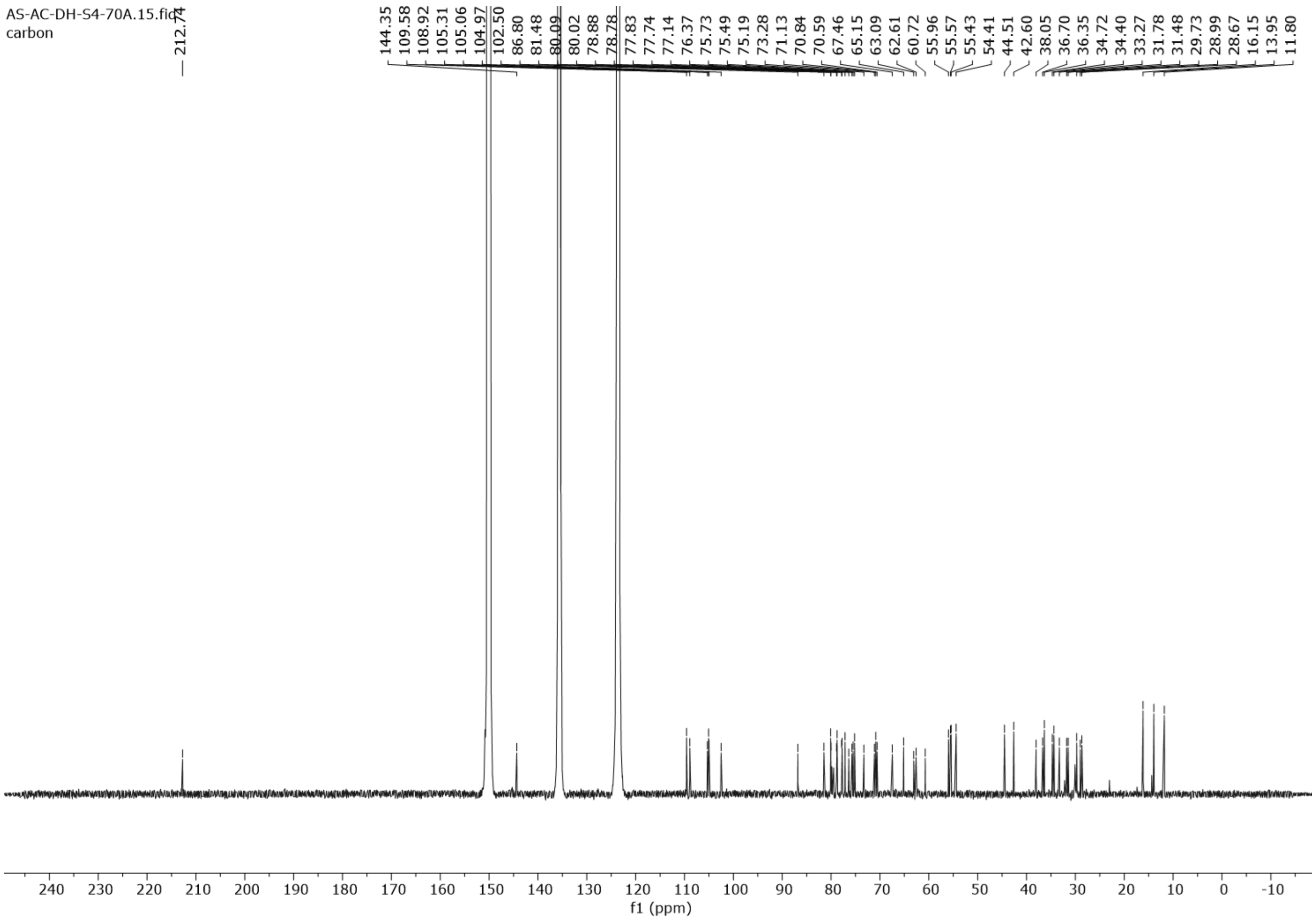

Figure S24. ^{13}C NMR spectrum of Coloratoside C (3) (700 MHz, Pyridine-d_5)

Elemental Composition Report

Tolerance = 5.0 mDa / DBE: min = -1.5, max = 50.0
Element prediction: Off
Number of isotope peaks used for i-FIT = 3

Monoisotopic Mass, Even Electron Ions
487 formula(e) evaluated with 3 results within limits (up to 5 best isotopic matches for each mass)
Elements Used:
C: 0-80 H: 0-1000 O: 0-200

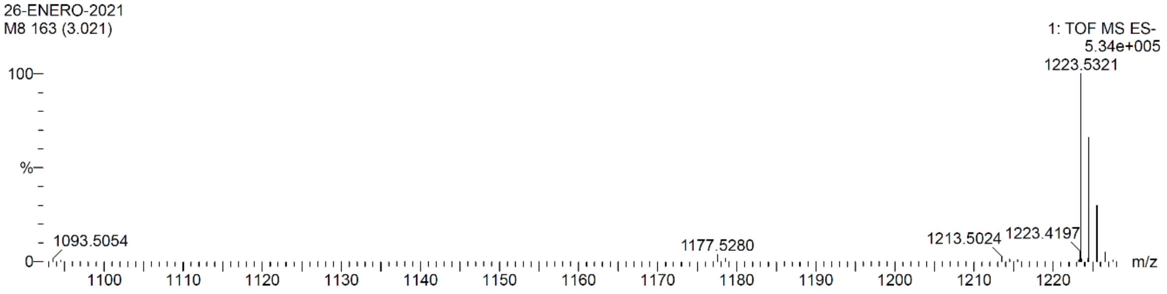

Minimum:	70.00				-1.5				
Maximum:	100.00		5.0	10.0	50.0				

Mass	RA	Calc. Mass	mDa	PPM	DBE	i-FIT	Norm	Conf(%)	Formula
1223.5321	100.00	1223.5333	-1.2	-1.0	13.5	80.0	0.443	64.20	C56 H87 O29
		1223.5274	4.7	3.8	22.5	80.6	1.039	35.37	C63 H83 O24
		1223.5368	-4.7	-3.8	35.5	85.0	5.442	0.43	C74 H79 O16

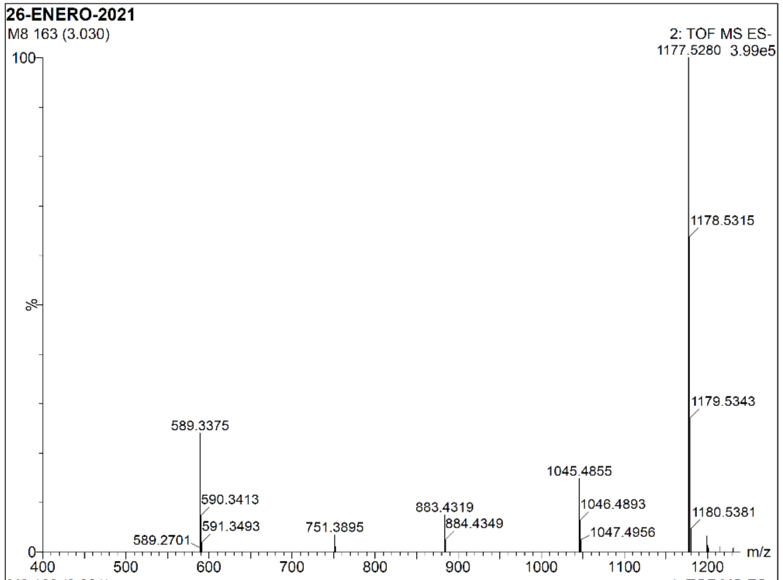

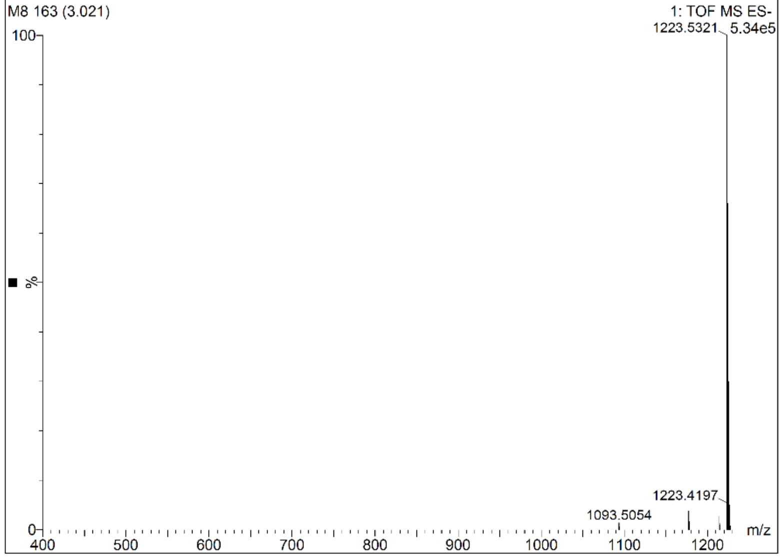

Figure S25. Elemental composition calculated for $C_{51}H_{79}O_{25}$ [M + CH_3COO^-]$^-$ and HRESI MS^E (negative mode) of Coloratoside D (4).

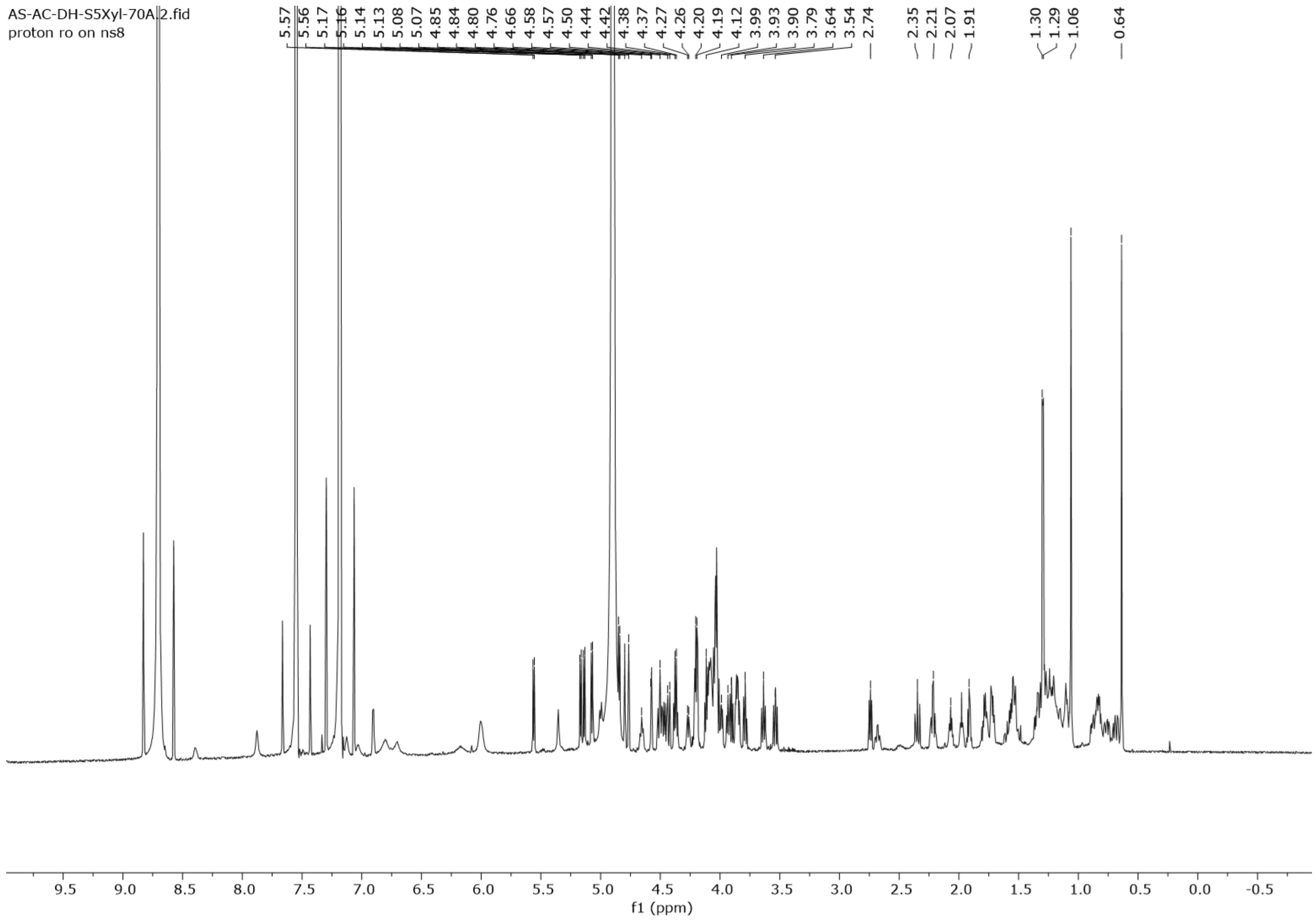

Figure S26. ^{1}H NMR spectrum of Coloratoside D (4) (700 MHz, Pyridine-*d*$_5$)

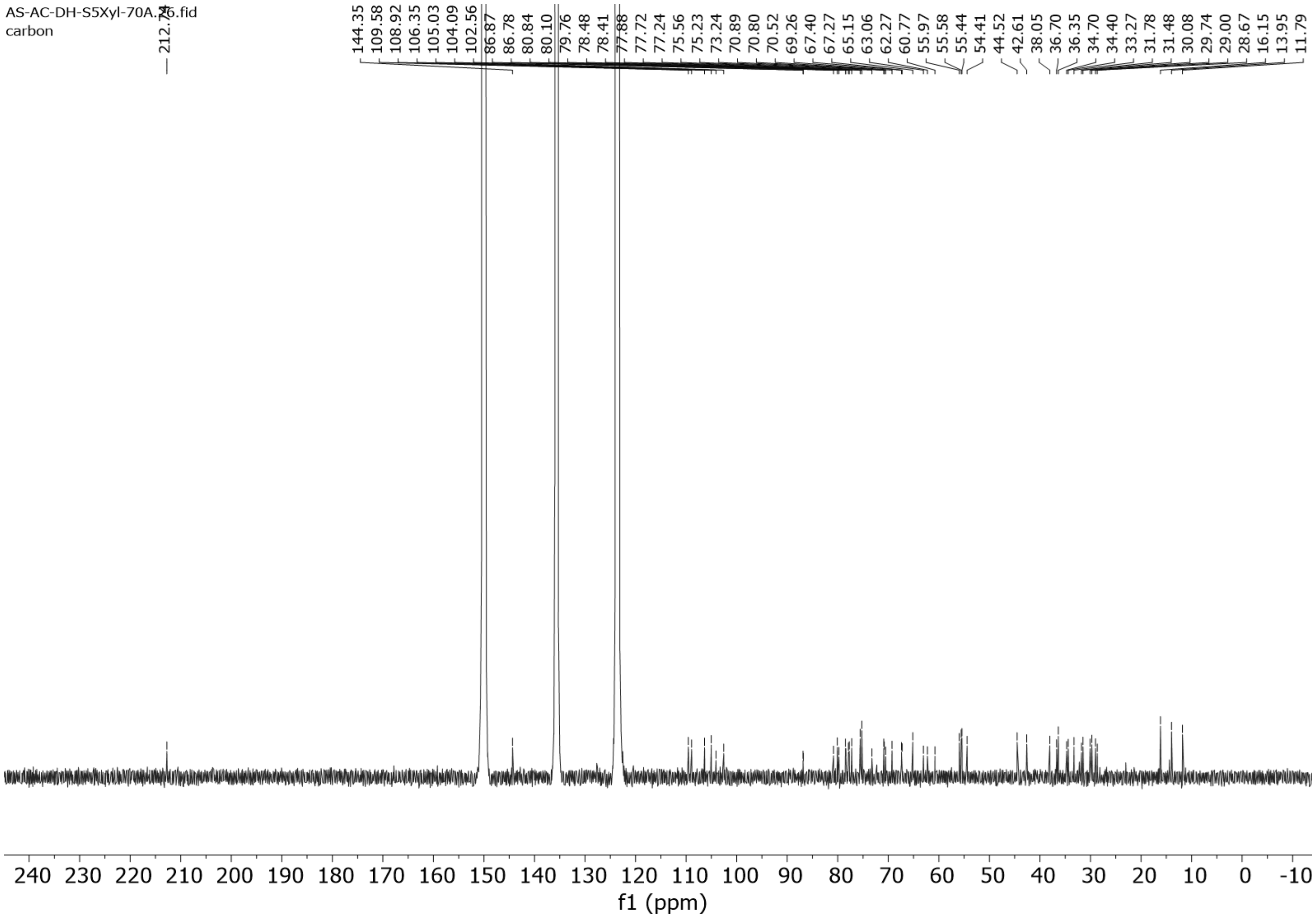

Figure S27. ^{13}C NMR spectrum of Coloratoside D (4) (700 MHz, Pyridine-d_5)

Elemental Composition Report

Tolerance = 5.0 mDa / DBE: min = -1.5, max = 50.0
Element prediction: Off
Number of isotope peaks used for i-FIT = 3

Monoisotopic Mass, Even Electron Ions
478 formula(e) evaluated with 3 results within limits (up to 5 best isotopic matches for each mass)
Elements Used:
C: 0-80 H: 0-1000 O: 0-200

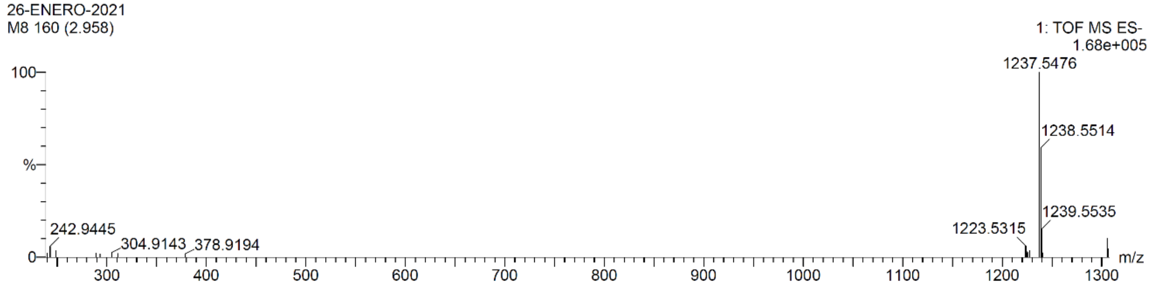

Minimum:	70.00				-1.5				
Maximum:	100.00		5.0	10.0	50.0				
Mass	RA	Calc. Mass	mDa	PPM	DBE	i-FIT	Norm	Conf(%)	Formula
1237.5476	100.00	1237.5490	-1.4	-1.1	13.5	27.6	0.038	96.25	C57 H89 O29
		1237.5431	4.5	3.6	22.5	31.0	3.451	3.17	C64 H85 O24
		1237.5525	-4.9	-4.0	35.5	32.7	5.148	0.58	C75 H81 O16

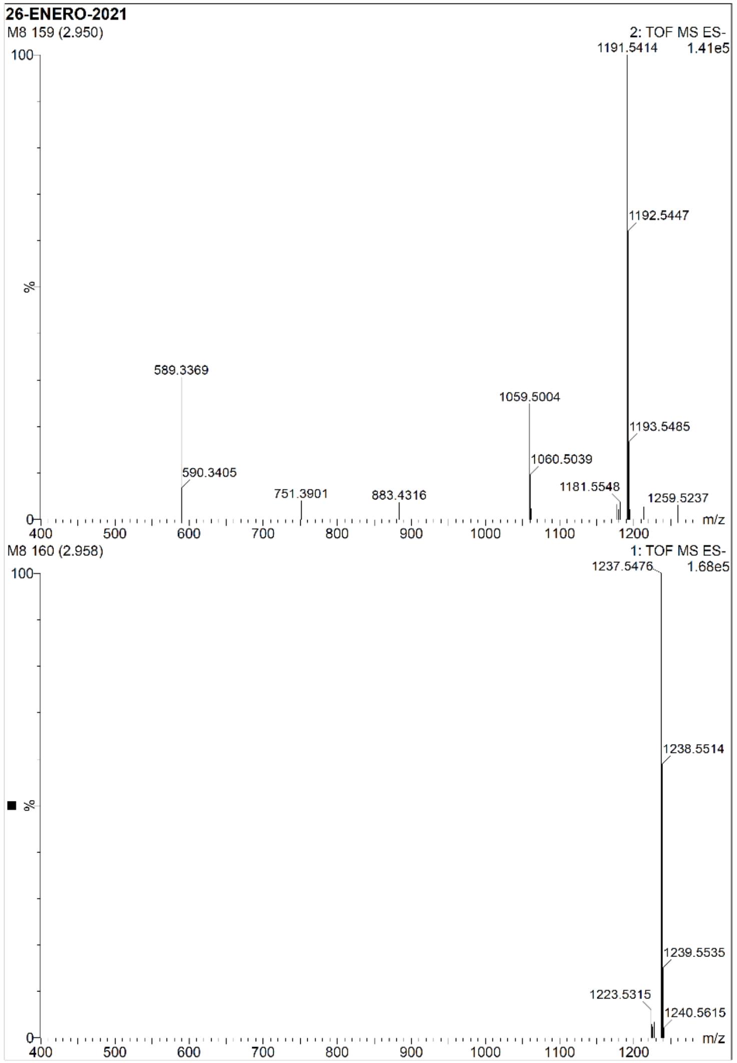

Figure S28. Elemental composition calculated for $C_{51}H_{79}O_{25}$ [M + CH_3COO^-]$^-$ and HRESI MS^E (negative mode) of Coloratoside E (5).

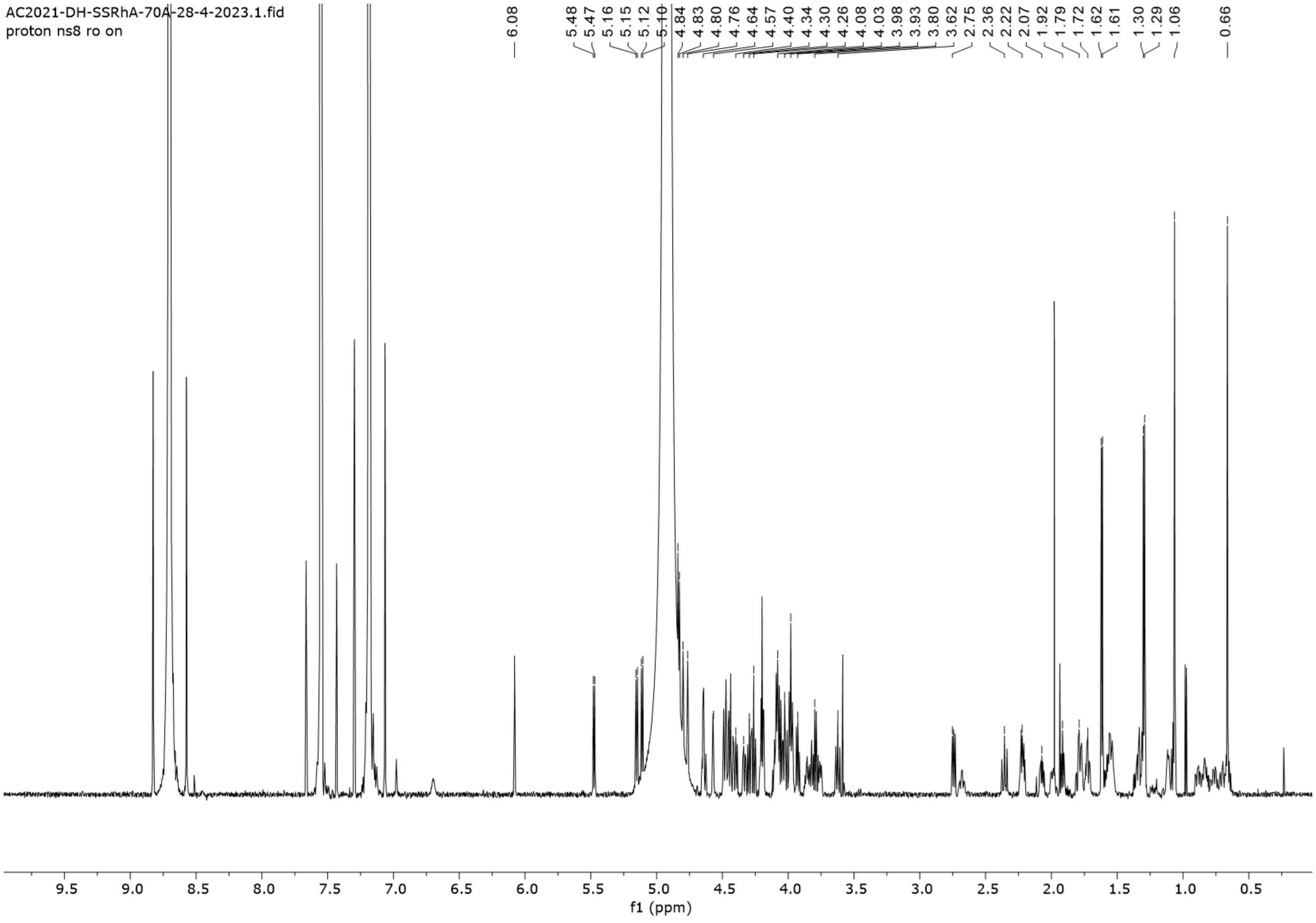

Figure S29. ^{1}H NMR spectrum of Coloratoside E (5) (700 MHz, Pyridine-d_5)

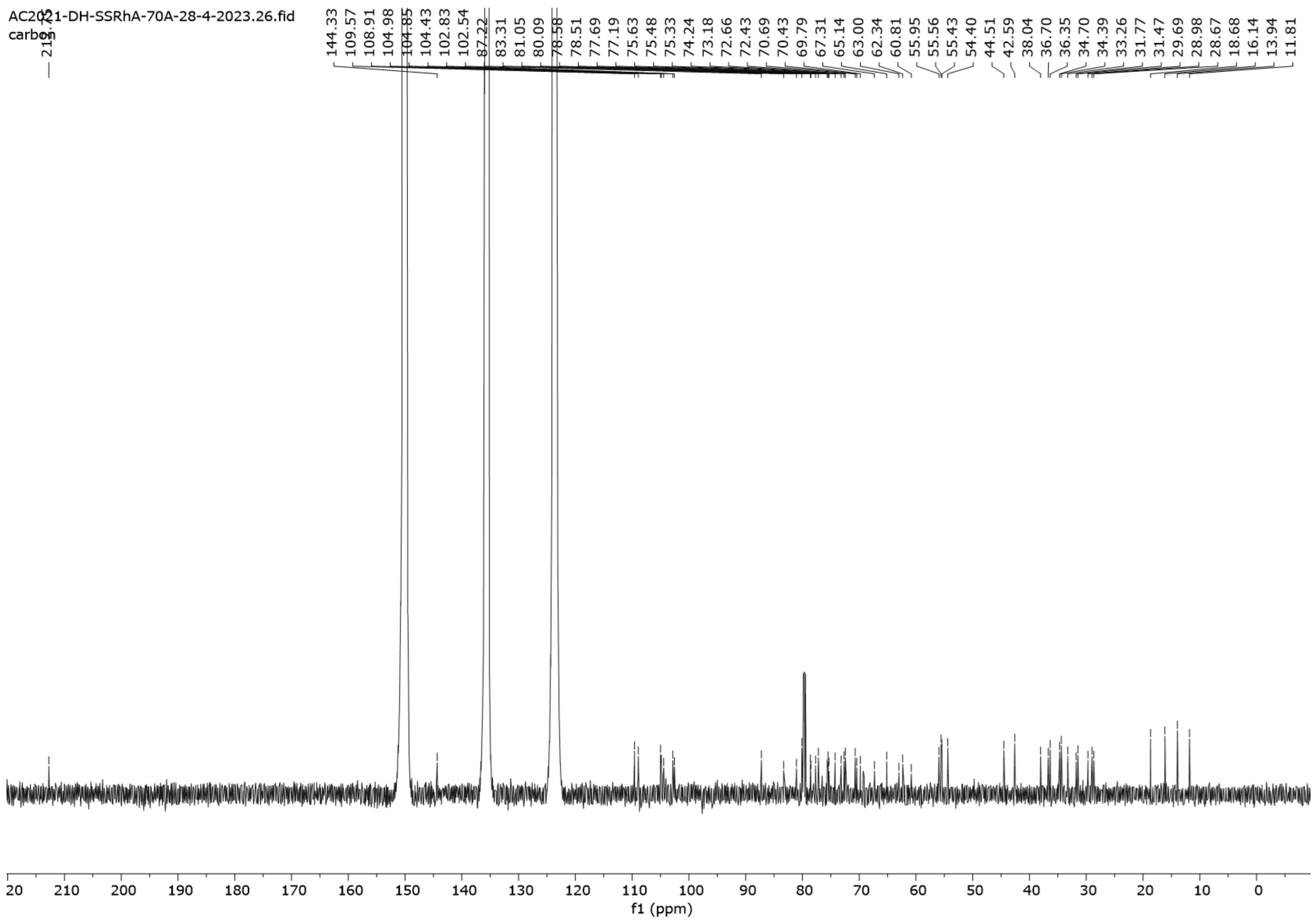

Figure S30. ^{13}C NMR spectrum of Coloratoside E (5) (700 MHz, Pyridine-d_5)

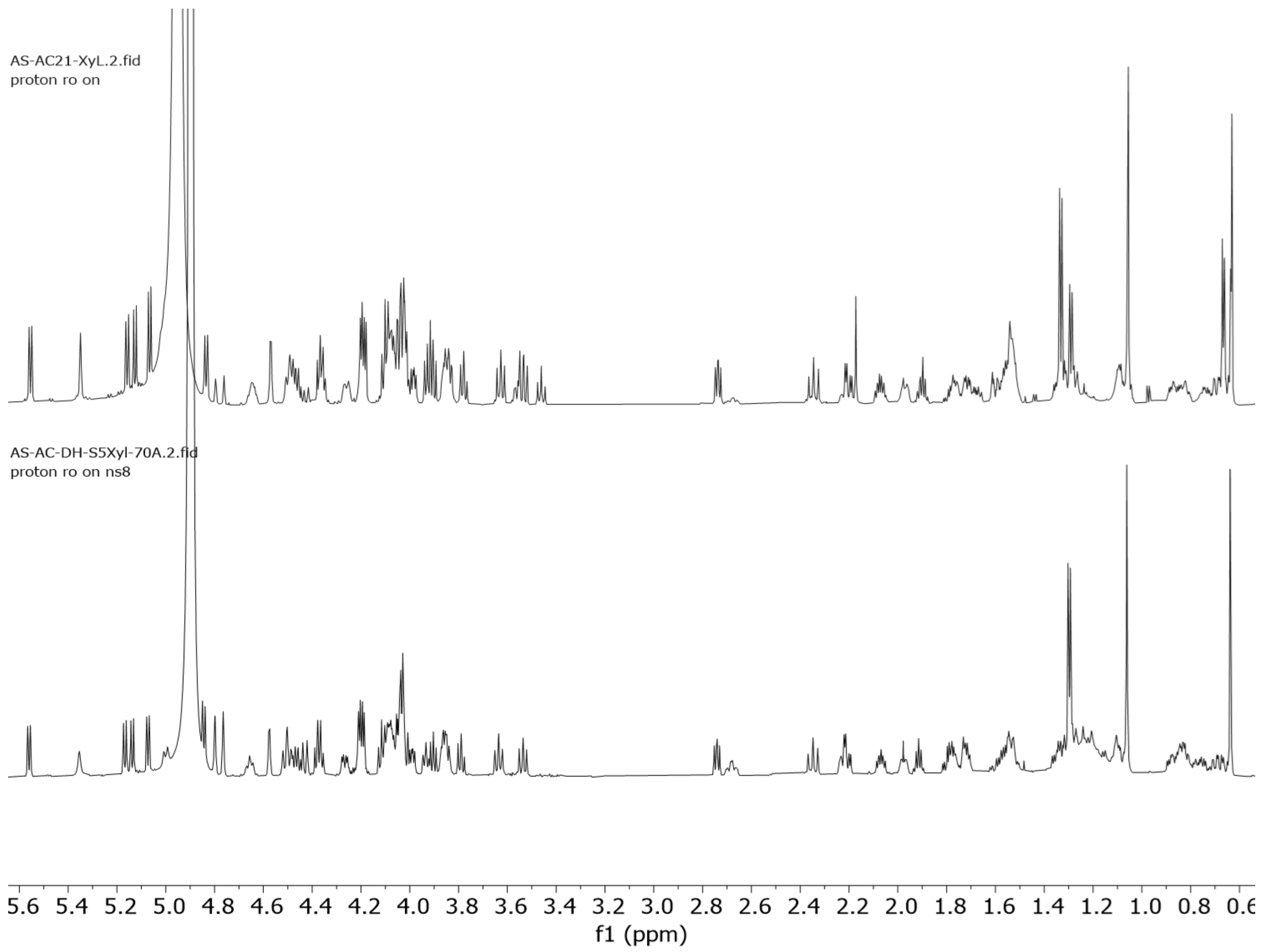

Figure S31. Example of ^{1}H NMR spectra comparison: Coloratoside D (4) and previous fraction (60% of 7 and 40% of 4) with the same structure of sugar chain for both compounds (700 MHz, Pyridine-d_5)

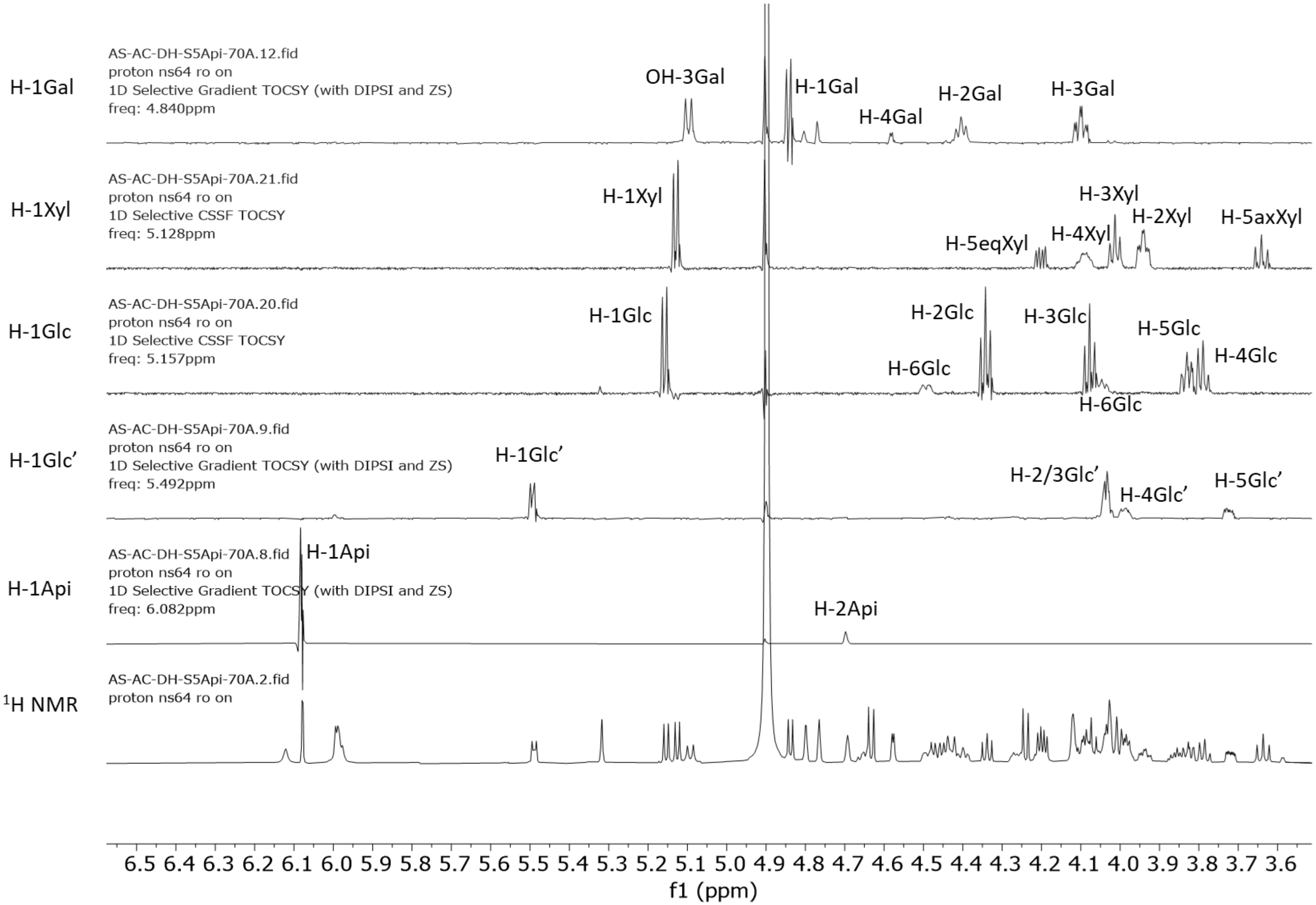

Figure S32. Example of 1D TOCSY spectra (120 ms): Sugar chain of Coloratoside B (2). (700 MHz, Pyridine-d_5)

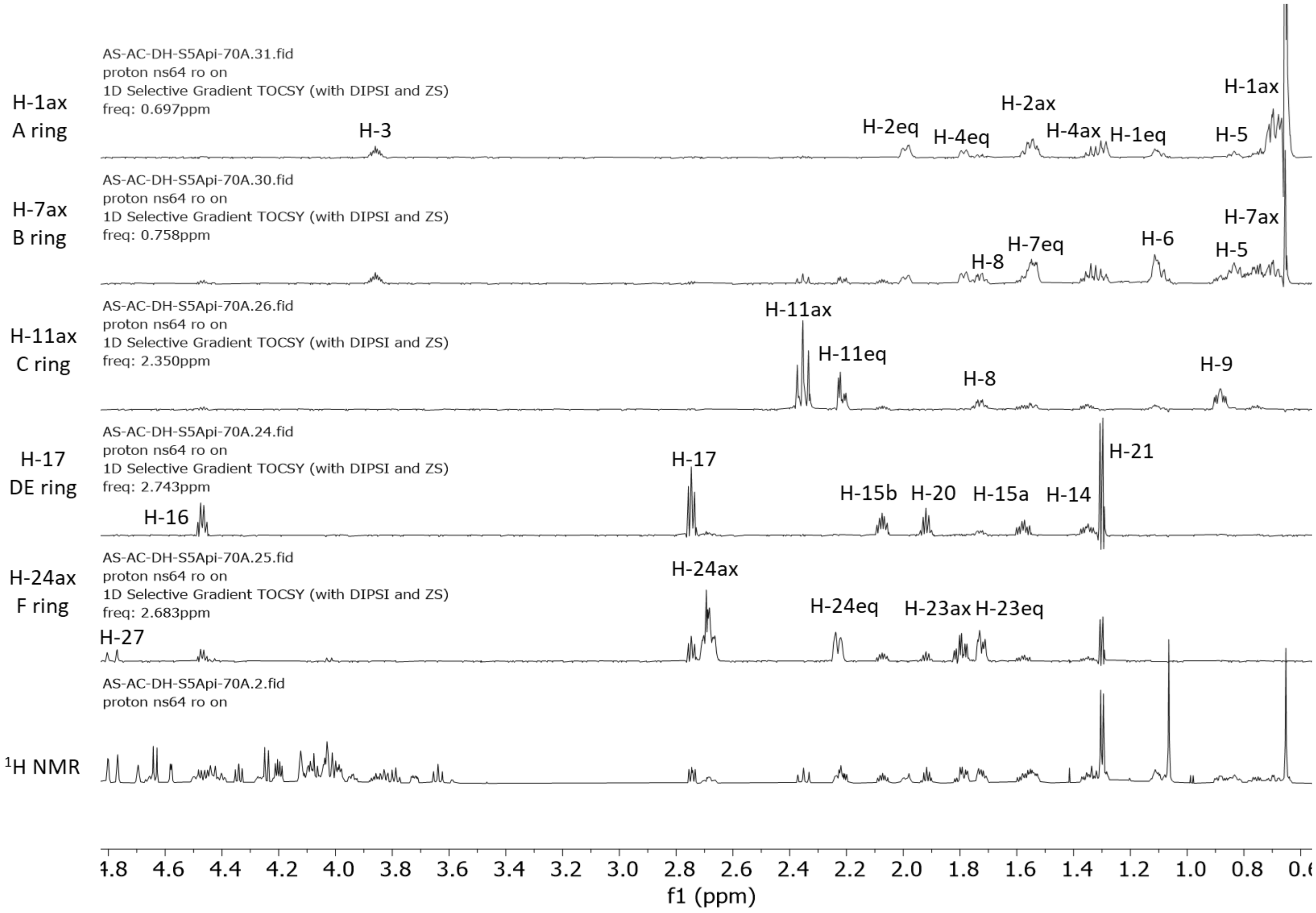

Figure S33. Example of 1D TOCSY spectra (120 ms): Aglycone of Coloratoside B (2). (700 MHz, Pyridine-d_5)
